# New Oligophenalenone Dimers from *Talaromyces stipitatus* with Potential Anti-Neuroinflammatory Activity

**DOI:** 10.3390/molecules31081308

**Published:** 2026-04-17

**Authors:** Qing Liu, Yu Gao, Biaopeng Wang, Kehan Du, Shengmin Zhu, Yahong Yuan, Yuqi Gao, Tianli Yue

**Affiliations:** 1 Shaanxi Key Laboratory of Food Safety in Higher Education Institutions, College of Food Science and Technology, Northwest University, Xi’an 710069, China; chenliuqingq@163.com (Q.L.); gaoyuyouxiang@yeah.net (Y.G.); bpwang@stumail.nwu.edu.cn (B.W.); dukehan@163.com (K.D.); junrouovo@outlook.com (S.Z.); 2 Selenium-Enriched Tea Research and Development Center, Northwest University, Xi’an 710069, China

**Keywords:** oligophenalenone, *Talaromyces*, anti-neuroinflammatory, Alzheimer’s disease

## Abstract

Five new oligophenalenone dimers, talarostipins A–E (**1**–**5**), including four N-containing derivatives, together with sixteen known duclauxin analogues (**6**–**21**), were isolated from the soil fungus *Talaromyces stipitatus*, and their structures were identified by a combination of NMR spectroscopic analyses, HRESIMS and ECD spectra. Anti-neuroinflammatory activity evaluation indicated that duclauxamide C (**8**) significantly inhibited NO generation in lipopolysaccharide (LPS)-induced BV-2 microglial cells with an IC_50_ value of 5.0 ± 0.7 μM. Transcriptome sequencing analysis indicated that **8** probably suppressed neuroinflammation by targeting the NF-*κ*B signaling pathway. Further verification was conducted by Western blot analysis, which indicated that **8** exerted its anti-neuroinflammatory effect by downregulating the expression of pro-inflammatory proteins, including inducible nitric oxide synthase (iNOS), cyclooxygenase-2 (COX-2) and p65.

## 1. Introduction

Alzheimer’s disease (AD), the most prevalent neurodegenerative disease (NDD) worldwide, is characterized by progressive cognitive decline and persistent neuroinflammation [1]. Among these pathological hallmarks, neuroinflammation has emerged as a pivotal driver of AD progression, rather than a mere secondary response, with accumulating evidence highlighting its role in accelerating neuronal damage and cognitive impairment [2,3]. Microglial activation, the hallmark of neuroinflammation in AD, triggers the overproduction of pro-inflammatory mediators and activates downstream inflammatory signaling pathways, creating a self-sustaining vicious cycle that exacerbates disease pathology [4]. Thus, modulating neuroinflammation has become a prospective therapeutic target for AD, and the development of novel anti-neuroinflammatory agents with high efficacy and low toxicity remains an urgent clinical need [5]. Inflammatory signaling pathways play a critical role in the pathogenesis of AD-related neuroinflammation, with the NF-*κ*B signaling pathway standing out as a central regulator [6]. The NF-*κ*B pathway orchestrates the transcription of pro-inflammatory cytokines, chemokines, and inflammatory enzymes, and its dysregulated activation is closely associated with microglial overactivation and AD progression [7,8]. Previous studies have shown that targeting the NF-*κ*B pathway can effectively suppress neuroinflammation, reduce Aβ deposition and tau hyperphosphorylation, and alleviate neuronal damage in AD models [9,10].

**Figure 1:**
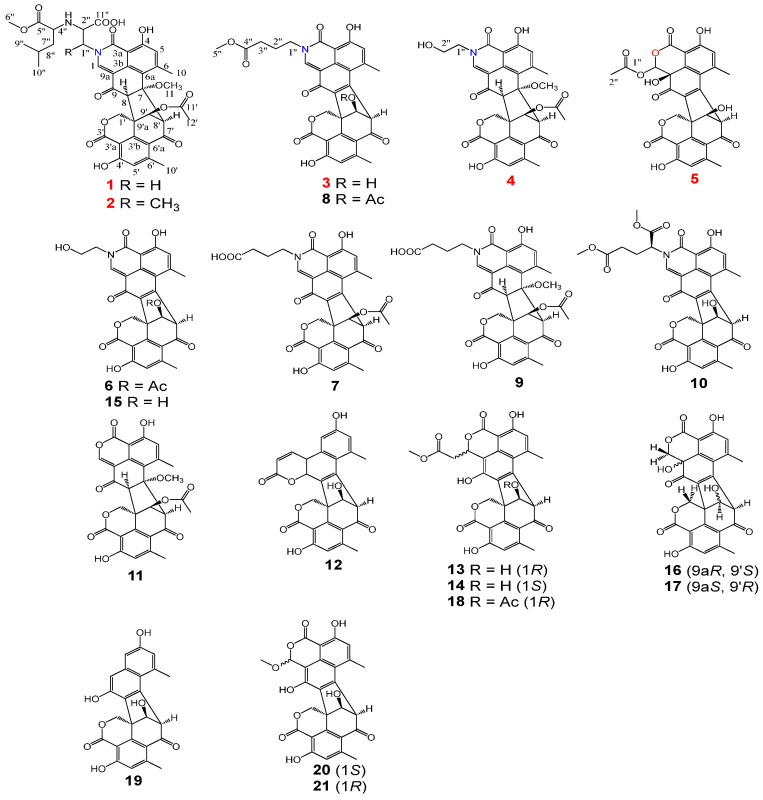
Structures of compounds **1**–**21** from *T. stipitatus*.

Natural products derived from microbial sources have long been recognized as indispensable reservoirs for drug discovery, owing to their structural diversity, unique stereochemical features, and diverse pharmacological activities, which could rarely be replicated by synthetic compounds [11]. Fungi, particularly those inhabiting specialized ecological niches such as soil and marine environments, are prolific producers of secondary metabolites with distinct biosynthetic origins, including polyketides, meroterpenoids, alkaloids, and oligophenalenone derivatives [12]. Among fungal genera, *Talaromyces*, initially classified as the sexual type of Penicillium and later established as an independent taxon, has emerged as a prominent source of bioactive secondary metabolites, attracting growing attention in medicinal chemistry and pharmacology research [13]. Previous work on the discovery of secondary metabolites from *Talaromyces*, such as bacillisporins F–H [14], talauromides A–G [15], talaroclauxins A–B [16], and bacillisporins K–L [17], mainly concentrates on their antibacterial activities. Thus, the present study aims to explore the diverse structures of oligophenalenone dimers of *T. stipitatus*, which were obtained from soil samples collected in Taibai Mountain, with a particular focus on their anti-neuroinflammatory activities. The investigation of the fermentation of *T. stipitatus* led to the isolation of five novel duclauxin analogues, talarostipins A–E (**1**–**5**), along with sixteen known oligophenalenone dimers (Figure 1). Bioactivity evaluations revealed that duclauxamide C (**8**) displayed the most significant activity (IC_50_ 5.0 μM) against neuroinflammation in LPS-induced BV-2 microglial cells. Further exploration clarified that **8** is a potential agent for the treatment of AD by targeting the NF-*κ*B signaling pathway.

## 2. Results and Discussion

Talarostipin A (**1**) was obtained as an orange solid with the molecular formula C_39_H_40_N_2_O_14_ on the basis of NMR (Table 1) and HRESIMS data (*m*/*z* 761.2542 [M + H]^+^, calcd for C_39_H_41_N_2_O_14_, 761.2558), indicating the presence of 21 degrees of unsaturation. According to the ^1^H NMR spectrum together with HSQC of **1**, five methyl groups [*δ*_H_ 2.70 (s, 3H), 2.23 (s, 3H), 2.05 (s, 3H), 0.95 (overlap, 3H), 0.95 (overlap, 3H)], two methoxyl groups [*δ*_H_ 3.80 (s, 3H), 2.96 (s, 3H)], one methylene group [*δ*_H_ 1.62 (m, 2H)], two oxymethylene groups [*δ*_H_ 5.10 (d, *J* = 12.0 Hz, 1H)/4.73 (d, *J* = 12.0 Hz, 1H), 4.07 (m, 1H)/4.21 (m, 1H)], six methine groups [*δ*_H_ 5.31 (t, *J* = 4.0 Hz, 1H), 5.20 (s, 1H), 4.66 (m, 1H), 4.14 (s, 1H), 4.01 (s, 1H), 1.66 (m, 1H)], three aromatic proton signals [*δ*_H_ 8.02 (s, 1H), 6.76 (s, 1H), 6.50 (s, 1H)], two phenolic hydroxyl groups [*δ*_H_ 12.07 (s, 1H), 11.58 (s, 1H)] and one secondary amine group [*δ*_H_ 6.71 (m, 1H)] were distinguished. The ^13^C NMR spectrum revealed 39 carbon signals corresponding to seven methyl groups (including two oxygenated at *δ*_c_ 52.88, 51.73), three methylene groups (including two oxygenated at *δ*_c_ 71.51, 60.11), nine methine groups (including three sp^2^ methine carbons at *δ*_c_ 135.92, 121.24, 118.45), and twenty quaternary carbon signals (including seven carbonyl groups at 194.41, 191.23, 174.15, 169.79, 167.42, 167.03, 165.29, eleven aromatic or olefinic carbons and two sp^3^ carbons). As **1** had 21 degrees of unsaturation, and seven carbonyl groups and seven olefinic bonds (14 sp^2^ carbons) accounted for 14 degrees of unsaturation, **1** should bear seven rings.

**Figure 2:**
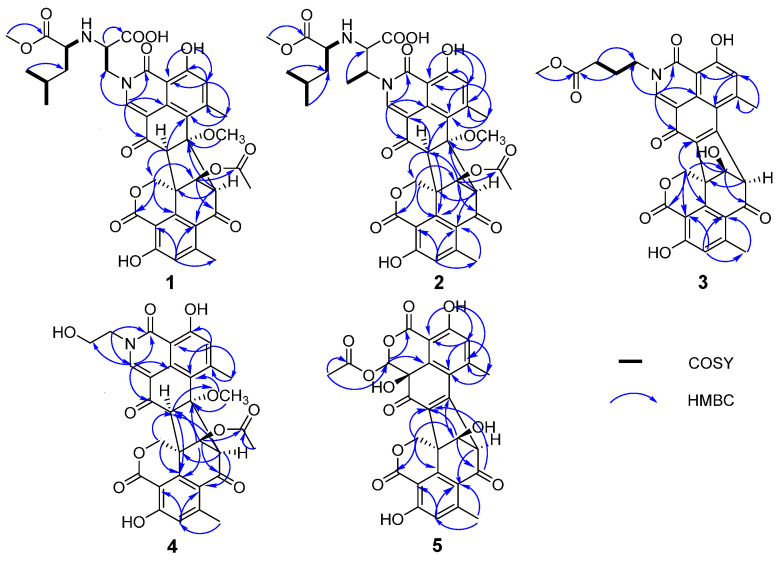
Key ^1^H–^1^H COSY and HMBC correlations of **1**–**5**.

**Figure 3:**
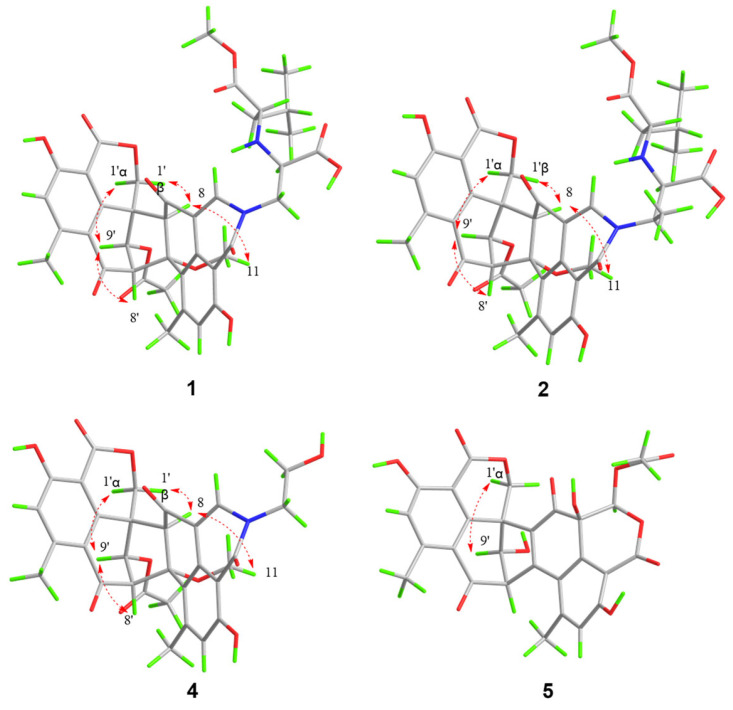
Key NOESY correlations of **1**–**2** and **4**–**5**.

The planar structure of **1** was clarified based on 2D NMR data (Table 1, Figure 2 and Figure 3), which exhibited similarity to talauxins [18], derivatives of duclauxin, except for the N-2 side chain. The ^1^H–^1^H COSY correlations between H_α_-1″/H-2″, N-3″/H_1_-4″, H_1_-4″/H_2_-7″, H_3_-9″/H_1_-8″, H_1_-8″/H_3_-10″, together with the HMBC correlations from H_3_-6″ to C-5″, from H_3_-9″ to C-7″, from H_1_-2″ to C-11″, from H_1_-1 to C-1″, and the chemical shifts of C-1″ at *δ*_c_ 60.11 and C-2″ at *δ*_c_ 61.57 elucidated the (1-methoxy-4-methyl-1-oxopentan-2-yl)alanine moiety at N-2. The relative configuration of **1** was confirmed by the NOESY spectrum. The correlations of H_1_-8/H_β_-1′, H_1_-8/H_3_-11, H_1_-9′/H_α_-1, H_1_-8′/H_1_-9′ and the small coupling between H_1_-8′/H_1_-9′ were consistent with duclauxin [19], indicating that the partial relative configuration of **1** was assigned as (7S*, 8S*, 8′S*, 9′S*, 9a′R*)—1.

**Figure 4:**
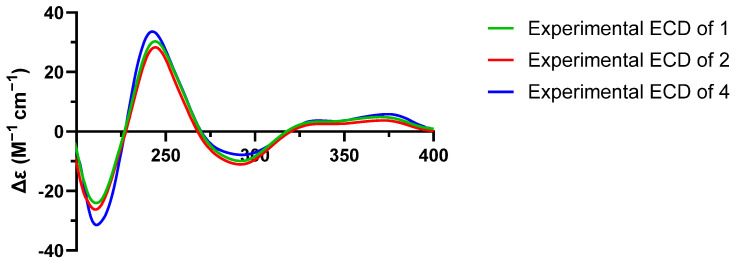
Experimental ECD spectra of compounds **1**, **2** and **4**.

Talarostipin B (**2**), isolated as an orange solid, possessed the molecular formula C_40_H_42_N_2_O_14_ according to HRESIMS data (*m*/*z* 775.2704 [M + H]^+^, calcd for C_40_H_43_N_2_O_14_, 775.2714). Comparison of the NMR spectra (Table 2) of **1** and **2** indicated that they had the same skeleton, with the only difference being that a methyl group was connected to C-1″ (Figure 1), which could be validated by the COSY correlations between H_1_-1″/C-12″ and H_1_-1″/H_1_-2″, together with the HMBC correlation from H-12″ to C-2″. The key NOESY correlations of H_1_-8/H_3_-11, H_1_-8/H_β_-1′, H_1_-8′/H_1_-9′ and H_1_-9′/H_α_-1′ were the same as those of **1**, indicating that the two compounds shared the identical partial (7S*, 8S*, 8′S*, 9′S*, 9a′R*) configuration. The absolute configurations of the 6/6/6/5/6/6/6 heptacyclic skeletons in **1** and **2** were established as 7*S*, 8*S*, 8′*S*, 9′*S*, and 9a′*R* based on their experimental ECD spectra (Figure 4), which showed almost the same Cotton effects as those of duclauxin [20]. Despite extensive efforts employing various methods, including DP4+ probability analysis, the configuration of the N-2 side chain could not be unambiguously determined.

**Figure 5:**
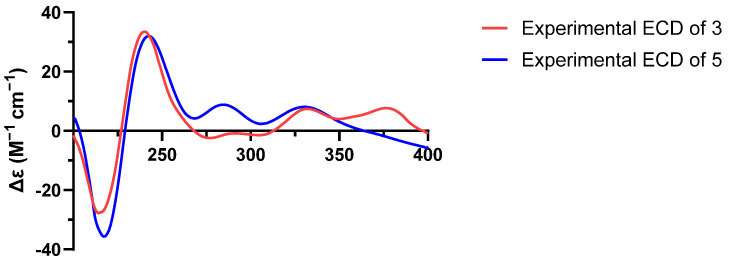
Experimental ECD spectra of compounds **3** and **5**.

Talarostipin C (**3**) was purified as an orange solid and the molecular formula was revealed as C_31_H_25_NO_10_ by HRESIMS (*m*/*z* 572.1548 [M + H]^+^, calcd for C_31_H_26_NO_10_, 572.1557). The 1D and 2D NMR data of **3** resembled those of duclauxamide C (**8**) [21]. Further elaborate analysis indicated that the upfield shift of H_1_-9′ (*δ*_H_ 4.85 in **3**; *δ*_H_ 5.73 in **8**), and the absence of corresponding signals for the acetyl group (9′-OCOCH_3_ at *δ*_c_ 170.24, 9′-OCOCH_3_ at *δ*_c_ 20.99 and 9′-OCOCH_3_ at *δ*_H_ 2.08), in comparison to **8,** established the replacement of the acetyloxy unit at C-9′ in **8** by a hydroxyl group in **3** (Table 2, Figure 1). The absolute configuration of compound **3** was established as 8′*R*, 9′*S*, and 9a′ *S* by comparison of its experimental ECD spectrum (Figure 5) with that of talauromide C [15], as well as the almost identical NMR data at C-8′, C-9′ and C-9a′, and biogenetic considerations.

Talarostipin D (**4**) was isolated as a yellow solid, and its molecular formula C_31_H_27_O_11_ was assigned from the HRESIMS [M + H]^+^ peak at *m*/*z* 590.1652 (calcd for C_31_H_28_NO_11_, 590.1662). The analysis of NMR data suggested that the core skeleton of **4** was identical to that of **1**, apart from the side chain at N-2. Two methylene signals at *δ_C_* 60.70 (*δ*_H_ 3.87, H_α_-1″; *δ*_H_ 4.12, H_β_-1″) and 50.01 (*δ*_H_ 3.87, H_2_-2″) revealed the presence of a hydroxyethyl group at N-2, consistent with duclauxamide A1 [22], instead of the alanine moiety in **1**. This was confirmed by the HMBC correlations from H_β_-1″ to C-1/C-3 and from H_1_-1 to C-2″ (Table S4). The key NOESY correlations of H_1_-8/H_3_-11, H_1_-8/H_β_-1′, H_1_-8′/H_1_-9′ and H_1_-9′/H_α_-1′ were the same as those of **1**, indicating that the two compounds shared the identical partial (7S*, 8S*, 8′S*, 9′S*, 9a′R*) configuration. The comparison of the CD spectrum (Figure 4) with that of duclauxin [20] determined that the absolute configuration of **4** was 7*S*, 8*S*, 8′*S*, 9′*S*, 9a′*R*.

Talarostipin E (**5**) was a yellow solid, and the HRESIMS displayed a molecular peak at *m*/*z* 549.1030 [M + H]^+^ (calcd for C_28_H_21_NO_12_, 549.1033), deducing its molecular formula as C_28_H_20_O_12_. The ^1^H and ^13^C NMR spectra of **5** showed structural similarities with Taladuxin C (**16**) [23], except for the additional acetyl group (C-1″ at *δ*_C_ 170.22, C-2″ at *δ*_C_ 36.57 and H_3_-2″ at *δ*_H_ 2.05) linked at C-1 (C-1 at *δ*_C_ 80.33, H_1_-1 at *δ*_H_ 5.44) (Table 2), which was further attested by the HMBC correlations from H_3_-2″ to C-1″ and C-1 (Table S5). The relative configuration of **5** was assigned as 8′R*, 9′S*, 9a′S* based on the key NOESY signals H_1_-9′/H_α_-1′ and the nearly same NMR data as taladuxin D [23] at C-8′, C-9′ and C-9a′. Along with the experimental ECD spectra (Figure 5) of **5** and taladuxin D [23], biogenetic considerations suggested the absolute configuration of **5** to be 8′*R*, 9′*S*, and 9a′*S*.

The remaining known compounds were identified as duclauxamides A1–C (**6**–**8**), talaroclauxin C (**9**), talauromide (**10**), duclauxin (**11**), adpressins A–C and F (**12**–**15**), taladuxins C–D and J (**16**–**18**), macrosporusone A (**19**), and macrosporusones D (**20**–**21**, 1S and 1R) [18,19,20,21,22,23] by comparing the NMR spectroscopic data with those in the literature.

**Figure 6:**
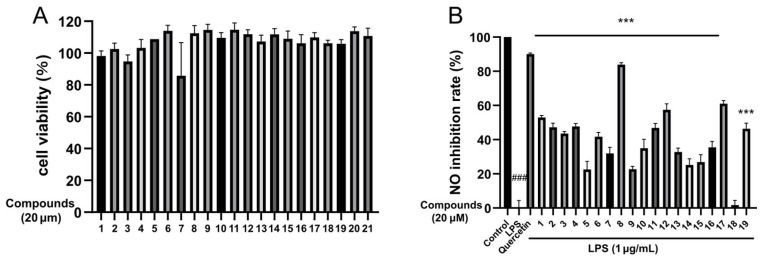
(**A**) The effect of the compounds on BV-2 cell viability at a concentration of 20 μM. (**B**) The effect of the compounds on NO production in LPS-induced BV-2 cells at a concentration of 20 μM. All values are the mean ± SEM of three independent experiments. Compared with the control group, ^###^ *p* < 0.05; Compared with the model group (LPS), *** *p* < 0.001.

Mounting clinical and preclinical evidence has demonstrated that neuroinflammation is not only a secondary response to AD pathology but also an independent therapeutic target, and interventions targeting neuroinflammatory pathways are expected to delay or reverse disease progression [5]. To evaluate the anti-neuroinflammatory potential of these compounds, compounds **1**–**21** were tested for their inhibitory effects on NO production in LPS-stimulated murine BV-2 microglial cells. All isolates were non-cytotoxic to BV-2 cells at the tested concentrations (Figure 6A), and their NO inhibition ratios ranged from 10.0% to 90.0% (Figure 6B). As shown in Table 3, compounds **1**, **2**, **4**, **8**, **12** and **17** exerted anti-neuroinflammatory activities with IC_50_ values ranging from 5.0 to 20.7 μM, among which duclauxamide C (**8**) displayed the most significant activity with an IC_50_ value of 5.0 μM, presenting a comparable effect to the natural product quercetin.

**Figure 7:**
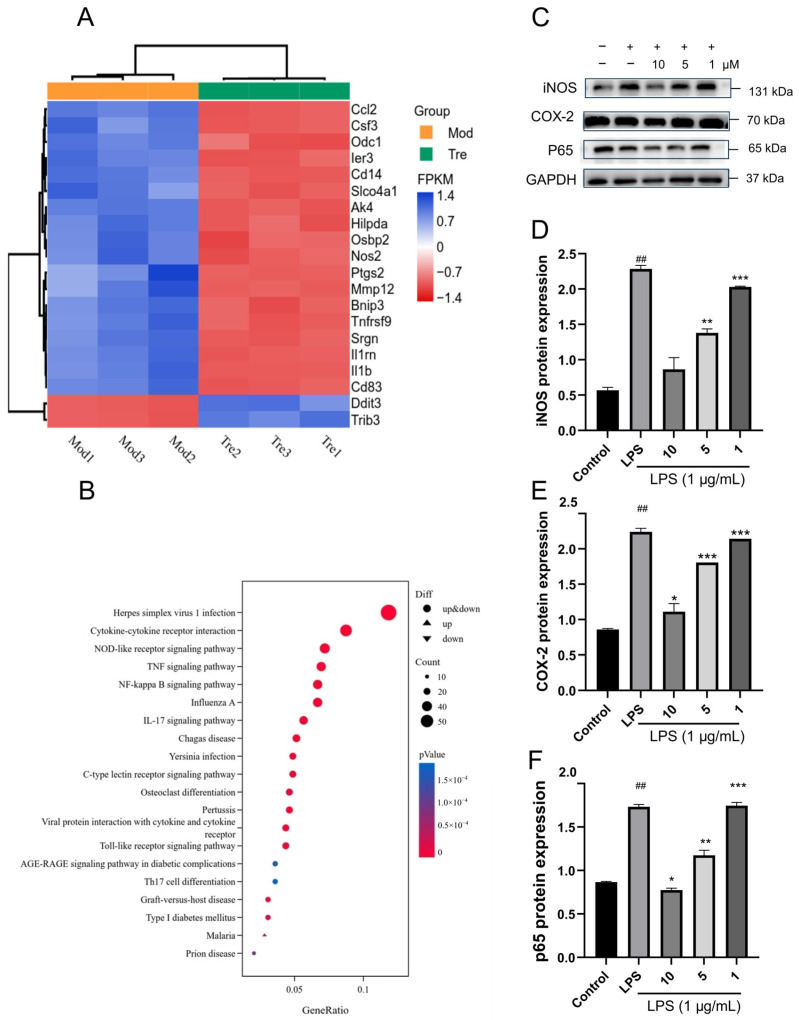
(**A**) The enriched pathways of 20 **8**-associated DEGs in neuroinflammation. (**B**) 20 **8**-associated DEGs in inflammation by GO and KEGG enrichment analysis. (**C**–**F**) The expression levels of iNOS, COX-2, and p65 were detected by Western blotting after individual treatment with **8** for 24 h in LPS-stimulated BV-2 cells. All values are expressed as mean ± SEM from three independent experiments. Compared with the control group, ^##^ *p* < 0.01; Compared with the model group (RSL3), * *p* < 0.05, ** *p* < 0.01, and *** *p* < 0.001.

To further elucidate the mechanism by which **8** inhibits neuroinflammation, transcriptome sequencing (RNA-seq) in BV-2 cells was employed. The total mRNA from BV-2 cells in the model group (LPS) and the drug group (LPS + **8**) was sequenced. After screening with a threshold of |log (FC)| > 0.4, compared with the model group, 87 genes were upregulated and 61 genes were downregulated in the drug group. The top 20 significantly differentially expressed genes (DEGs) shared between the two groups were screened out, mainly including Ccl2, Csf3, Ier3, Cd14, Slco4a1, Ak4, Hilpda, Osbp2, Nos2, Ptgs2, Mmp12, Tnfrsf9, Il1rn, Il1b, and Cd83, all of which were downregulated in the drug group, and upregulated in the model group. In contrast, Trib3 and Ddit3 were upregulated in the drug group and downregulated in the model group (Figure 7A). Bubble plots of Kyoto Encyclopedia of Genes and Genomes (KEGG) pathway analysis and Gene Ontology (GO) functional enrichment analysis indicated that among the top 20 enriched pathways, the majority were associated with the NF-*κ*B signaling pathway (Figure 7B). Collectively, these findings suggest that compound **8** might reverse LPS-induced neuroinflammation by suppressing the NF-*κ*B signaling pathway.

In order to demonstrate the transcriptome sequencing results, Western blot analysis was applied, and the expression levels of iNOS, COX-2, and p65 were remarkably increased in the model group (*p* < 0.01) (Figure 7C–F), indicating that BV-2 cells produced an inflammatory response under LPS induction and that the NF-*κ*B signaling pathway was abnormally activated. This was consistent with previous results showing that the differential genes Nos2 and Ptgs2 (encoding iNOS and COX-2 proteins, respectively) were upregulated in the model group. In contrast, the intervention with **8** could significantly decrease the expression of the three proteins (*p* < 0.01) in a dose-dependent manner (Figure 7C–F), which was consistent with the characteristics of downregulated Nos2 and Ptgs2 gene expression and inhibitory regulation related to the NF-*κ*B signaling pathway in the drug group.

**Figure 8:**
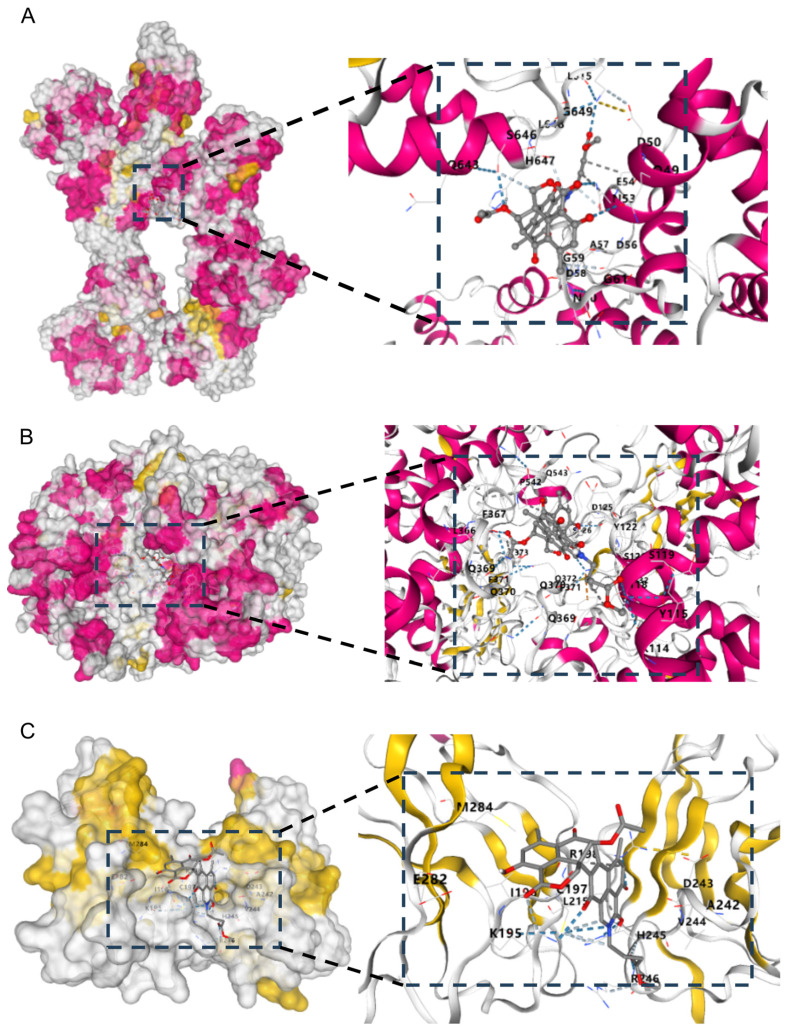
(**A**) Binding mode of **8** and iNOS in molecular docking analysis. (**B**) Binding mode of **8** and COX-2 in molecular docking analysis. (**C**) Binding mode of **8** and p65 in molecular docking analysis.

Molecular docking studies of **8** with iNOS, COX-2, and p65 were further performed to elucidate the direct binding modes. As illustrated, compound **8** formed hydrogen bonds with residues Q49, N53, and S646 in iNOS (Figure 8A), while engaging in hydrophobic interactions with residues L615, L648, and A57, with a binding energy of −10.4 kcal/mol. Similarly, compound **8** achieved binding to COX-2 (Figure 8B) primarily through hydrogen bonds formed with residues Q543 and T118, accompanied by hydrophobic interactions with residues L366, I124, F367, F371, and P542, and its binding energy was determined to be −10.2 kcal/mol. For binding with p65 (Figure 8C), compound **8** established hydrogen bonds with residues K195, R198, and R246, and underwent hydrophobic interactions with residues I196, L215, V244, A242, and M284, with a corresponding binding energy of −10.2 kcal/mol.

## 3. Materials and Methods

### 3.1. General Experimental Procedures

Optical rotations (ORs) were measured using an Autopol III automatic polarimeter (Rudolph Research Analytical, Hackettstown, NJ, USA). UV data and ECD spectra were obtained using a Chirascan spectrometer (Applied Photophysics, Ltd., Leatherhead, Surrey, UK). IR spectra were obtained using a Bruker TENSOR27 spectrophotometer (Bruker Optics, Karlsruhe, Germany) with KBr pellets. One-dimensional and 2D NMR (COSY, HSQC, HMBC, and NOESY) spectra were acquired using Bruker Avance-400 and 800 spectrometers (Beijing Oubeire Co., Ltd. Beijing, China). High-resolution electrospray ionization mass spectrometry (HR-ESI-MS) spectra were acquired using a Triple TOF 5600+ (AB SCIEX, Framingham, MA, USA). Semipreparative reversed-phase high-performance liquid chromatography (RP-HPLC) and semipreparative normal-phase high-performance liquid chromatography (NP-HPLC) were performed using an Agilent 1100 (Agilent Technologies, Inc., Santa Clara, CA, USA) liquid chromatography system equipped with an Agilent C_18_ column (EclipseXDB-C_18_, 5 μm, 9.4 × 250 mm, Agilent Technologies, Inc., Santa Clara, CA, USA) and a Welch SiO_2_ column (Ultimate, 5 μm, 10 × 250 mm, Welch Materials, Inc., Shanghai, China). Column chromatography (CC) was performed using silica gel (300–400 mesh, Qingdao Marine Chemical Inc., Qingdao, Shandong, China), C_18_ reversed-phase (RP) silica (ODS-AQ-HG GEL, AQG 12S50, YMC, Co., Ltd., Kyoto, Kyoto Prefecture, Japan), and Sephadex LH-20 gel (GE Healthcare, Inc., Chicago, IL, USA). The experimental results were monitored by thin-layer chromatography (TLC) using silica gel plates (type H) (Qingdao Dingkang Silicone Co., Ltd., Qingdao, Shandong, China). All other chemicals used in this study were of analytical grade.

### 3.2. Fungal Material and Fermentation

A total of 21 fungal strains were isolated from soil samples collected at Taibai Mountain (GPS coordinates: 33.9550° N, 107.7633° E), Shaanxi Province, China, in May 2017. Among them, the fungal strain *Talaromyces stipitatus* was obtained and identified based on ITS gene sequence analysis (Figures S62–S64). This strain was preserved at the Shaanxi Provincial Key Laboratory of Natural Product Chemical Biology, College of Chemistry and Pharmacy, Northwest A & F University, Shaanxi, China. The fungal strain *Talaromyces* sp. was cultured on potato dextrose agar (PDA) plates at 28 °C for 5 days. The colonies grown on the plates were cut into squares (0.5 × 0.5 × 0.5 cm^3^) and cultivated in potato dextrose (PDB) liquid medium in a shaking incubator at 120 rpm and 28 °C for 5 days. Subsequently, a 20 mL seed culture was transferred into a 1 L Erlenmeyer flask preloaded with 200 g of rice and 200 mL of distilled water, with the total fermentation scale reaching 40 kg. After the fungi were fermented at 28 °C for 30 days, the fermented rice medium was subjected to methanol extraction three times. The combined methanol extract was filtered under vacuum and then concentrated to dryness under reduced pressure to afford the crude extract. The crude extract was redissolved and extracted with ethyl acetate/water (*v*/*v*, 1:1) five times. Then, the organic layer was concentrated under reduced pressure to yield the crude extract (261 g).

### 3.3. Extraction and Purification

The extract was then fractionated using silica gel CC eluted with a step gradient of PE–EtOAc (*v*/*v*, 100:0, 50:1, 20:1, 10:1, 5:1, 2:1, 1:1) and CH_2_Cl_2_–MeOH (*v*/*v*, 100:0, 100:1, 100:2, 100:4, 100:8, 100:16, 100:32, 0:100) to provide 5 fractions (Fr.1–Fr.5). Fraction 1 was chromatographed on C_18_ silica gel CC with a gradient of CH_3_OH−H_2_O (30–100%, *v*/*v*) to give four fractions (Fr.1-1–Fr.1-4). Fr.1-1 was further purified by Sephadex LH-20 gel filtration chromatography, followed by preparative reversed-phase high-performance liquid chromatography (RP-HPLC) (CH_3_CN:H_2_O, 48:52), to afford compound **4** (t_R_ = 33 min, 28 mg). Fr.1-2 was separated by RP-HPLC (CH_3_CN:H_2_O, 54:46) to obtain compound **15** (t_R_ = 30 min, 10 mg). Fr.1-3 was purified by RP-HPLC (CH_3_OH:H_2_O, 70:30) to yield compounds **13** (t_R_ = 30 min, 17 mg) and **14** (t_R_ = 38 min, 10.5 mg). Fr.1-4 was separated by RP-HPLC (CH_3_OH:H_2_O, 85:15) to obtain compounds **20** (t_R_ = 25 min, 17.1 mg) and **21** (t_R_ = 35 min, 13.3 mg). Fraction 2 was chromatographed on C_18_ silica gel CC with a gradient of CH_3_OH−H_2_O (30–100%, *v*/*v*) to give five fractions (Fr.2-1–Fr.2-5). Fr.2-1 was isolated by Sephadex LH-20 and then purified by semipreparative HPLC with CH_3_OH:H_2_O (58:42), to give compound **17** (t_R_ = 27 min, 10.3 mg). Fr.2-2 was separated by RP-HPLC (CH_3_OH:H_2_O, 70:30) to obtain compound **19** (t_R_ = 20 min, 3.6 mg). Fr. 2-3 was subjected to chromatography over Sephadex LH-20 (MeOH) and further purified by RP-HPLC (CH_3_OH:H_2_O, 70:30) to yield compound **16** (t_R_ = 27 min, 34.8 mg). Fr.2-4 was separated by RP-HPLC (CH_3_CN:H_2_O, 65:35) to obtain compound **6** (t_R_ = 30 min, 3 mg). Fr.2-5 was isolated on Sephadex LH-20 (MeOH) and subsequently purified by semipreparative RP-HPLC (CH_3_OH:H_2_O, 67:33) to obtain compound **9** (t_R_ = 41 min, 12.7 mg). Fraction 3 was chromatographed on C_18_ silica gel CC with a gradient of CH_3_OH−H_2_O (30–100%, *v*/*v*) to give seven fractions (Fr.3-1–Fr.3-7). Fr.3-1 was separated by RP-HPLC (CH_3_OH:H_2_O, 62:38) to obtain compound **5** (t_R_ = 31 min, 9 mg). Fr. 3-2 was subjected to chromatography over Sephadex LH-20 (MeOH) and further purified by RP-HPLC (CH_3_OH:H_2_O, 65:35) to yield compound **18** (t_R_ = 37 min, 13.7 mg). Fr.3-3 was separated by RP-HPLC (CH_3_CN:H_2_O, 57:43) to obtain compounds **1** (t_R_ = 35 min, 8.8 mg) and **2** (t_R_ = 42 min, 7.6 mg). Fr.3-4 was purified by RP-HPLC (CH_3_OH:H_2_O, 81:19) to yield compound **8** (t_R_ = 30 min, 7 mg). Fr.3-5 was separated by RP-HPLC (CH_3_CN:H_2_O, 60:40) to obtain compound **10** (t_R_ = 33 min, 9.8 mg). Fr.3-6 was purified by RP-HPLC (CH_3_CN:H_2_O, 55:45) to yield compounds **7** (t_R_ = 33 min, 11 mg) and **3** (t_R_ = 40 min, 22 mg). Fr.3-7 was separated by RP-HPLC (CH_3_OH:H_2_O, 73:27) to obtain compound **12** (t_R_ = 35 min, 14 mg). Fraction 4 was chromatographed on C_18_ silica gel CC with a gradient of CH_3_OH−H_2_O (30–100%, *v*/*v*) to give Fr.4-1. Fr.4-1 was subjected to Sephadex LH-20 (MeOH) and then purified using semipreparative RP-HPLC (CH_3_CN:H_2_O, 70:30) to afford compound **11** (t_R_ = 44 min, 10 mg).

### 3.4. Structural Elucidation

Talarostipin A (**1**). Orange solid; [α]25 D + 41.7 (c 0.041, MeOH); UV (MeOH) λ_max_ (log ε) 232 (4.90) nm; IR (KBr) V_max_ 3417, 2789, 2703, 1618, 1354, 761 cm^−1^; ECD (1.03 × 10^−3^ mM, MeOH) λmax (Δε) 370 (+4.90), 340 (+3.39), 292 (−9.93), 244 (+30.28), 211 (−24.07) nm; HRESIMS [M + H]^+^ *m*/*z* 761.2542 (calcd for C_39_H_41_N_2_O_14_^+^, 761.2558); ^1^H and ^13^C NMR data, see Table 1.

Talarostipin B (**2**). Orange solid; [α]25 D + 38.7 (c 0.038, MeOH); UV (MeOH) λ_max_ (log ε) 232 (4.27) nm; IR(KBr) V_max_ 3432, 2784, 2699, 1626,1360, 764 cm^−1^; ECD (0.99 × 10^−3^ mM, MeOH) λmax (Δε) 371 (+3.73), 338 (+2.60), 292 (−11.06), 244 (+28.30), 211 (−26.23) nm; HRESIMS [M + H]^+^ *m*/*z* 775.2704 (calcd for C_40_H_43_N_2_O_14_^+^, 775.2714); ^1^H and ^13^C NMR data, see Table 2.

Talarostipin C (**3**). Orange solid; [α]25 D–185.2 (c −0.185, MeOH); UV (MeOH) λ_max_ (log ε) 200 (8.91) nm; IR(KBr) V_max_ 3407, 2794, 2699, 1618, 1357, 762 cm^−1^; ECD (1.00 × 10^−3^ mM, MeOH) λmax (Δε) 323 (−3.58), 288 (+1.95), 251 (−2.78), 229 (−16.05), 200 (+15.82) nm; HRESIMS [M + H]^+^ *m*/*z* 572.1548 (calcd for C_31_H_26_NO_10_^+^, 572.1557); ^1^H and ^13^C NMR data, see Table 2.

Talarostipin D (**4**). Yellow solid; [α]25 D–3.8 (c −0.038, MeOH); UV (MeOH) λ_max_ (log ε) 200 (11.0) nm; IR(KBr) V_max_ 2794, 2689, 2362, 1628, 1345, 777 cm^−1^; ECD (0.97 × 10^−3^ mM, MeOH) λmax (Δε) 374 (+5.85), 335 (+3.68), 293 (−7.85), 243 (+33.54), 211 (−31.45) nm; HRESIMS [M + H]^+^ *m*/*z* 590.1652 (calcd for C_31_H_28_NO_11_^+^, 590.1662); ^1^H and ^13^C NMR data, see Table 2.

Talarostipin E (**5**). Yellow solid; [α]25 D–7.4 (c −0.007, MeOH); UV (MeOH) λ_max_ (log ε) 200 (12.0) nm; IR(KBr) V_max_ 3417, 2794, 2703, 1621, 1347, 772 cm^−1^; ECD (1.03 × 10^−3^ mM, MeOH) λmax (Δε) 399 (−5.89), 330 (+8.10), 284 (+8.82), 242 (+31.96), 217 (−37.67), 200 (+4.40) nm; HRESIMS [M + H]^+^ *m*/*z* 549.1030 (calcd for C_28_H_21_NO_12_^+^, 549.1033); ^1^H and ^13^C NMR data, see Table 2.

The NMR data of known compounds are available in the Supplementary Materials.

### 3.5. Electronic Circular Dichroism (ECD) Spectroscopy

An accurately weighed sample was dissolved in spectroscopic-grade methanol to prepare a test solution with a concentration of approximately 1.0 mg/mL. The measurement was performed at room temperature using a J-1500 circular dichroism spectrophotometer equipped with a quartz cell with a 0.1 cm path length. The scanning parameters were set as follows: wavelength range 190–400 nm, scanning speed 100 nm/min, bandwidth 1.0 nm, and response time 1 s. Each sample was scanned three times consecutively. The obtained raw spectra were smoothed, and the solvent background was subtracted. The final ECD spectrum was generated by averaging the three individual scans. Results are presented as a plot of ellipticity (mdeg) versus wavelength (nm). In the corresponding ECD spectrum, the compound exhibited characteristic Cotton effects: a positive band at 210 nm and a negative band at 245 nm.

### 3.6. Cell Culture

Murine BV-2 microglia cell line was obtained from the Cell Resource Centre of Peking Union Medical College (Beijing, China). The cells were cultured in Dulbecco’s modified Eagle’s medium (DMEM, Gibco, Grand Island, NY, USA) containing 10% fetal bovine serum (FBS, Gibco, Grand Island, NY, USA) and 1% penicillin/streptomycin (Beyotime Biotechnology, Shanghai, China) in a humidified incubator at 37 °C with 5% CO_2_.

### 3.7. Cytotoxicity Assay

Cell viability was measured using a Cell Counting Kit-8 (CCK8, Beyotime Biotechnology, Shanghai, China). Briefly, 2 × 10^4^ BV-2 cells were cultured in each well of a 96-well cell culture plate in 100 μL medium for 24 h. After attachment for 24 h at 37 °C, 10 μL of CCK-8 solution was added to each well and incubated for 2 h. The absorbance was detected at 450 nm with a microplate reader (Synergy HTX, Bio Tek Instruments Inc., Winooski, VT, USA).

### 3.8. Anti-Neuroinflammation Assay

The inhibitory activity of the compounds on NO release was indirectly determined using Griess reagents. Briefly, BV-2 cells were seeded at a density of 2.5 × 10^4^ cells/well in 96-well plates and then treated with LPS (1 μg/mL) in the presence or absence of the tested compounds for 24 h. After that, the culture media was mixed with an equal volume of Griess reagents I and II. The absorbance was measured at 540 nm.

### 3.9. KEGG and GO Enrichment Analysis

KEGG and GO functional enrichment analyses were performed using matascape6 for the related targets of compound **8** and ulcerative colitis. In KEGG enrichment analysis, −lg(*P*) was selected as the screening condition, and the top 20 pathways twenty significantly dysregulated genes in the results were selected for visualization. GO enrichment analysis also used −lg(*P*) as the significant enrichment screening condition, and the top 20 signaling pathways were selected to generate bubble maps for visualization.

### 3.10. Western Blotting

To verify the regulatory role of differentially expressed genes and the activation status of the NF-*κ*B signaling pathway, Western blot analysis was performed in this experiment to detect the expression levels of three key proteins (iNOS, COX-2, and P65) in cells of each group. Three technical replicates were strictly set up for the experiment, and the gray values were quantitatively analyzed using ImageJ software (Image Lab 5.2). Glyceraldehyde-3-phosphate dehydrogenase (GAPDH) was used as an internal reference protein to correct experimental errors, ensuring the reliability and reproducibility of the detection results.

### 3.11. Molecular Docking

AutoDock 4.2 Vina software and AutoDock Tools (ADT 1.5.6) were utilized to explore the binding patterns and interaction sites between small-molecule compounds and target proteins. The 3D crystal structures of transcription factor iNOS (PDB ID: 3HR4), COX-2 (PDB ID: 8ET0) and p65 (PDB ID: 6GGR) were downloaded from the RCSB Protein Data Bank (http://www.rcsb.org, accessed on 18 January 2026). The standard three-dimensional structure of the compound (in PDB format) was constructed using the “SKETCH” function embedded in SYBYL-X software (version 2.1). The interactions between compound 8 and the target proteins were simulated in a cubic grid box with dimensions of 4 Å (x, y, z) and a grid spacing of 0.375 Å, while other parameters were set to default values. For the ligand molecule, torsion and rotation were allowed during the entire docking process to obtain the optimal binding free energy, thereby determining the final complex structure and corresponding binding sites.

## 4. Conclusions

Five new duclauxin analogues, talarostipins A–E (**1**–**5**), along with sixteen known polycyclic oligophenalenone dimers (**6**–**21**), were isolated and identified from the soil-derived fungus *Talaromyces stipitatus*. In the bioassay, duclauxamide (**8**) demonstrated noticeable anti-neuroinflammatory effects in LPS-stimulated BV-2 microglial cells. Quantitative Western blot analysis revealed that **8** significantly and dose-dependently suppressed the LPS-induced protein expression of iNOS and COX-2, two key mediators of the inflammatory response. Furthermore, treatment with **8** markedly inhibited the p65 subunit of NF-*κ*B, a predominant transcription factor governing neuroinflammatory signaling. In the present study, compound **8** exhibited anti-neuroinflammatory potency comparable to that of quercetin. Notably, compound **8** possesses a unique heptacyclic oligophenalenone dimer scaffold that is rarely encountered in natural products, thereby offering a novel structural template for further optimization to enhance its potency and blood–brain barrier permeability. Collectively, these findings indicate that **8** may be a potential lead molecule for the treatment of Alzheimer’s disease by targeting neuroinflammation.

## Figures and Tables

**Table 1:** ^1^H NMR and ^13^C NMR data for compounds **1**, **2**, and **4**.

NO.	1 ^a^		2 ^a^		4 ^b^	
	*δ*_C_	*δ*_H_ (*J* in Hz)	*δ*_C_	*δ*_H_ (*J* in Hz)	*δ*_C_	*δ*_H_ (*J* in Hz)
1	135.92	8.02, s, 1H	136.28	8.14, s, 1H	135.32	7.86, s, 1H
3	165.29		165.77		164.10	
3a	106.94		106.81		107.03	
3b	134.67		134.74		137.40	
4	161.62		161.61		161.26	
5	118.45	6.76, s, 1H	118.38	6.76, s, 1H	117.08	6.74, s, 1H
6	149.56		149.69		148.47	
6a	116.76		116.80		116.73	
7	88.63		88.56		88.48	
8	63.99	4.01, s, 1H	63.97	4.00, s, 1H	63.88	3.98, s, 1H
9	194.41		193.17		193.16	
9a	111.51		111.32		110.90	
10	21.94	2.70, s, 3H	22.05	2.71, s, 3H	21.08	2.70, s, 3H
1′	71.51	5.10, d (12.3), 1H	71.46	5.08, d (12.7), 1H 4.75, d, 1H	71.29	5.21, d (12.1) 5.10, d (12.1)
		4.73, d (12.3), 1H				
3′	167.42				165.35	
3′a	104.90		167.59		105.21	
3′b	142.97		104.95		143.49	
4′	164.73		143.05		167.49	
5′	121.24	6.50, s, 1H	164.68		119.97	6.53, s, 1H
6′	151.65		120.97	6.50, s, 1H	150.69	
6′a	121.29		151.51		121.43	
7′	191.23		121.25		191.32	
8′	67.98	4.14, s, 1H	191.27		67.86	4.14, s, 1H
9′	78.82	5.20, s, 1H	67.98	4.14, s, 1H	78.95	5.18, s, 1H
9′a	51.53		78.85	5.20, s, 1H	51.21	
10′	22.59	2.05, s, 3H	51.53		21.25	1.99, s, 3H
1″	60.11	4.07, m, 1H	67.06	4.51, m, 2H	59.14	4.12, s, 1H 3.87, overlap
		4.21, m, 1H	67.06	4.51, m, 1H	50.80	3.78, overlap
2″	61.57	5.31, t, 1H	62.70	5.23, t, 1H		
NH		6.71 1H		6.76, 1H		
4″	51.62	4.66, m, 1H	51.60	4.64, m, 1H		
5″	174.15		173.95			
6″	52.88	3.80, s, 3H	52.83	3.79, s, 3H		
7″	41.04	1.62, m, 2H	40.91	1.61, m, 2H		
8″	25.06	1.66, m, 1H	25.15	1.69, m, 1H		
9″	22.89	0.95, overlap, 3H	22.84	0.95, m, 3H		
10″	22.05	0.95, overlap, 3H	22.08	0.95, m, 3H		
11″	167.03		166.70			
12″			19.85	1.22, d (4.0), 3H		
9′-OCOCH3_3_	169.79		169.83		169.47	
9′-OCOCH_3_	21.11	2.23, s, 3H	21.11	2.23, s, 3H	20.01	2.21, s, 3H
7-OCH_3_	51.73	2.96, s, 3H	51.79	2.95, s, 3H	50.81	2.98, s, 3H
4-OH		12.07, s		12.06, s		12.56, s
4′-OH		11.58, s		11.58, s		

^a^ Recorded in CDCl_3_. ^b^ Recorded in Acetone-*d_6_*.

**Table 2:** ^1^H NMR and ^13^C NMR data for compounds **3** and **5** (Acetone-*d_6_*).

NO.	3		5	
	*δ*_C_	*δ*_H_ (*J* in Hz)	*δ*_C_	*δ*_H_ (*J* in Hz)
1	143.86	8.52, s, 1H	80.33	5.44, dd, 1H
3	166.04		167.85	
3a	109.00		108.74	
3b	135.73		143.96	
4	164.84		162.86	
5	118.35	6.78, s, 1H	122.19	6.97, s, 1H
6	149.53		148.24	
6a	115.71		118.49	
7	152.83		156.57	
8	140.65		137.83	
9	178.36		192.92	
9a	115.01		67.83	
10	25.13	2.99, s, 3H	24.68	2.95, s, 3H
1′	70.46	5.09, overlap, 2H	70.18	5.06, d (12.3), 2H 4.86, d (12.3)
3′	169.13		168.93	
3′a	105.29		105.18	
3′b	148.68		148.59	
4′	164.75		164.90	
5′	120.44	6.72, s, 1H	120.54	6.78, s, 1H
6′	153.71		153.76	
6′a	118.25		119.62	
7′	192.23		191.08	
8′	67.66	4.86, overlap, 1H	68.99	4.77, s, 1H
9′	85.91	4.85, overlap, 1H	86.06	4.72, s, 1H
9′a	50.52		50.28	
10′	23.77	2.56, s, 3H	23.77	2.56, s, 3H
1″	49.97	4.20, m, 2H	170.22	
2″	25.00	2.07, t, 2H	36.57	2.05, 3H
3″	31.22	2.42, t, 2H		
4″	173.34			
5″	51.71	3.54, s, 3H		
4′-OH				
9′-OH		6.37, s		

**Table 3:** Anti-neuroinflammatory activity of compounds **1**–**21** ^a^.

Compound	Cell Viability (%, 20 μM)	IC_50_ (μM)	NO Percent Inhibition (%, 20 μM)
**1**	98.1	17 ± 1.8	57.8 ± 4.9
**2**	102.5	9.9 ± 0.3	59.2 ± 8.0
**4**	120.8	20.7 ± 2.0	51.1 ± 5.4
**8**	112.5	5.0 ± 0.7	86.5 ± 3.9
**12**	111.9	10.0 ± 1.1	57.8 ± 0.3
**17**	128.8	6.9 ± 0.5	61.8 ± 0.8
quercetin	99.0	4.8 ± 0.5	97.9 ± 2.1

^a^ Data were presented as the mean ± SD at three independent experiments.

## Data Availability

Supporting Information data include HRESIMS, UV, CD and 1D and 2D NMR spectra.

## Supplementary Materials

The following supporting information can be downloaded at: https://www.mdpi.com/article/10.3390/molecules31081308/s1, Figures S1–S6: The 1D and 2D NMR spectra of compound **1** in CDCl_3_, Figure S7: The HRESIMS spectrum of compound **1**, Figures S8 and S9: The IR and UV spectra of compound **1**, Figure S10–S15: The 1D and 2D NMR spectra of compound **2** in CDCl_3_, Figure S16: The HRESIMS spectrum of compound **2**, Figures S17 and S18: The IR and UV spectra of compound **2**, Figure S19–S24: The 1D and 2D NMR spectra of compound **3** in Acetone-*d*_6_, Figure S25: The HRESIMS spectrum of compound **3**, Figures S26 and S27: The IR and UV spectra of compound **3**, Figures S28–S33: The 1D and 2D NMR spectra of compound **4** in CDCl_3_, Figure S34: The HRESIMS spectrum of compound **4**, Figures S35 and S36: The IR and UV spectra of compound **4**, Figures S37–S42: The 1D and 2D NMR spectra of compound **5** in Acetone-*d*_6_, Figure S43: The HRESIMS spectrum of compound **5**, Figures S44 and S45: The IR and UV spectra of compound **5**, Figures S46–S61: ^1^H NMR spectrum of compounds **6**–**21**, Figure S62: The fungus ITS gene sequence, Figure S63: Plate image of the fungus, Figure S64: Microscopic images of the fungus, Table S1: NMR data of Talauxamide A (**1**) in CDCl_3_, Table S2: NMR data of Talauxamide B (**2**) in CDCl_3_, Table S3: NMR data of Talauxamide C (**3**) in Acetone-*d*_6_, Table S4: NMR data of Talauxamide D (**4**) in CDCl_3_, Table S5: NMR data of Talauxamide E (**5**) in Acetone-*d*_6_.
