# Supplementary material for: New Oligophenalenone Dimers from Talaromyces stipitatus with Potential Anti-Neuroinflammatory Activity
Source: Molecules. 2026 Apr 17;31(8):1308. doi: 10.3390/molecules31081308 (PMC13119277; doi:10.3390/molecules31081308)
Supplement: Supplementary file 1 [file molecules-31-01308-s001.zip › molecules-4241974-supplementary.pdf]

Supporting information

New oligophenalenone dimers from *Talaromyces stipitatus* with potential anti-neuroinflammation activities

Qing Liu [1], Yu Gao [1], Biaopeng Wang [1], Kehan Du [1], Shengmin Zhu [1], Yahong Yuan [1,2,*], Yu-Qi Gao [1,2,*], Tianli Yue [1,2,*]

Table of Contents

Figure S1 ^{1}H NMR spectrum of compound **1** (400 MHz, $CDCl_3$).
Figure S2 ^{13}C NMR spectrum of compound **1** (100 MHz, $CDCl_3$).
Figure S3 HSQC spectrum of compound **1** ($CDCl_3$).
Figure S4 ^{1}H–^{1}H COSY spectrum of compound **1** ($CDCl_3$).
Figure S5 HMBC spectrum of compound **1** ($CDCl_3$).
Figure S6 NOESY spectrum of compound **1** ($CDCl_3$).
Figure S7 (+)-HRESIMS of compound **1**.
Figure S8 IR spectrum (film on KBr pellet) of compound **1**.
Figure S9 UV spectrum of compound **1** (MeOH).
Figure S10 ^{1}H NMR spectrum of compound **2** (400 MHz, $CDCl_3$).
Figure S11 ^{13}C NMR spectrum of compound **2** (100 MHz, $CDCl_3$).
Figure S12 HSQC spectrum of compound **2** ($CDCl_3$).
Figure S13 ^{1}H–^{1}H COSY spectrum of compound **2** ($CDCl_3$).
Figure S14 HMBC spectrum of compound **2** ($CDCl_3$).
Figure S15 NOESY spectrum of compound **2** ($CDCl_3$).
Figure S16 (+)-HRESIMS of compound **2**.
Figure S17 IR spectrum (film on KBr pellet) of compound **2**.
Figure S18 UV spectrum of compound **2** (MeOH).
Figure S19 ^{1}H NMR spectrum of compound **3** (400 MHz, Acetone-d_6).
Figure S20 ^{13}C NMR spectrum of compound **3** (100 MHz, Acetone-d_6).
Figure S21 HSQC spectrum of compound **3** (Acetone-d_6).
Figure S22 ^{1}H–^{1}H COSY spectrum of compound **3** (Acetone-d_6).
Figure S23 HMBC spectrum of compound **3** (Acetone-d_6).
Figure S24 NOESY spectrum of compound **3** (Acetone-d_6).
Figure S25 (+)-HRESIMS of compound **3**.
Figure S26 IR spectrum (film on KBr pellet) of compound **3**.
Figure S27 UV spectrum of compound **3** (MeOH).
Figure S28 ^{1}H NMR spectrum of compound **4** (400 MHz, Acetone-d_6).
Figure S29 ^{13}C NMR spectrum of compound **4** (100 MHz, Acetone-d_6).
Figure S30 HSQC spectrum of compound **4** (Acetone-d_6).
Figure S31 ^{1}H–^{1}H COSY spectrum of compound **4** (Acetone-d_6).
Figure S32 HMBC spectrum of compound **4** (Acetone-d_6).
Figure S33 NOESY spectrum of compound **4** (Acetone-d_6).
Figure S34 (+)-HRESIMS of compound **4**.
Figure S35 IR spectrum (film on KBr pellet) of compound **4**.
Figure S36 UV spectrum of compound **4** (MeOH).
Figure S37 ^{1}H NMR spectrum of compound **5** (400 MHz, Acetone-d_6).
Figure S38 ^{13}C NMR spectrum of compound **5** (100 MHz, Acetone-d_6).
Figure S39 HSQC spectrum of compound **5** (Acetone-d_6).
Figure S40 ^{1}H–^{1}H COSY spectrum of compound **5** (Acetone-d_6).
Figure S41 HMBC spectrum of compound **5** (Acetone-d_6).
Figure S42 NOESY spectrum of compound **5** (Acetone-d_6).

Figure S43 (+)-HRESIMS of compound **5**.
Figure S44 IR spectrum (film on KBr pellet) of compound **5**.
Figure S45 UV spectrum of compound **5** (MeOH).
Figure S46 ^{1}H NMR spectrum of compound **6** (400 MHz, $CDCl_3$).
Figure S47 ^{1}H NMR spectrum of compound **7** (400 MHz, Acetone-d_6).
Figure S48 ^{1}H NMR spectrum of compound **8** (400 MHz, $CDCl_3$).
Figure S49 ^{1}H NMR spectrum of compound **9** (400 MHz, $CDCl_3$).
Figure S50 ^{1}H NMR spectrum of compound **10** (400 MHz, Acetone-d_6).
Figure S51 ^{1}H NMR spectrum of compound **11** (400 MHz, Acetone-d_6).
Figure S52 ^{1}H NMR spectrum of compound **12** (400 MHz, Acetone-d_6).
Figure S53 ^{1}H NMR spectrum of compound **13** (400 MHz, Acetone-d_6).
Figure S54 ^{1}H NMR spectrum of compound **14** (400 MHz, $CDCl_3$).
Figure S55 ^{1}H NMR spectrum of compound **15** (400 MHz, $CDCl_3$).
Figure S56 ^{1}H NMR spectrum of compound **16** (400 MHz, Acetone-d_6).
Figure S57 ^{1}H NMR spectrum of compound **17** (400 MHz, Acetone-d_6).
Figure S58 ^{1}H NMR spectrum of compound **18** (400 MHz, Acetone-d_6).
Figure S59 ^{1}H NMR spectrum of compound **19** (400 MHz, Acetone-d_6).
Figure S60 ^{1}H NMR spectrum of compound **20** (400 MHz, Acetone-d_6).
Figure S61 ^{1}H NMR spectrum of compound **21** (400 MHz, Acetone-d_6).
Figure S62 The fungus ITS gene sequence.
Figure S63 Plate image of the fungus.
Figure S64 Microscopic images of the fungus.
Table S1 NMR data of Talauxamide A (**1**) in $CDCl_3$
Table S2 NMR data of Talauxamide B (**2**) in $CDCl_3$
Table S3 NMR data of Talauxamide C (**3**) in Acetone-d_6
Table S4 NMR data of Talauxamide D (**4**) in Acetone-d_6
Table S5 NMR data of Talauxamide E (**5**) in Acetone-d_6

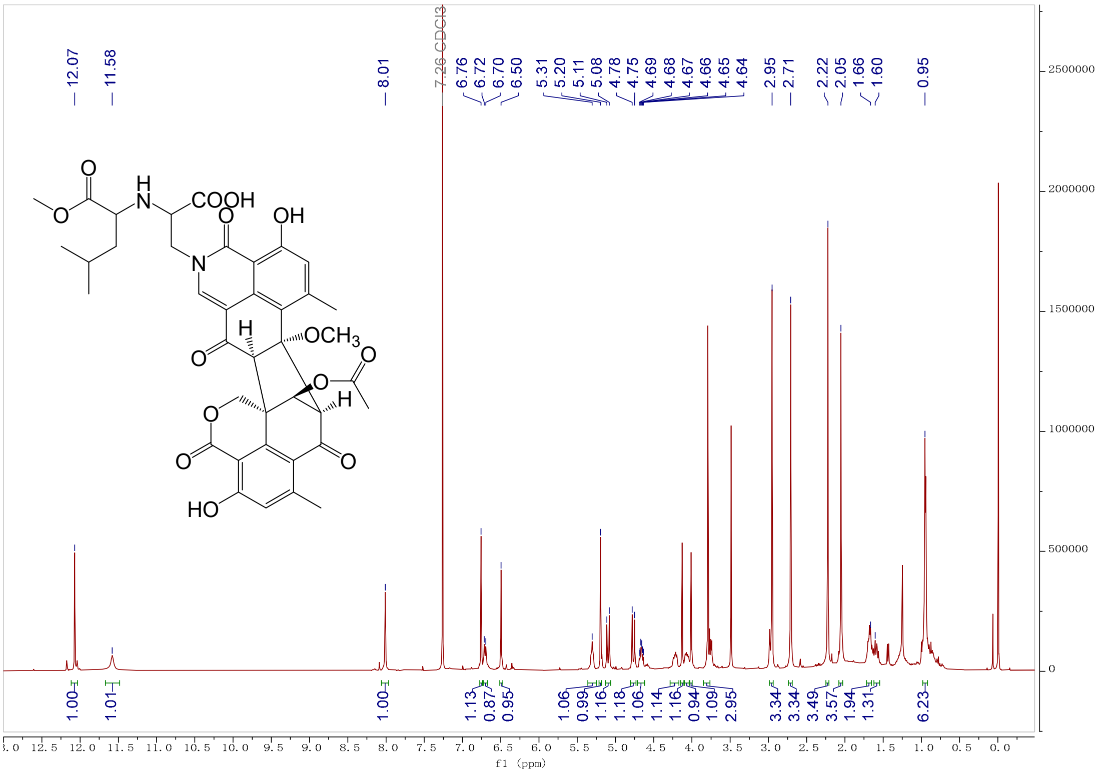

Figure S1 ^{1}H NMR spectrum of **1** (400 MHz, $CDCl_3$)

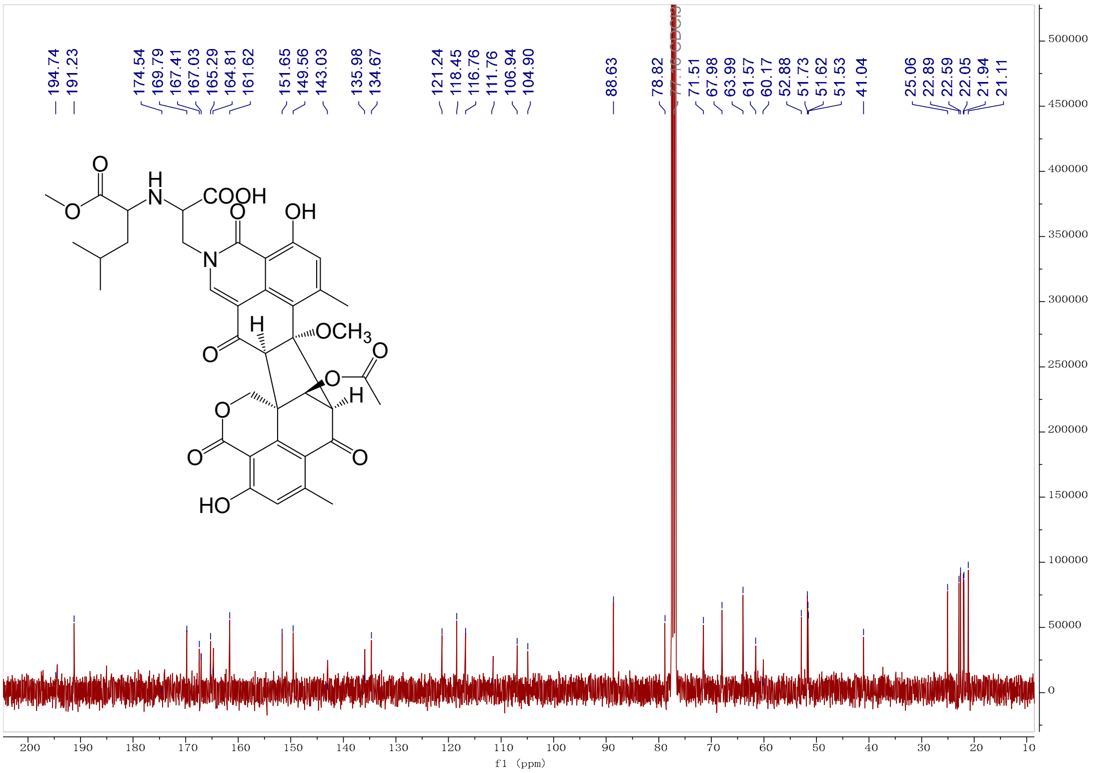

Figure S2 ^{13}CNMR spectrum of **1** (100 MHz, $CDCl_3$)

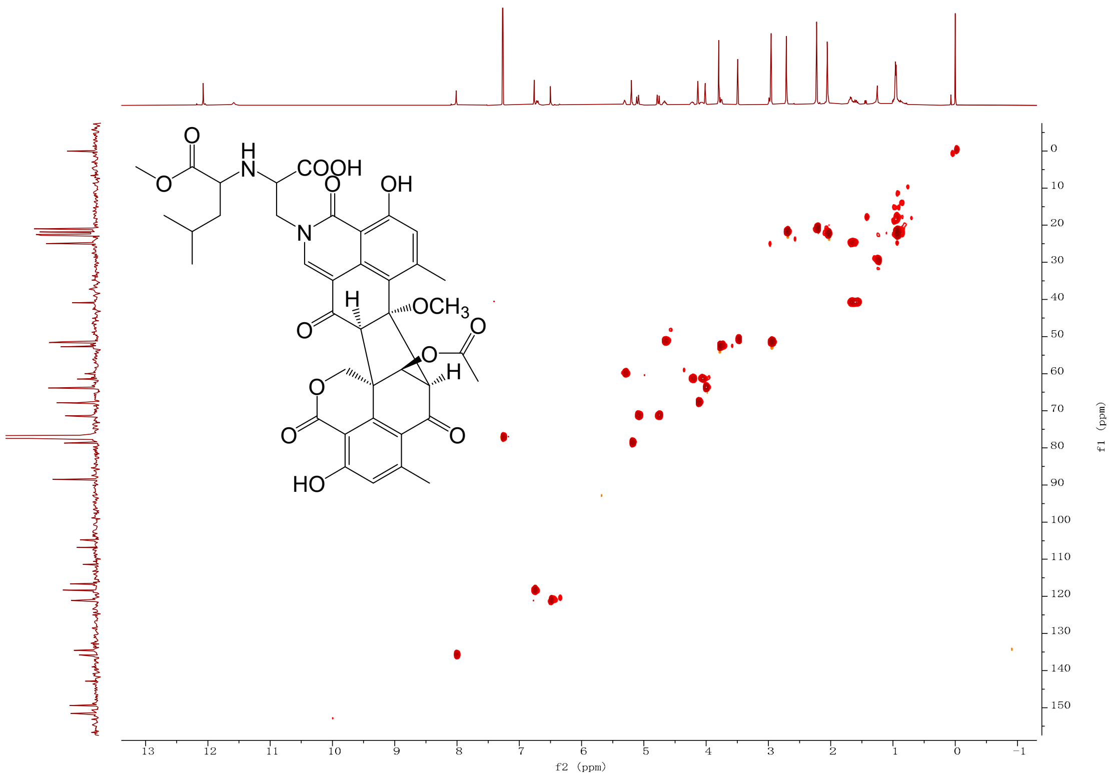

Figure S3 HSQC spectrum of **1** ($CDCl_3$)

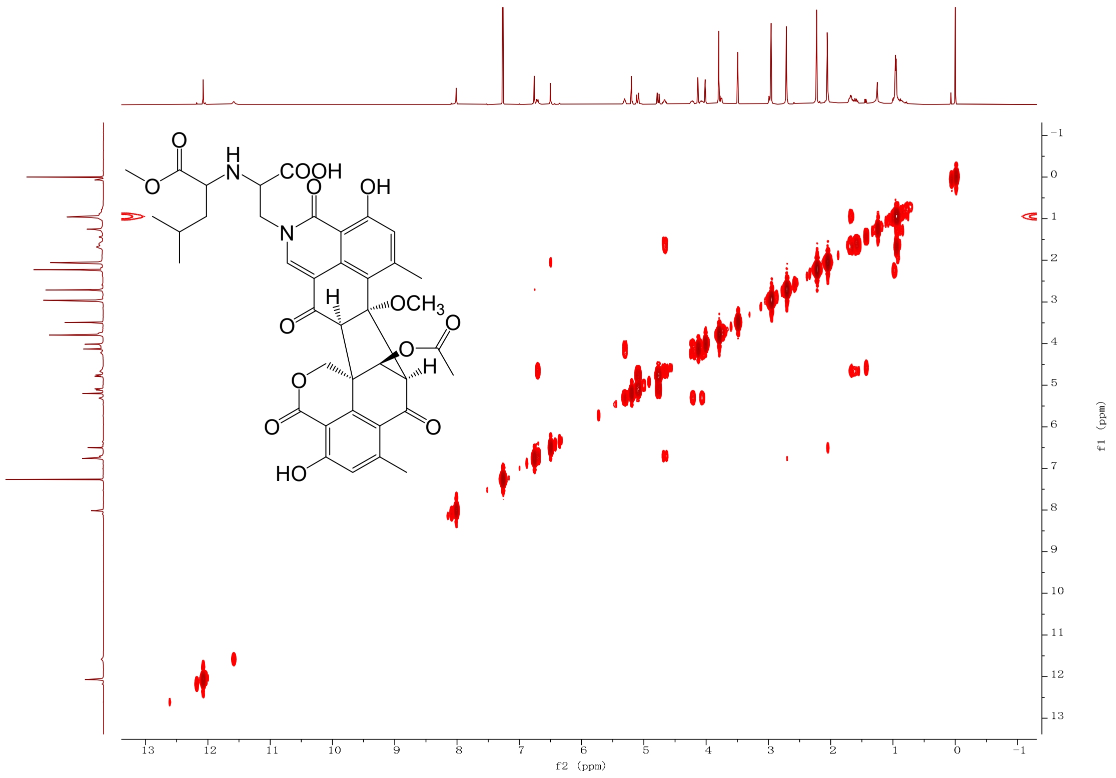

Figure S4 ^1H-^1H COSY spectrum of **1** ($CDCl_3$)

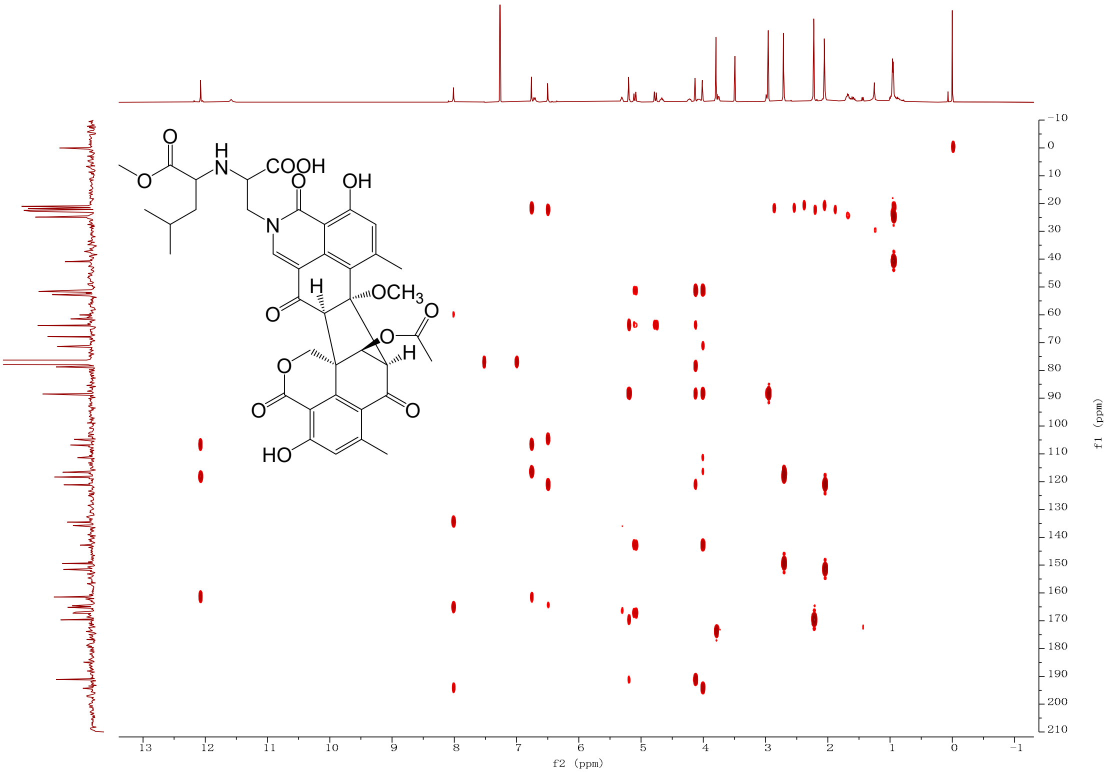

Figure S5 HMBC spectrum of **1** (CDCl₃)

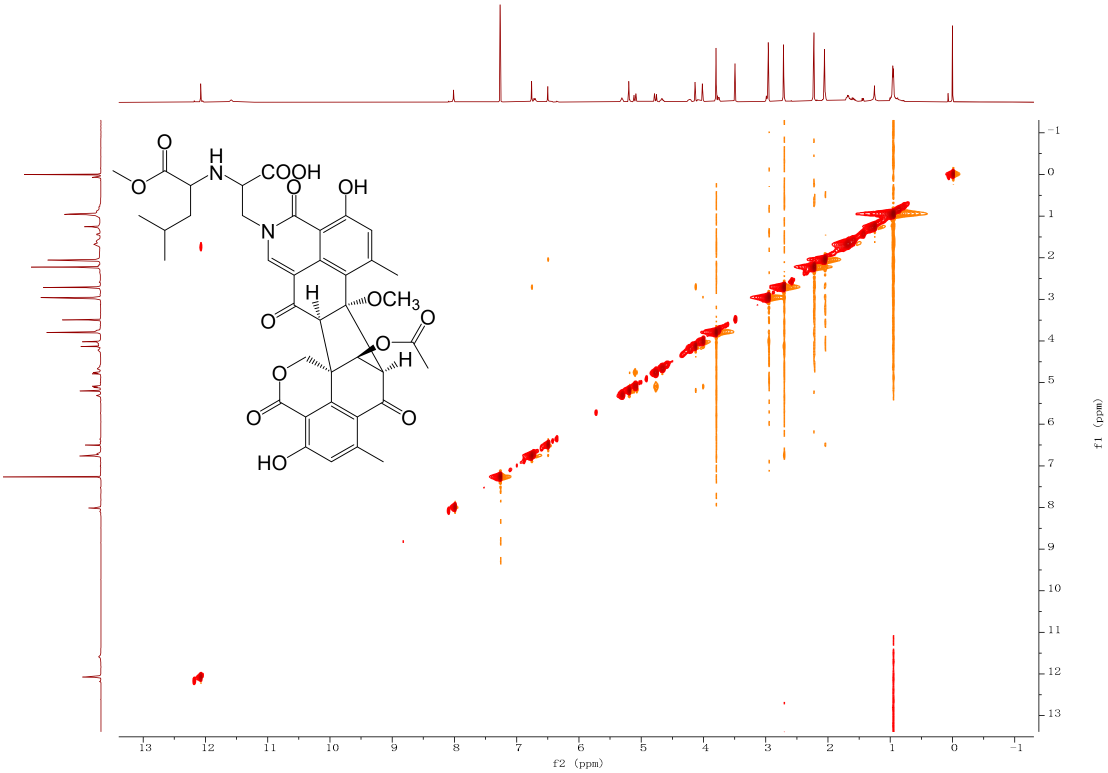

Figure S6 NOESY spectrum of **1** (CDCl₃)

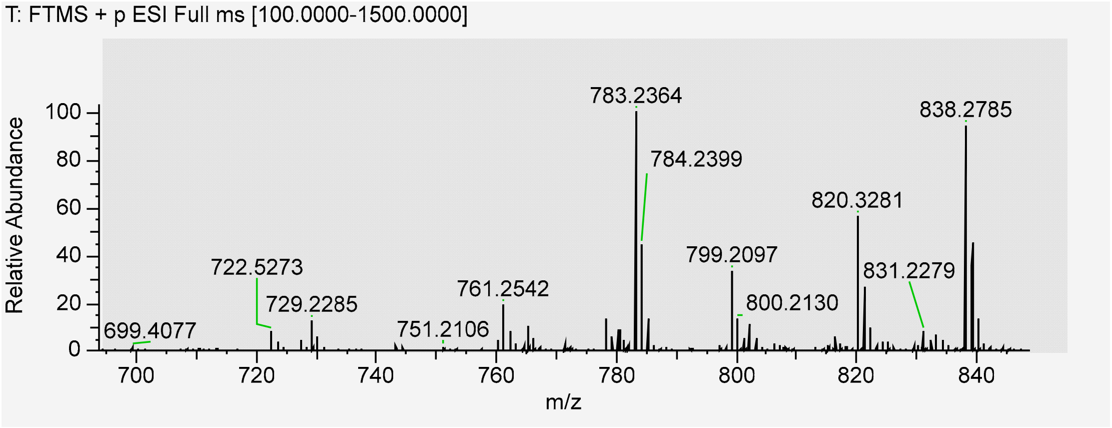

Figure S7 (+)-HRESIMS of compound **1**.

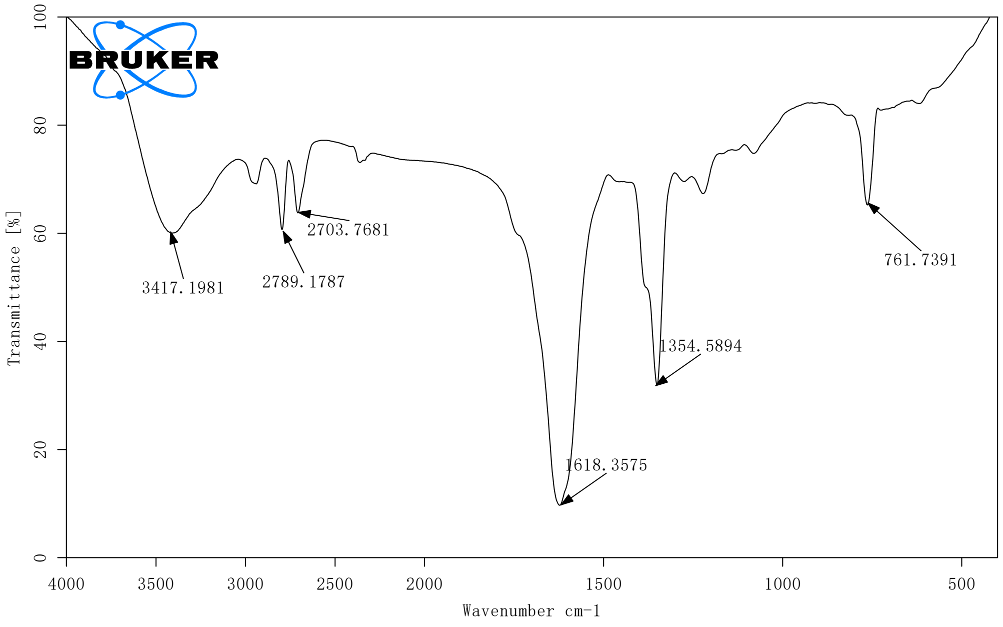

Figure S8 IR spectrum (film on KBr pellet) of compound **1**.

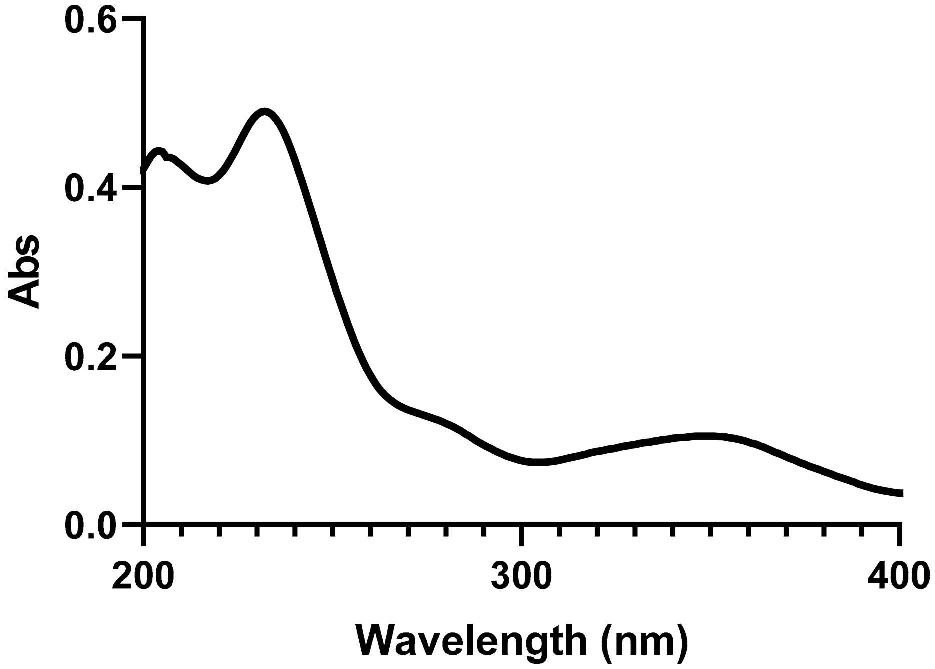

Figure S9 UV spectrum of compound **1** (MeOH).

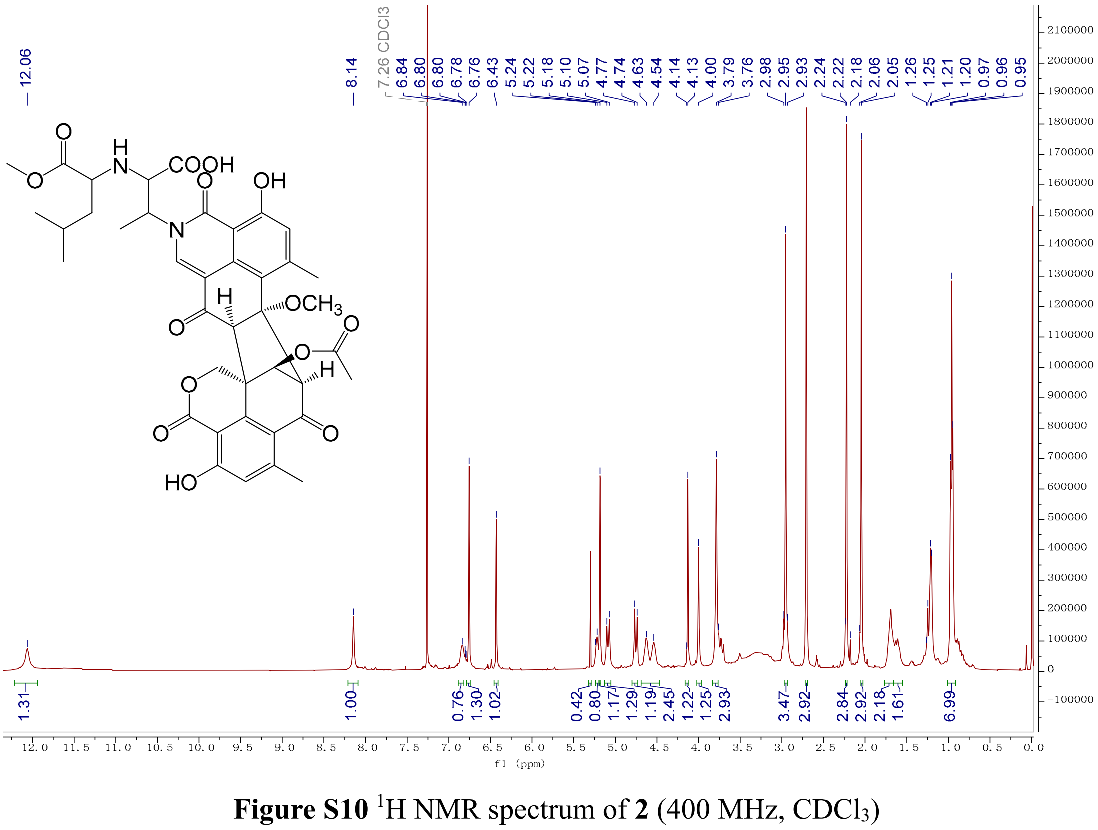

Figure S10 ^{1}H NMR spectrum of **2** (400 MHz, $CDCl_3$)

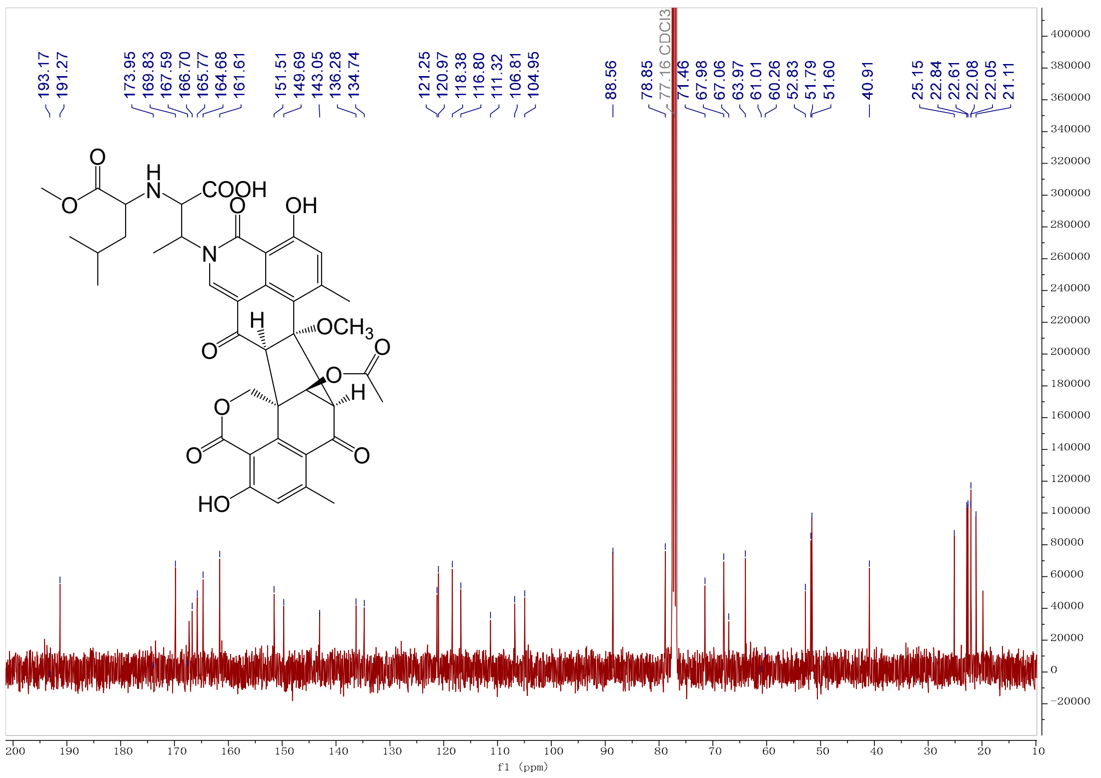

Figure S11 ^{13}CNMR spectrum of **2** (100 MHz, $CDCl_3$)

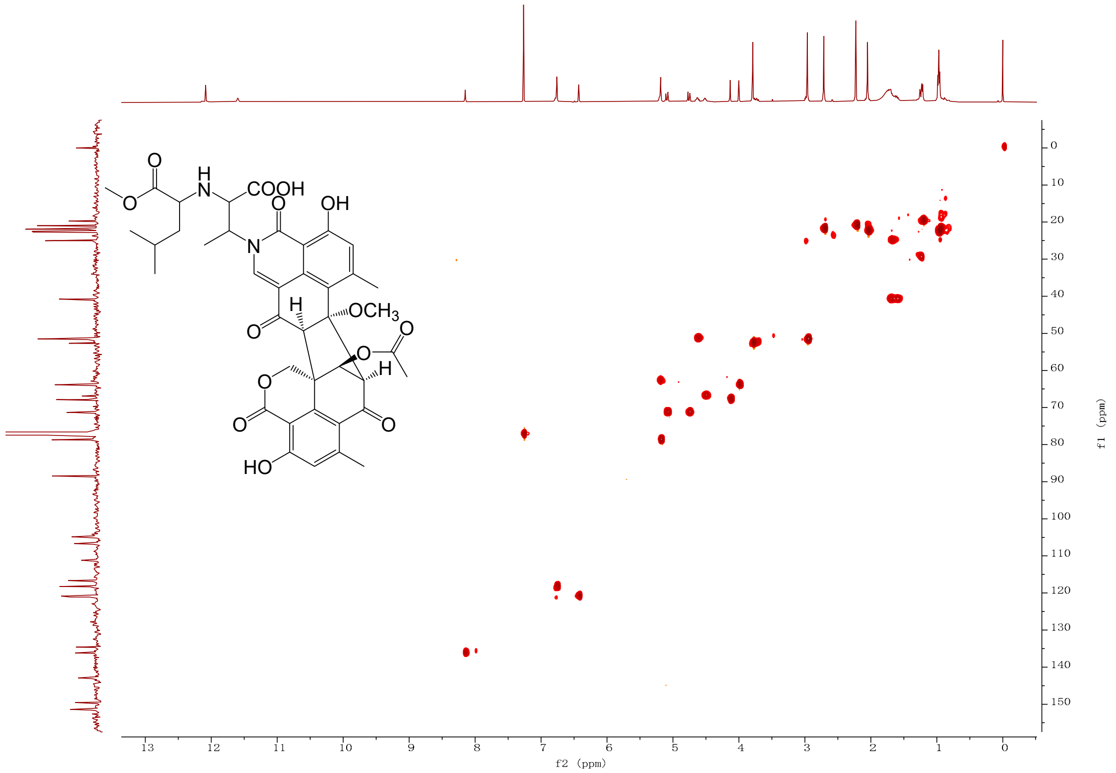

Figure S12 HSQC spectrum of **2** ($CDCl_3$)

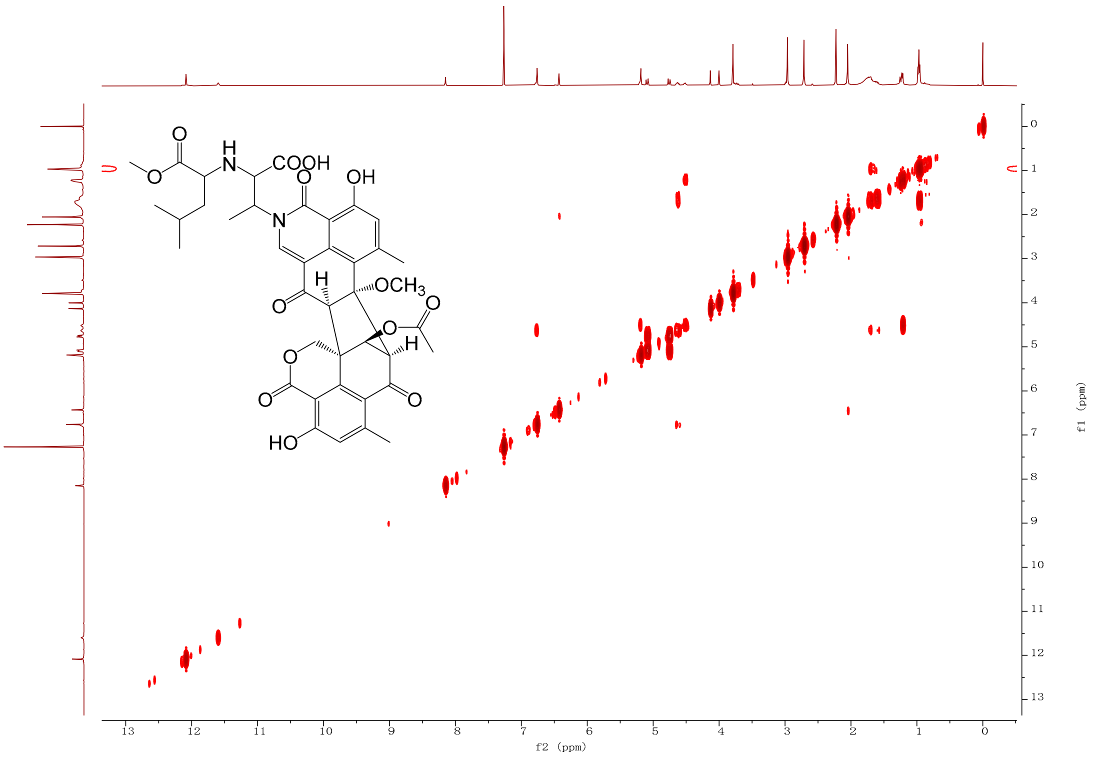

Figure S13 ^{1}H-^{1}H COSY spectrum of **2** ($CDCl_3$)

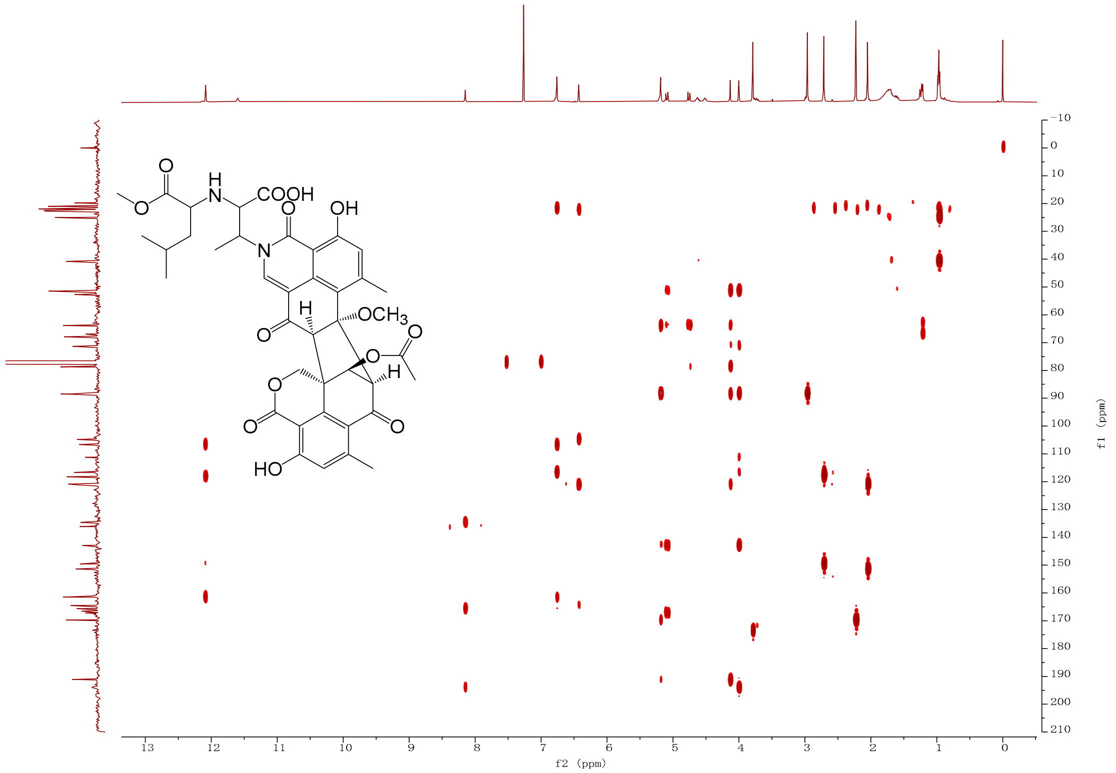

Figure S14 HMBC spectrum of **2** ($CDCl_3$)

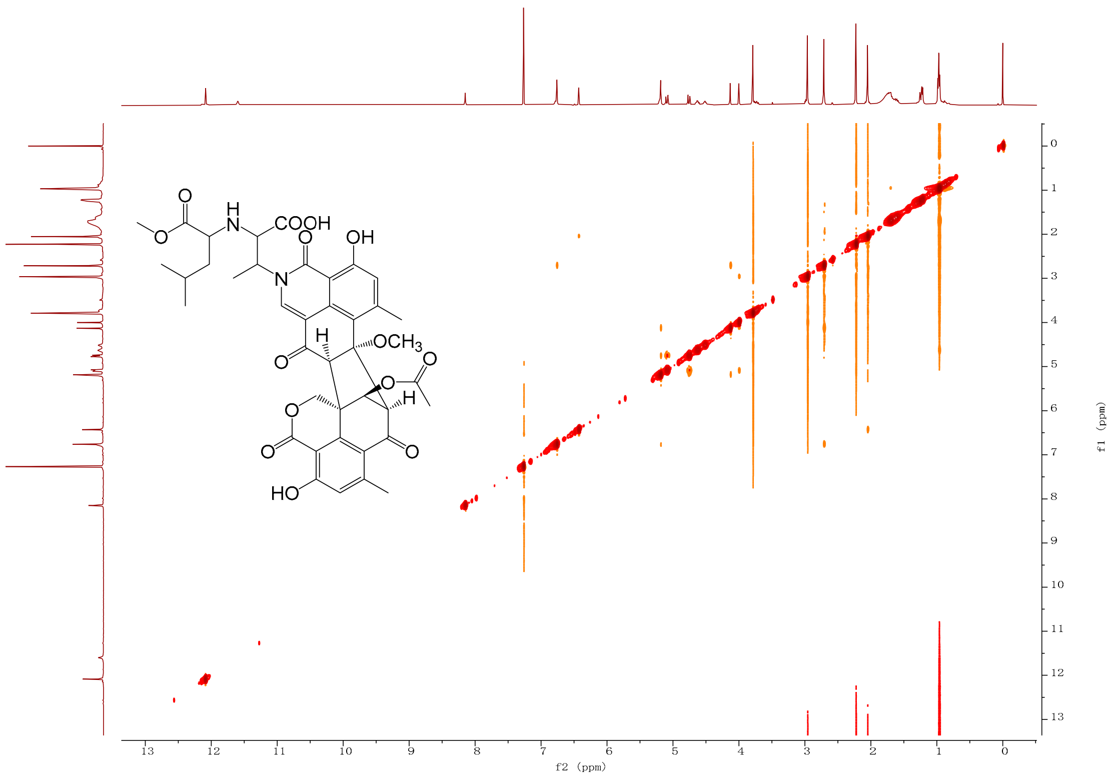

Figure S15 NOESY spectrum of **2** ($CDCl_3$)

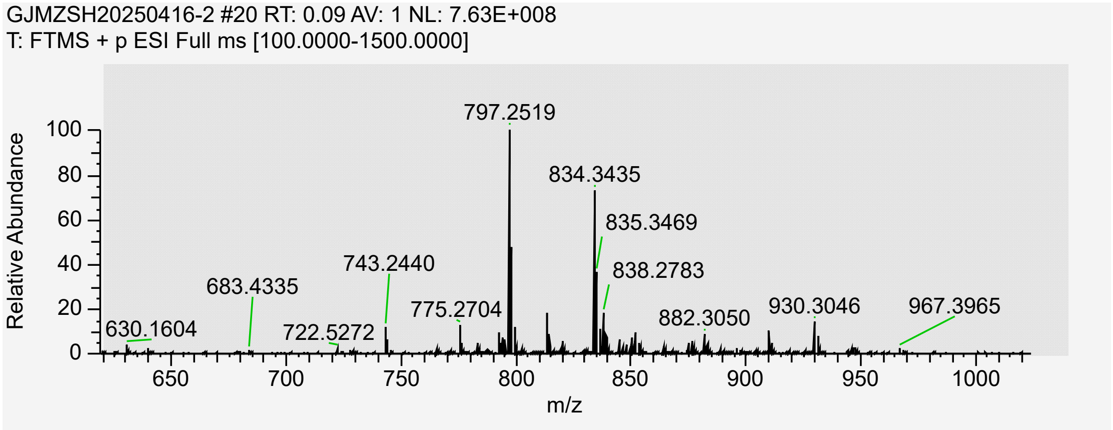

Figure S16 (+)-HRESIMS of compound **2**.

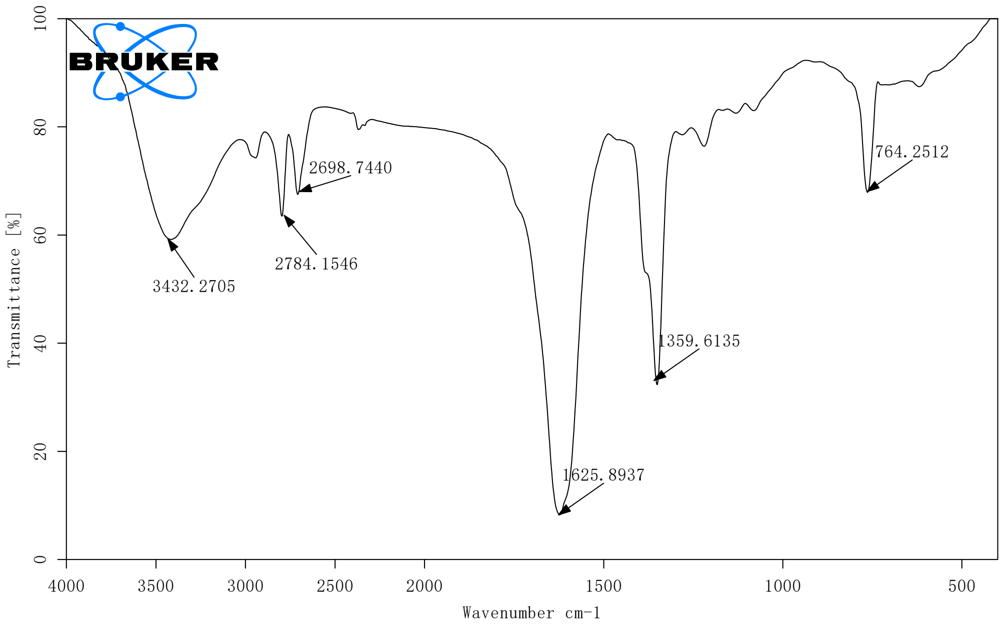

Figure S17 IR spectrum (film on KBr pellet) of compound **2**.

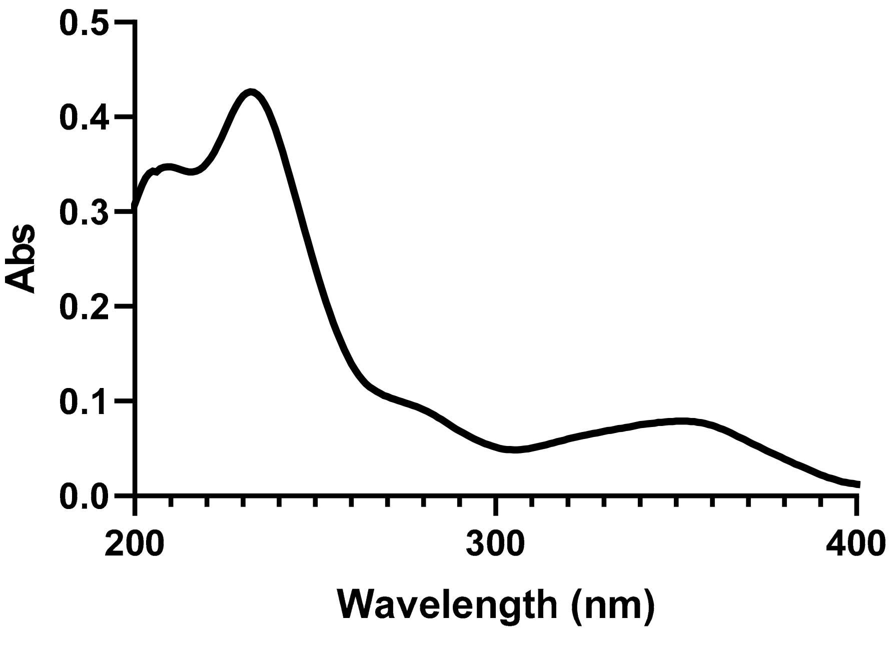

Figure S18 UV spectrum of compound **2** (MeOH).

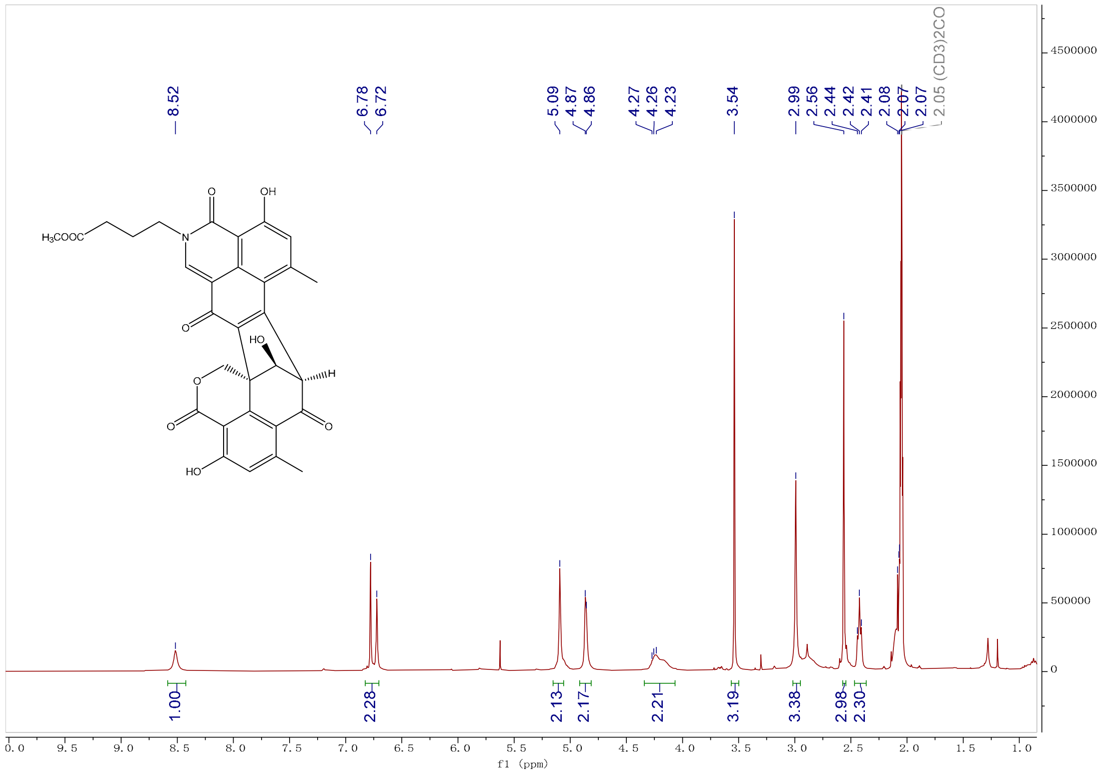

Figure S19 ^{1}H NMR spectrum of **3** (400 MHz, Acetone-d_6)

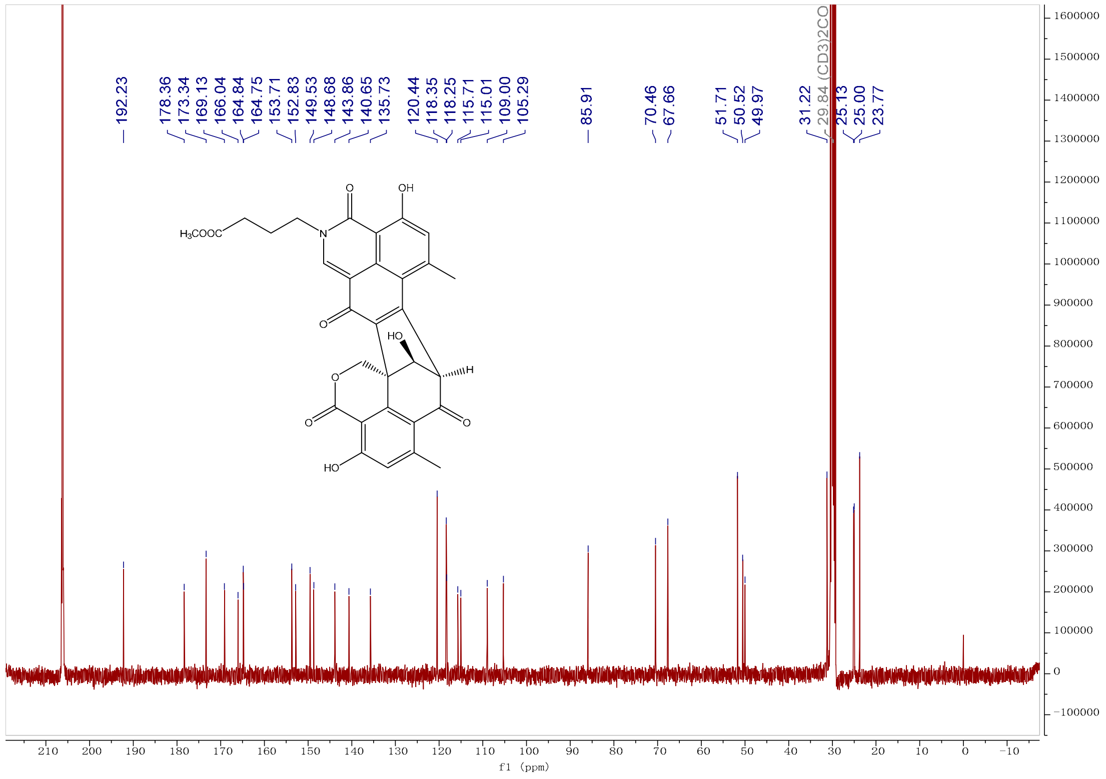

Figure S20 ^{13}CNMR spectrum of **3** (100 MHz, Acetone-d_6)

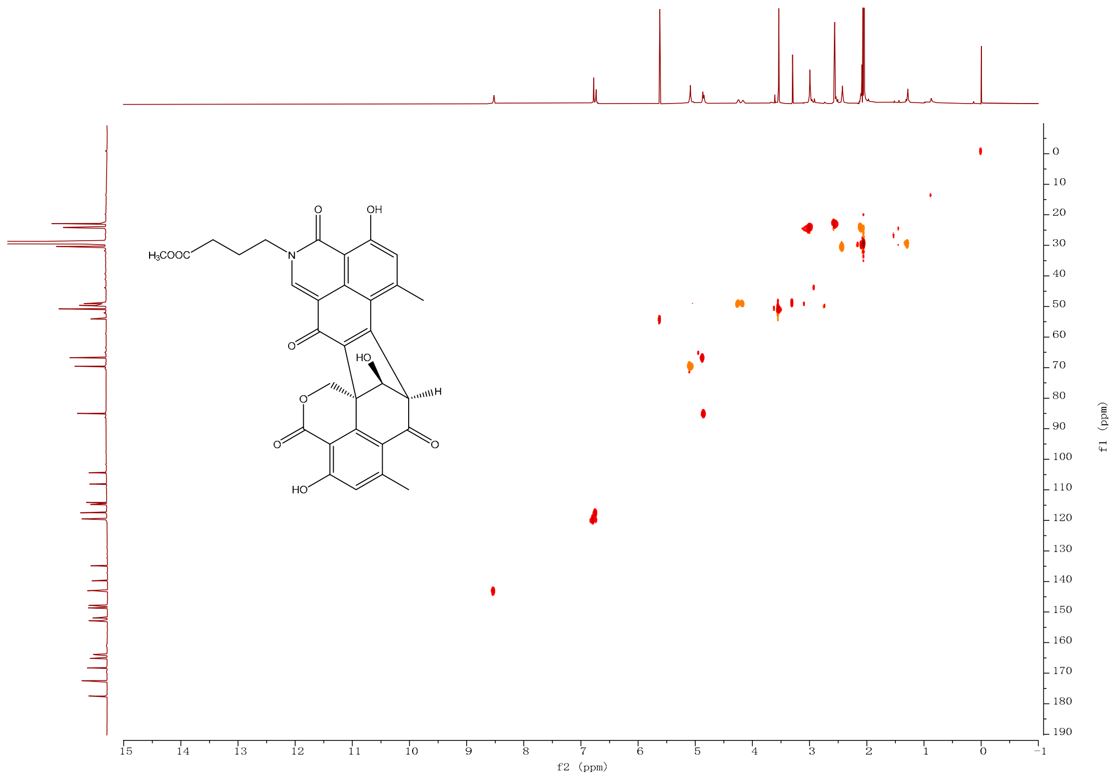

Figure S21 HSQC spectrum of **3** (Acetone-d_6)

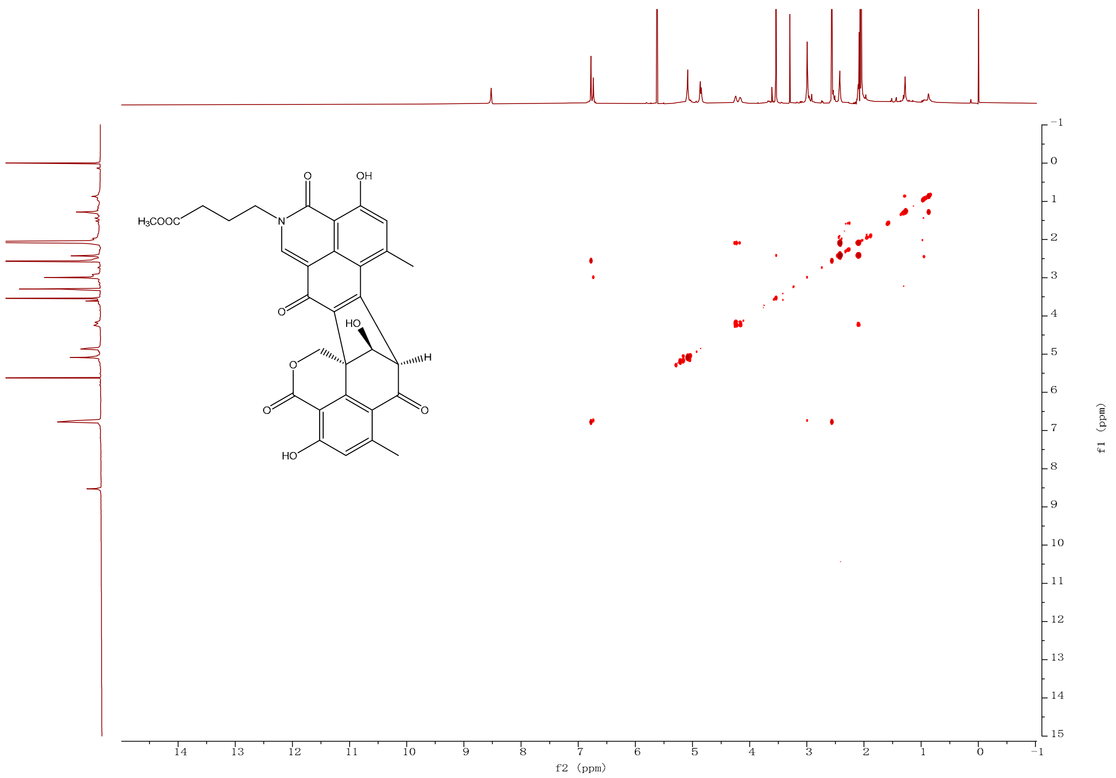

Figure S22 ^{1}H-^{1}H COSY spectrum of **3** (Acetone-d_6)

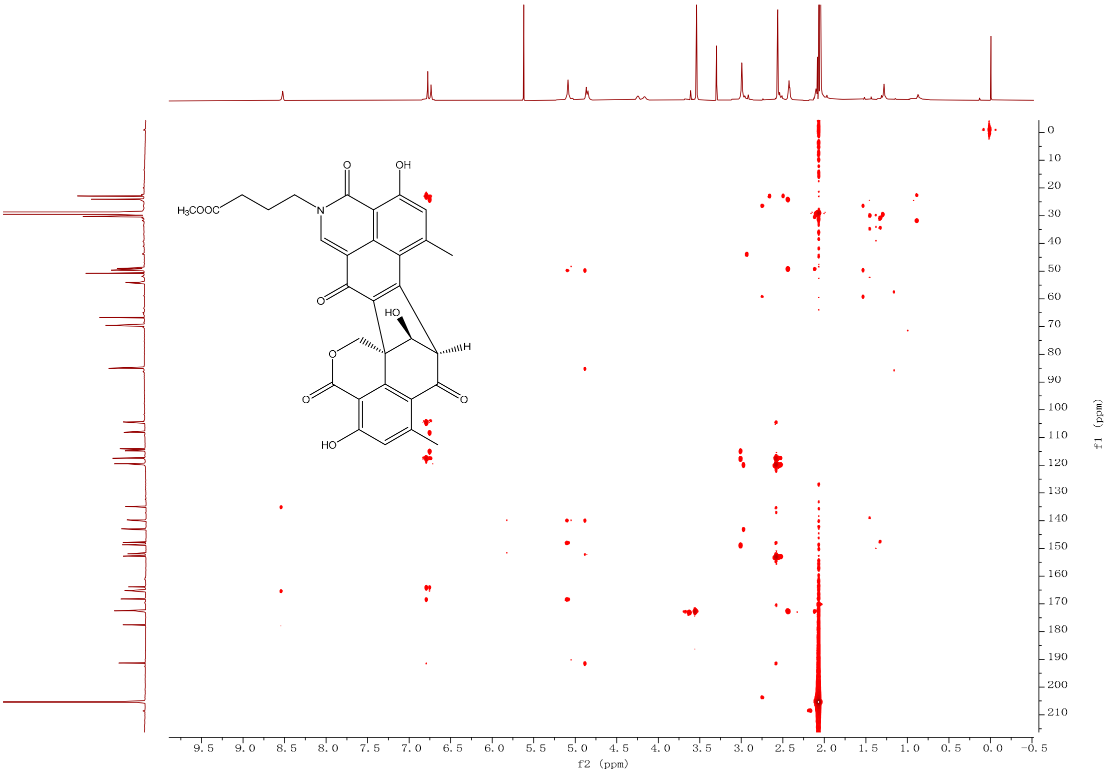

Figure S23 HMBC spectrum of **3** (Acetone-d_6)

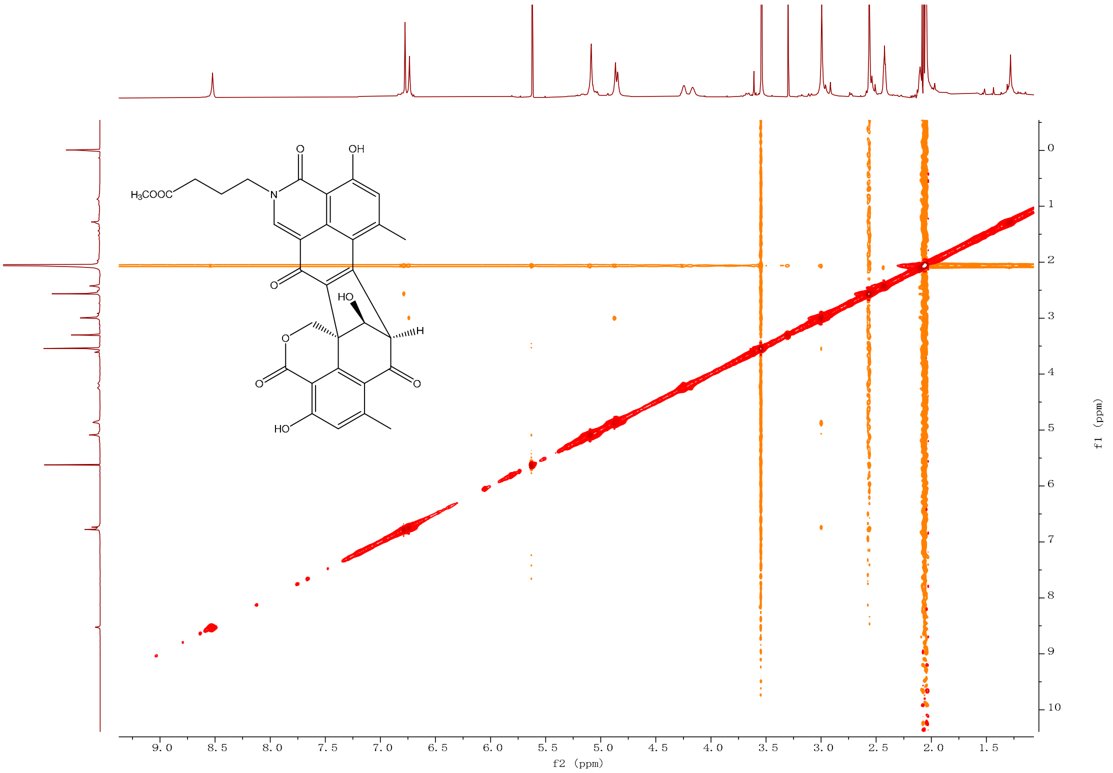

Figure S24 NOESY spectrum of **3** (Acetone-d_6)

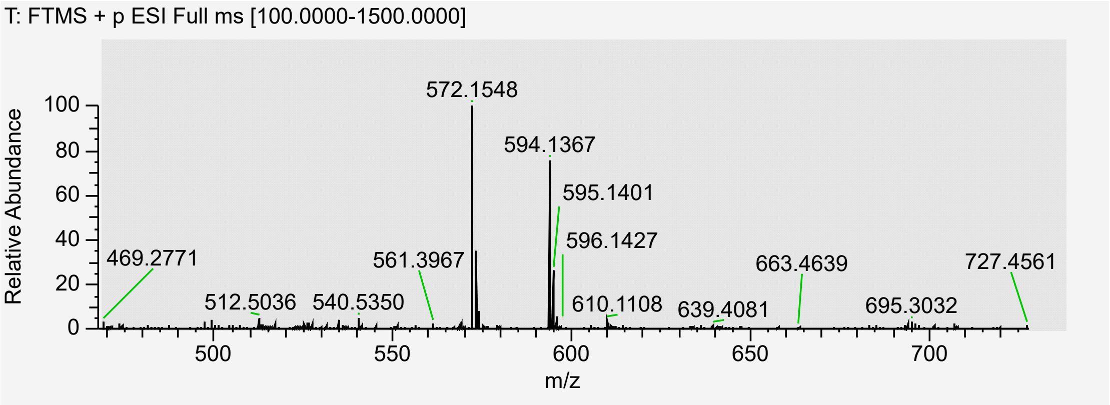

Figure S25 (+)-HRESIMS of compound **3**.

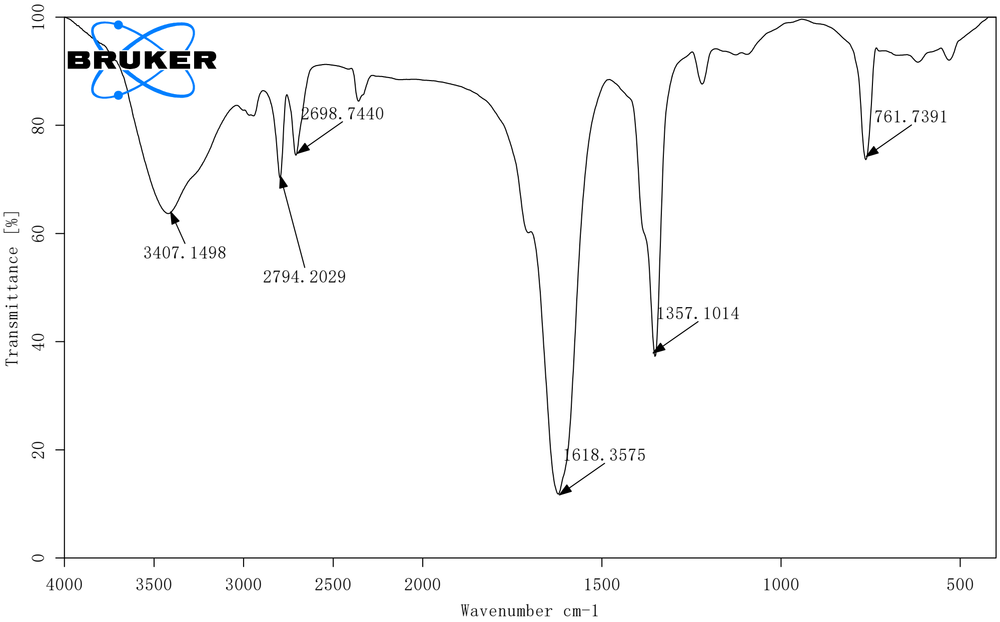

Figure S26 IR spectrum (film on KBr pellet) of compound **3**.

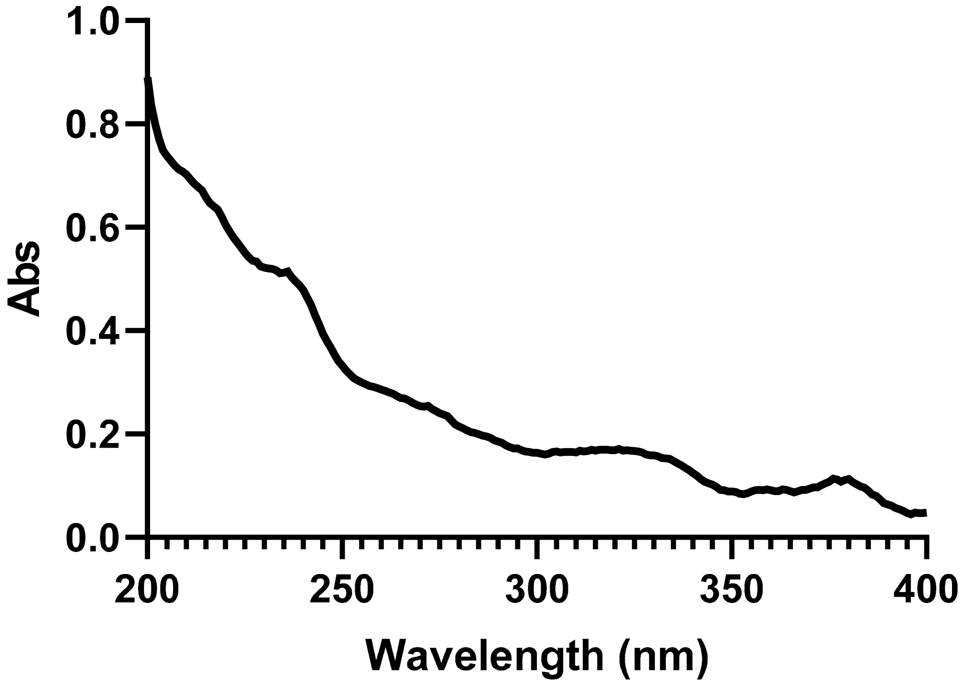

Figure S27 UV spectrum of compound **3** (MeOH).

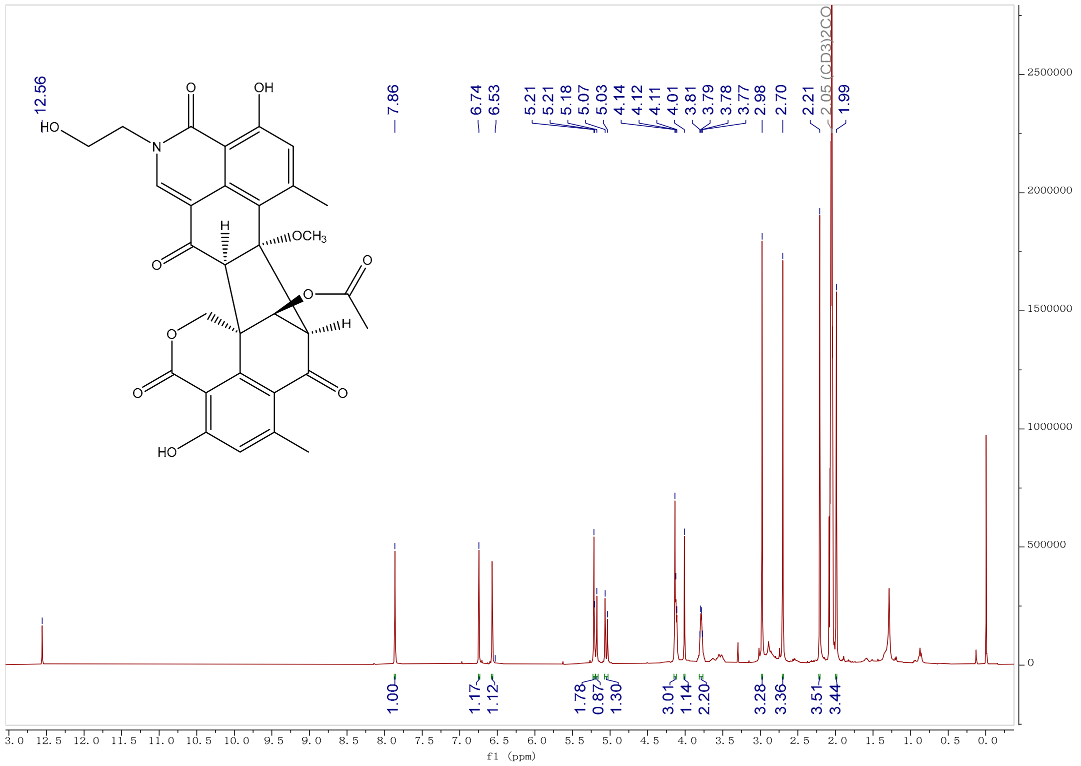

Figure S28 ^{1}H NMR spectrum of **4** (400 MHz, Acetone-d_6)

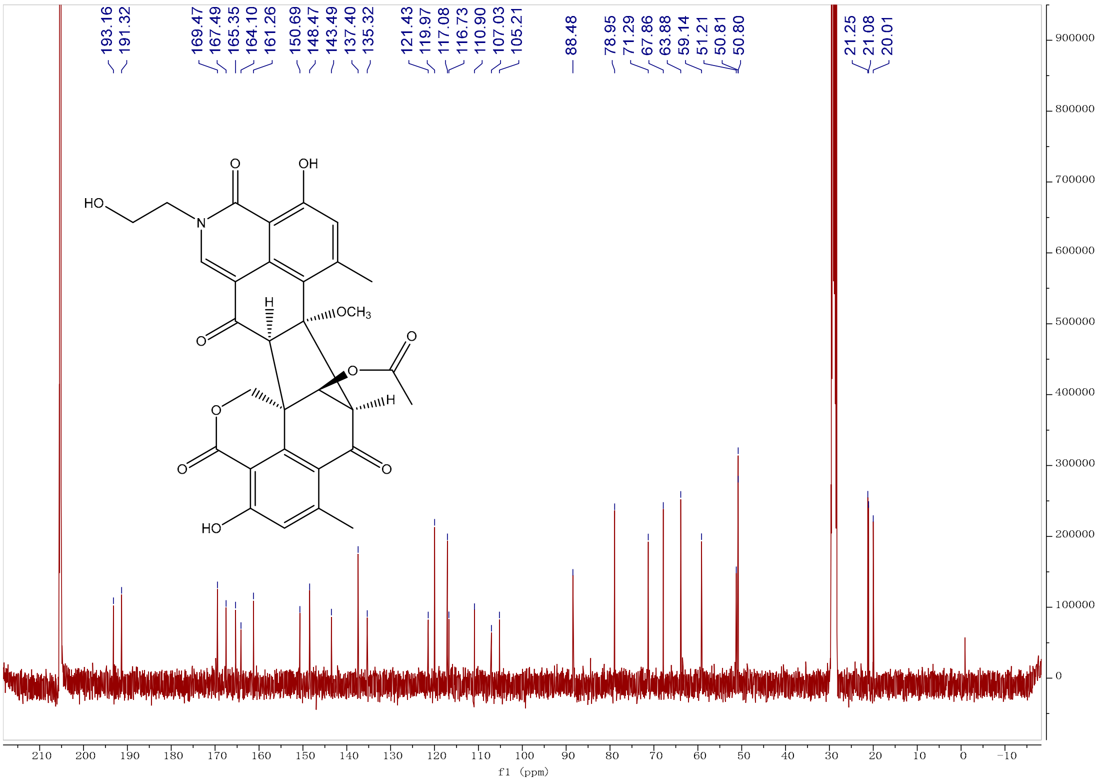

Figure S29 ^{13}CNMR spectrum of **4** (100 MHz, Acetone-d_6)

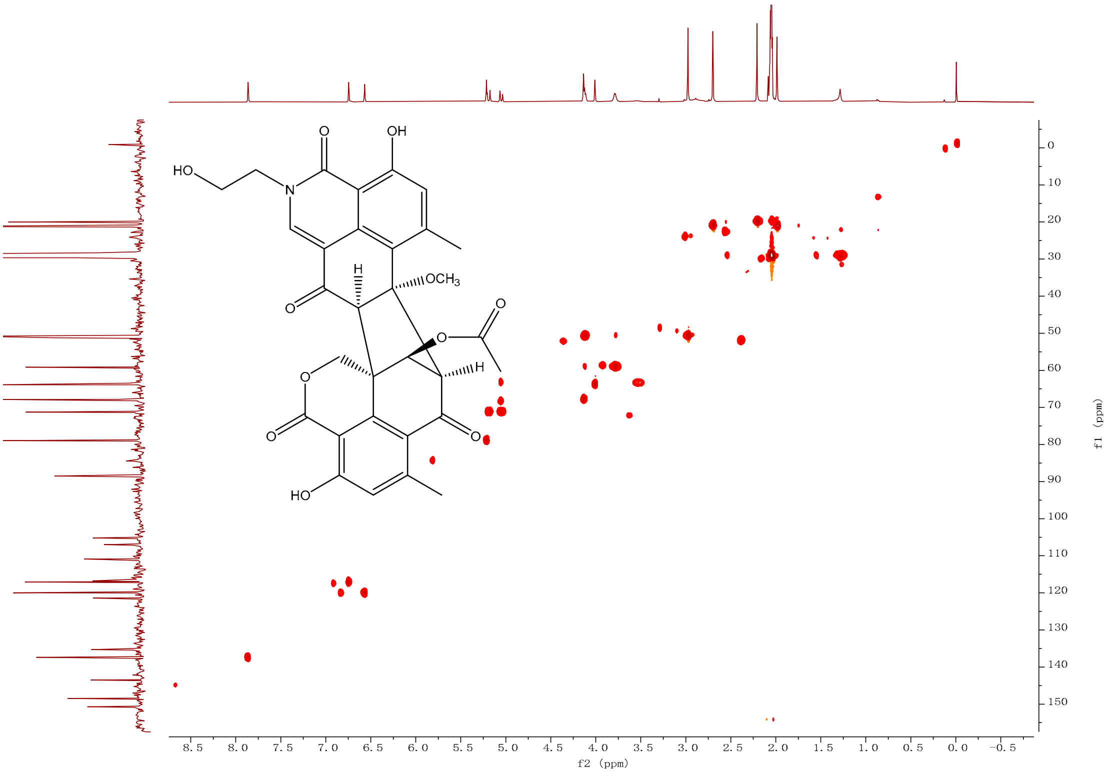

Figure S30 HSQC spectrum of **4** (Acetone-d_6)

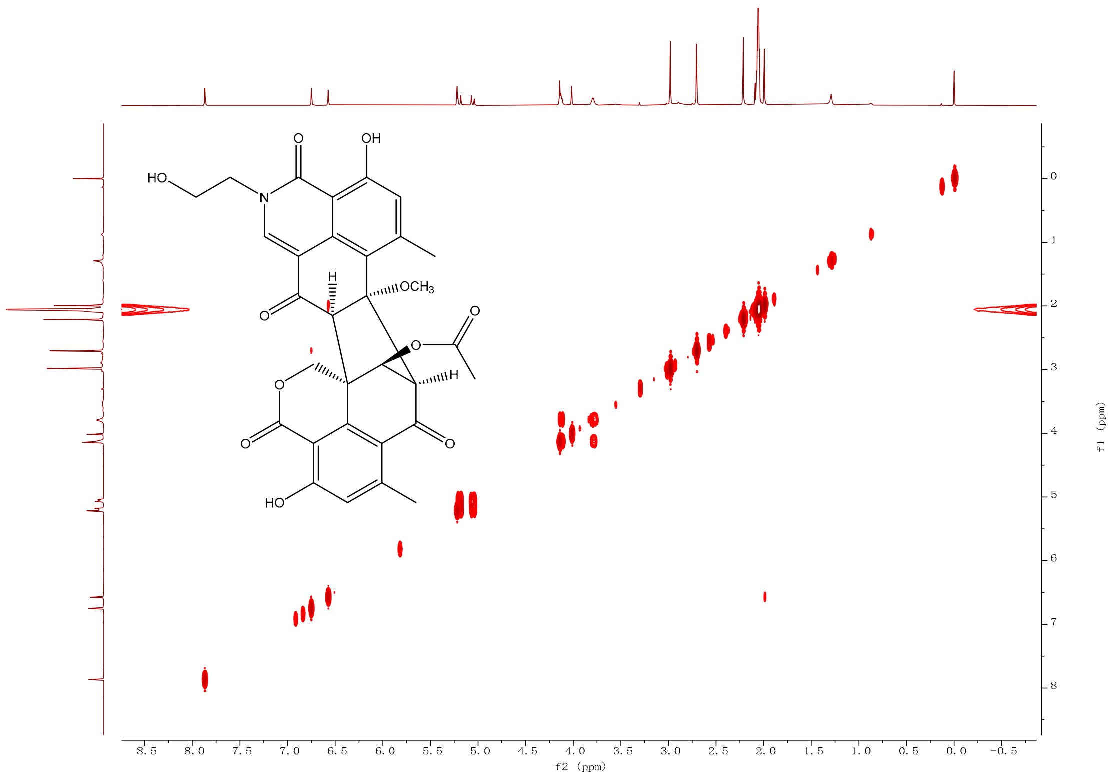

Figure S31 ^1H-^1H COSY spectrum of **4** (Acetone-*d*6)

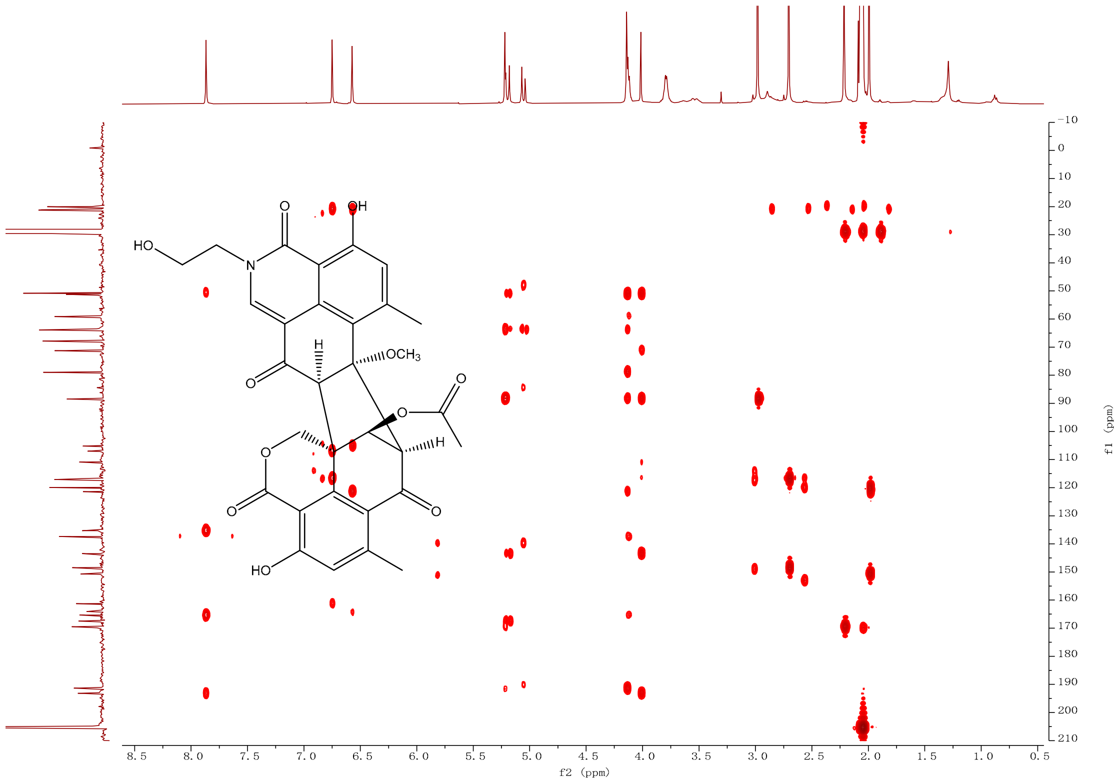

Figure S32 HMBC spectrum of 4 (Acetone-*d*6)

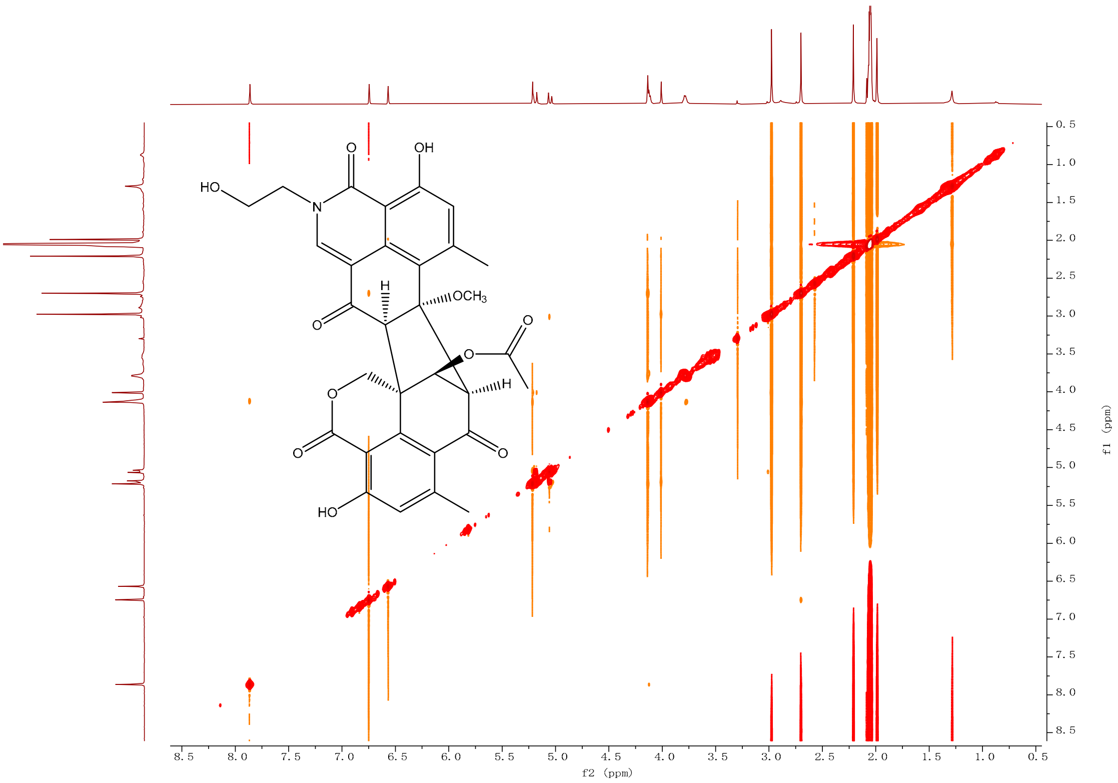

Figure S33 NOESY spectrum of **4** (Acetone-d_6)

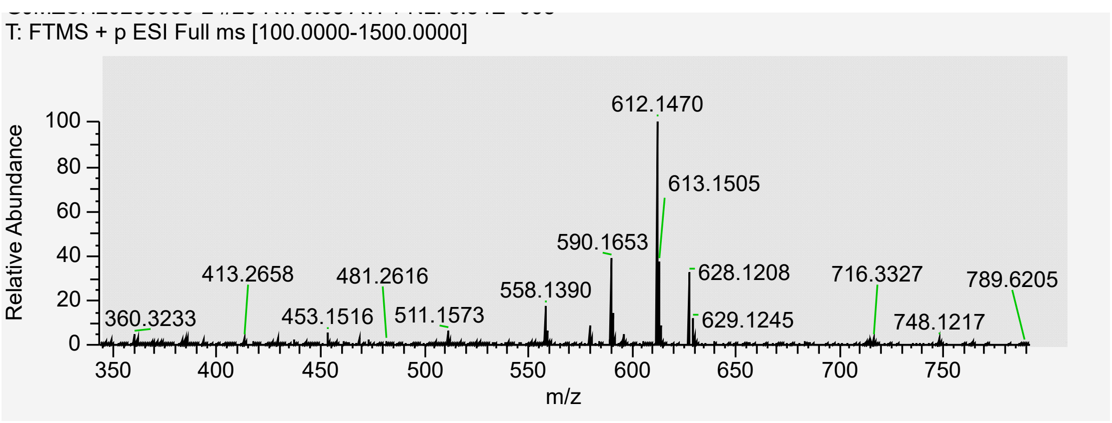

Figure S34 (+)-HRESIMS of compound **4**.

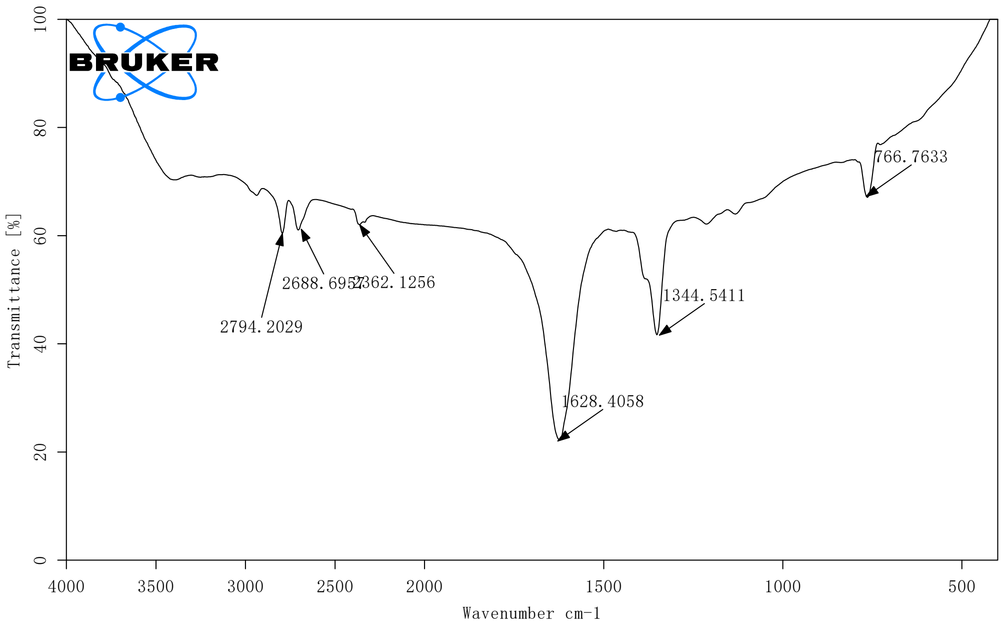

Figure S35 IR spectrum (film on KBr pellet) of compound **4**.

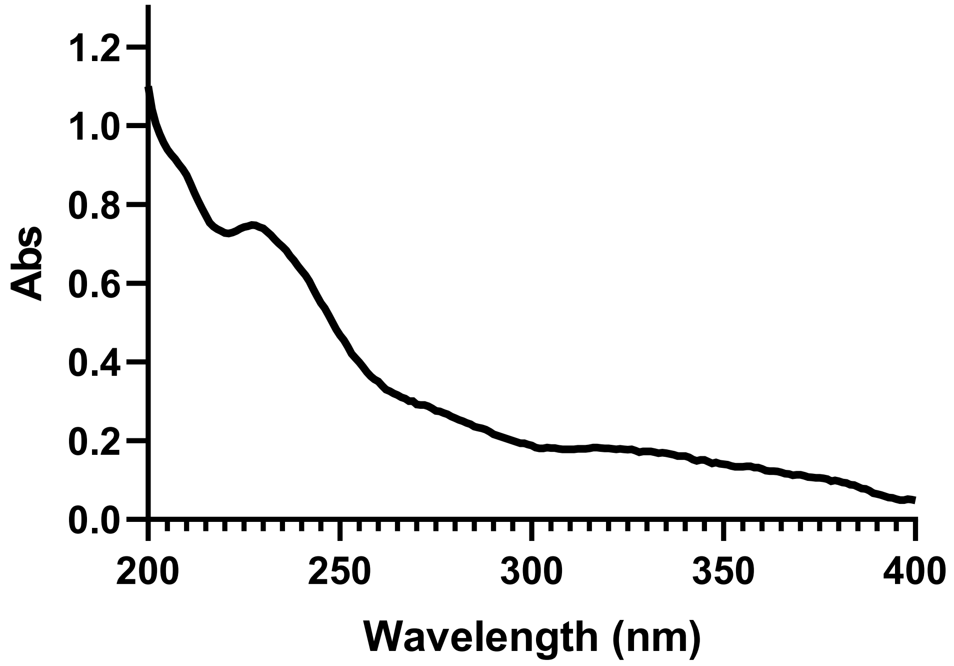

Figure S36 UV spectrum of compound **4** (MeOH).

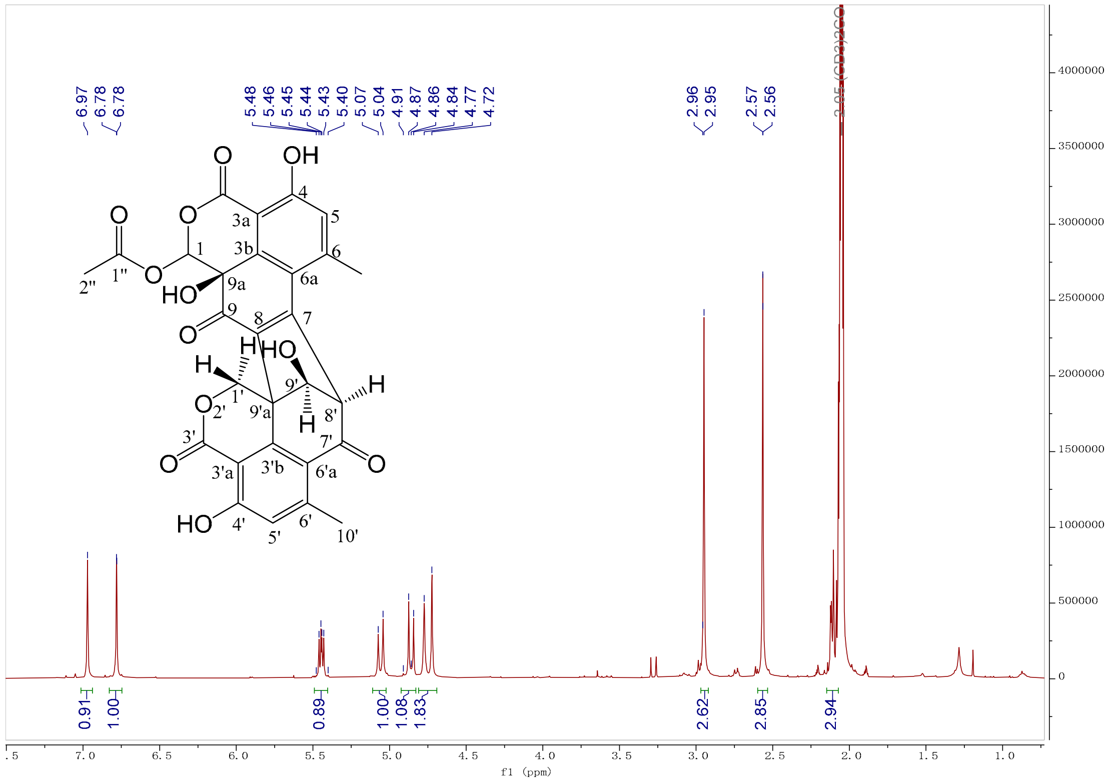

Figure S37 ^{1}H NMR spectrum of **5** (400 MHz, Acetone-d_6)

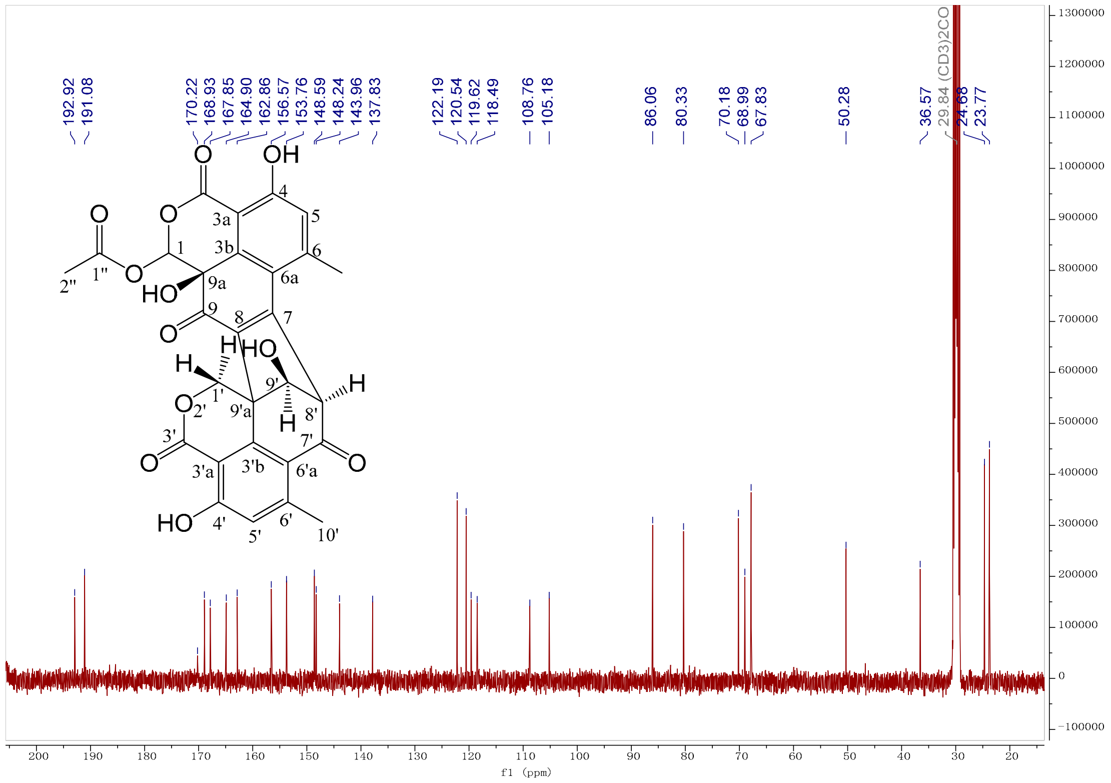

Figure S38 ^{13}CNMR spectrum of **5** (100 MHz, Acetone-d_6)

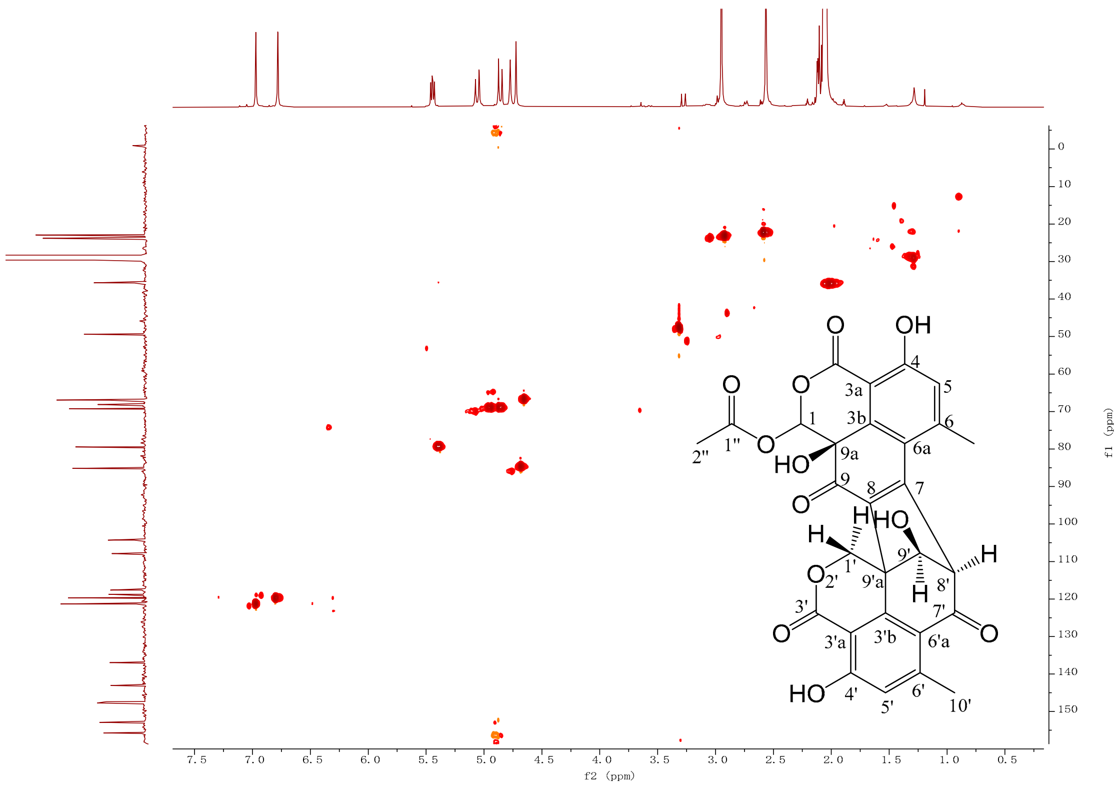

Figure S39 HSQC spectrum of **5** (Acetone-d_6)

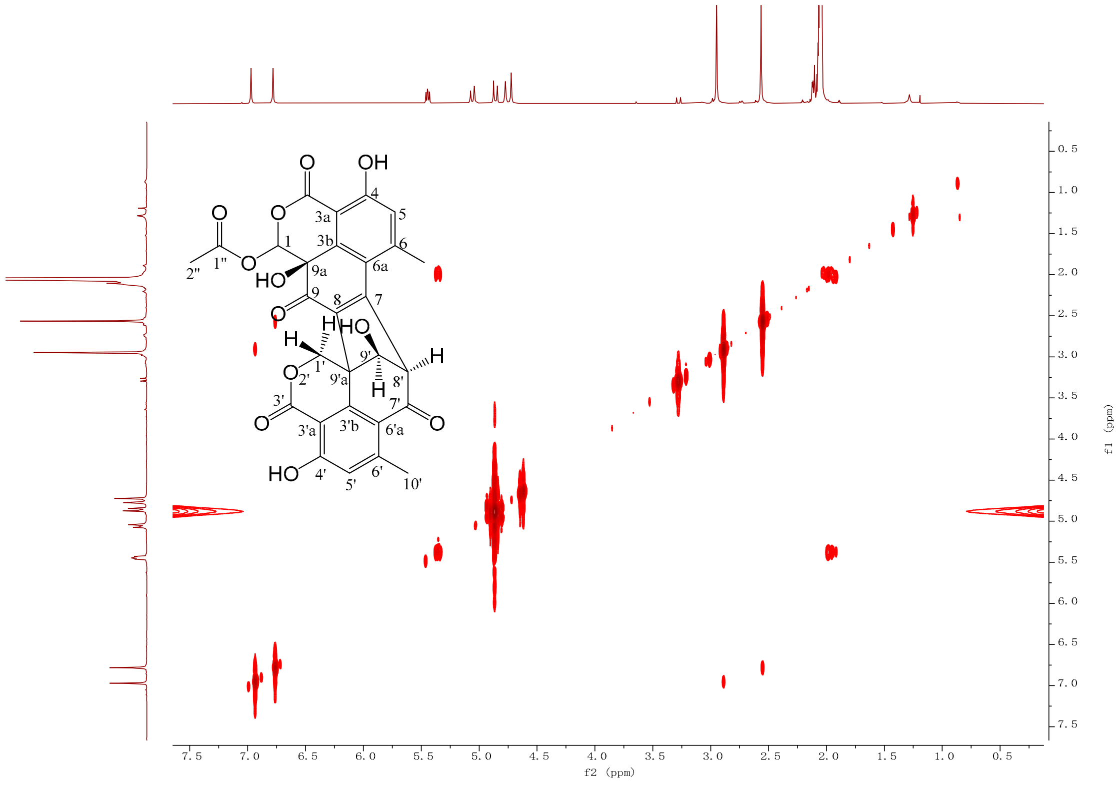

Figure S40 ^{1}H-^{1}H COSY spectrum of **5** (Acetone-d_6)

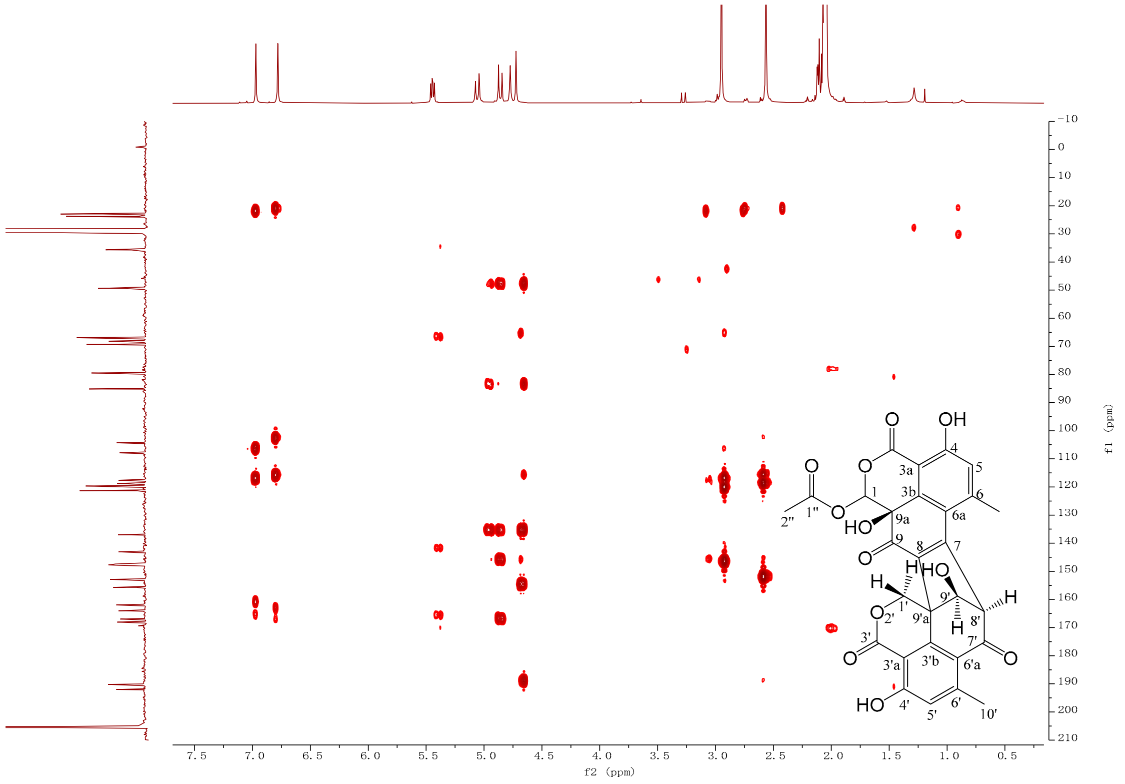

Figure S41 HMBC spectrum of **5** (Acetone-d_6)

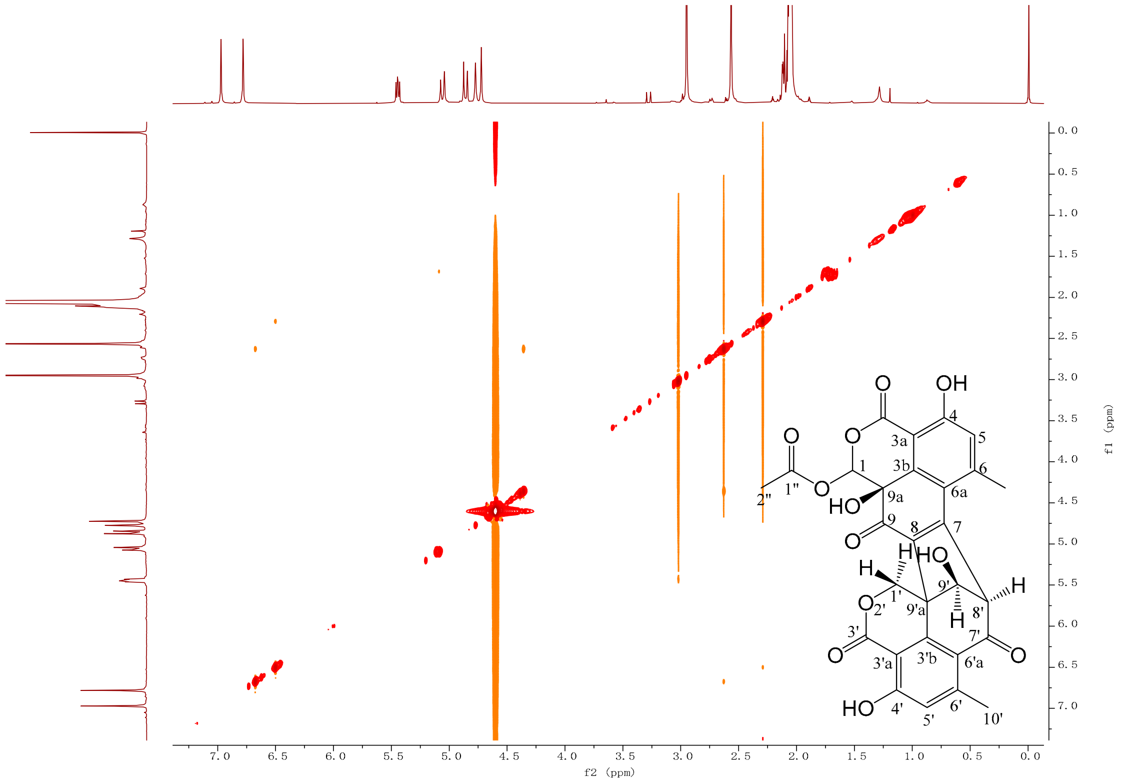

Figure S42 NOESY spectrum of **5** (Acetone-d_6)

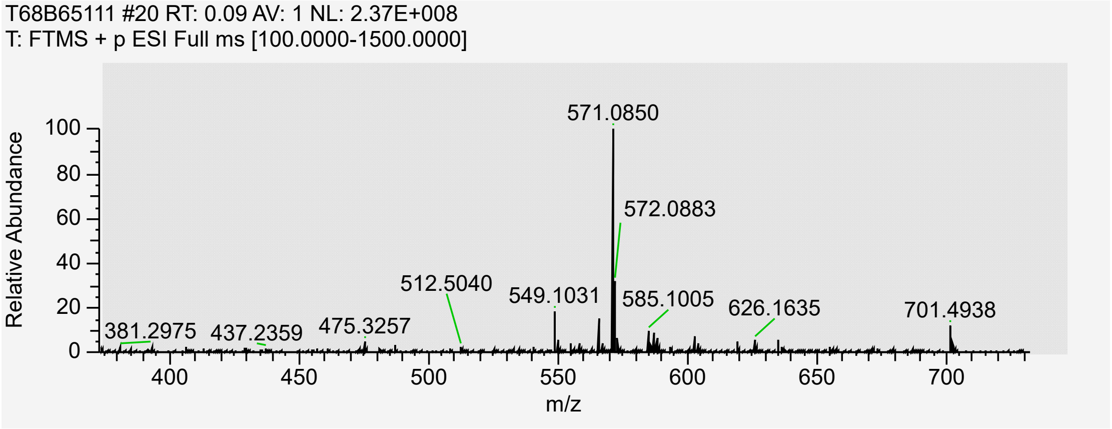

Figure S43 (+)-HRESIMS of compound **5**.

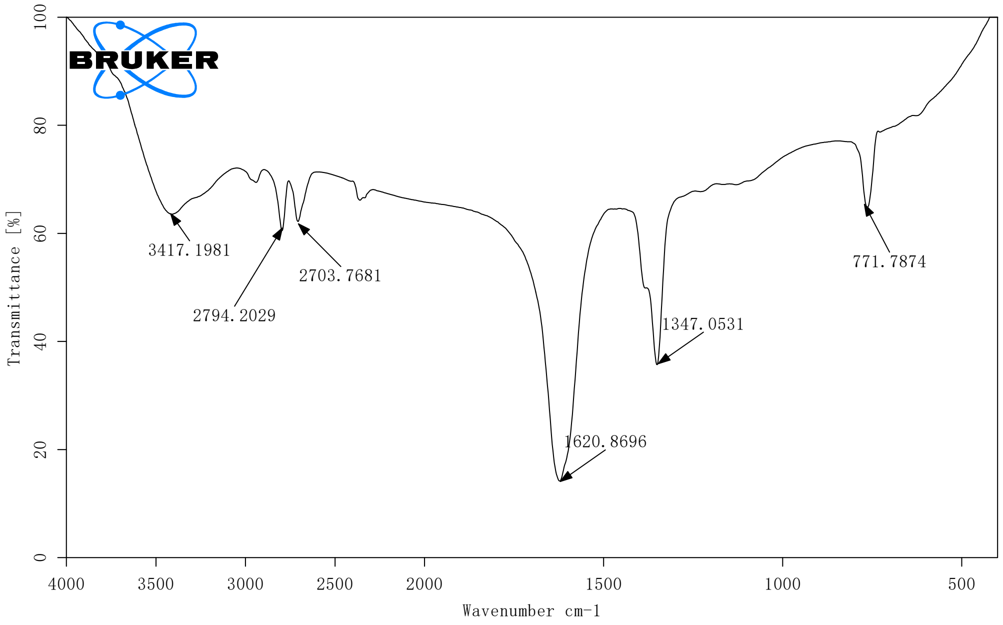

Figure S44 IR spectrum (film on KBr pellet) of compound **5**.

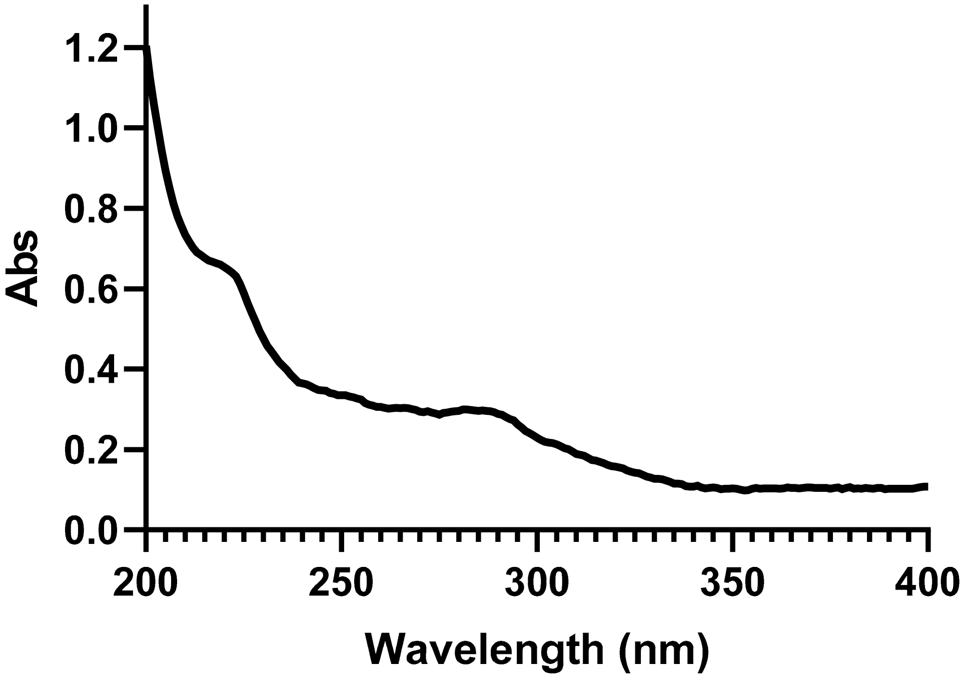

Figure S45 UV spectrum of compound **5** (MeOH).

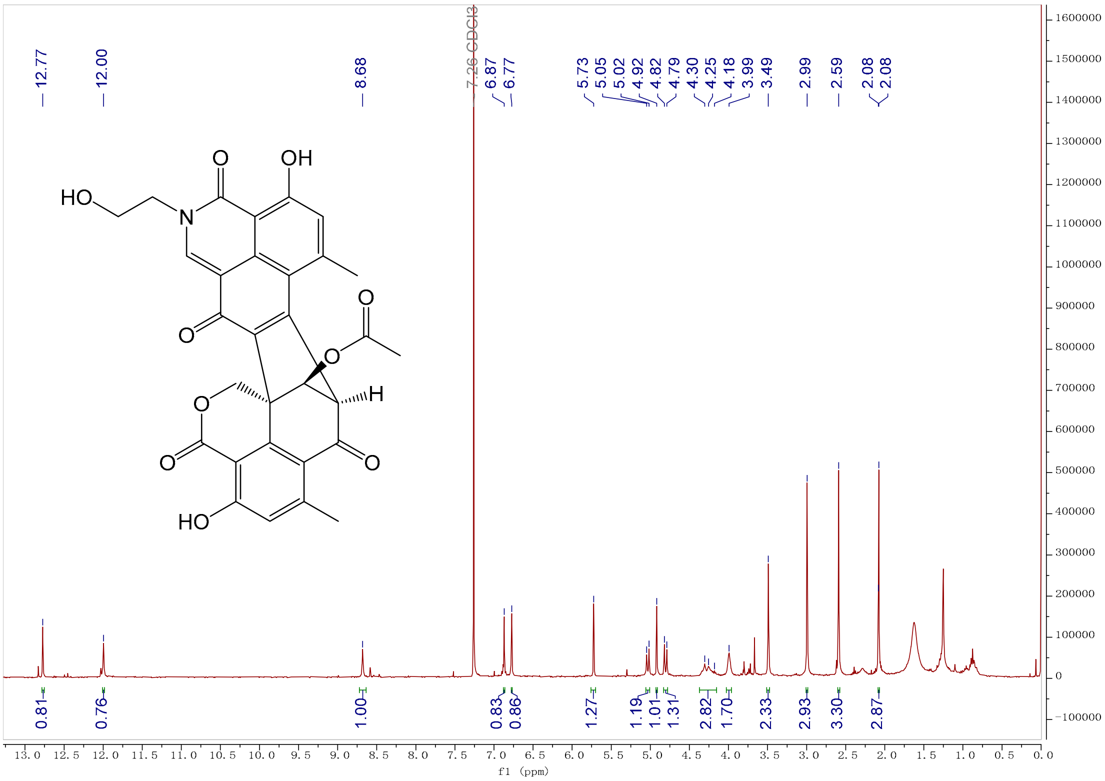

Figure S46 ^{1}H NMR spectrum of compound **6** (400 MHz, $CDCl_3$).

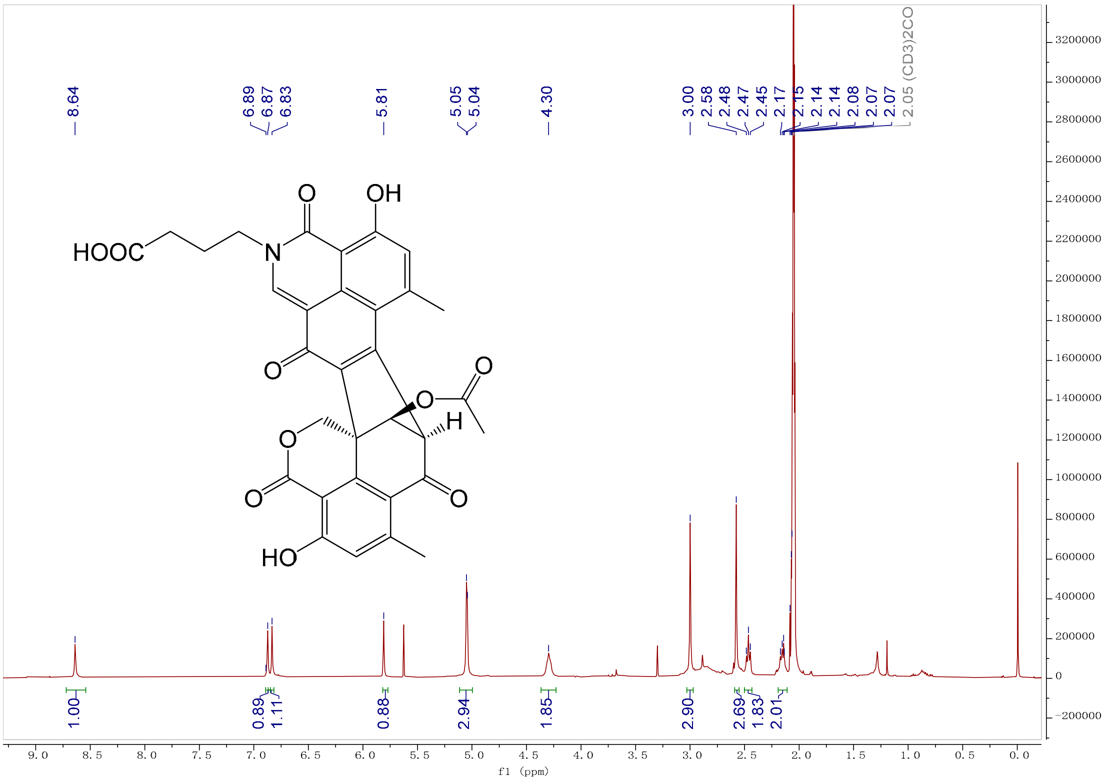

Figure S47 ^{1}H NMR spectrum of compound **7** (400 MHz, Acetone-d_6).

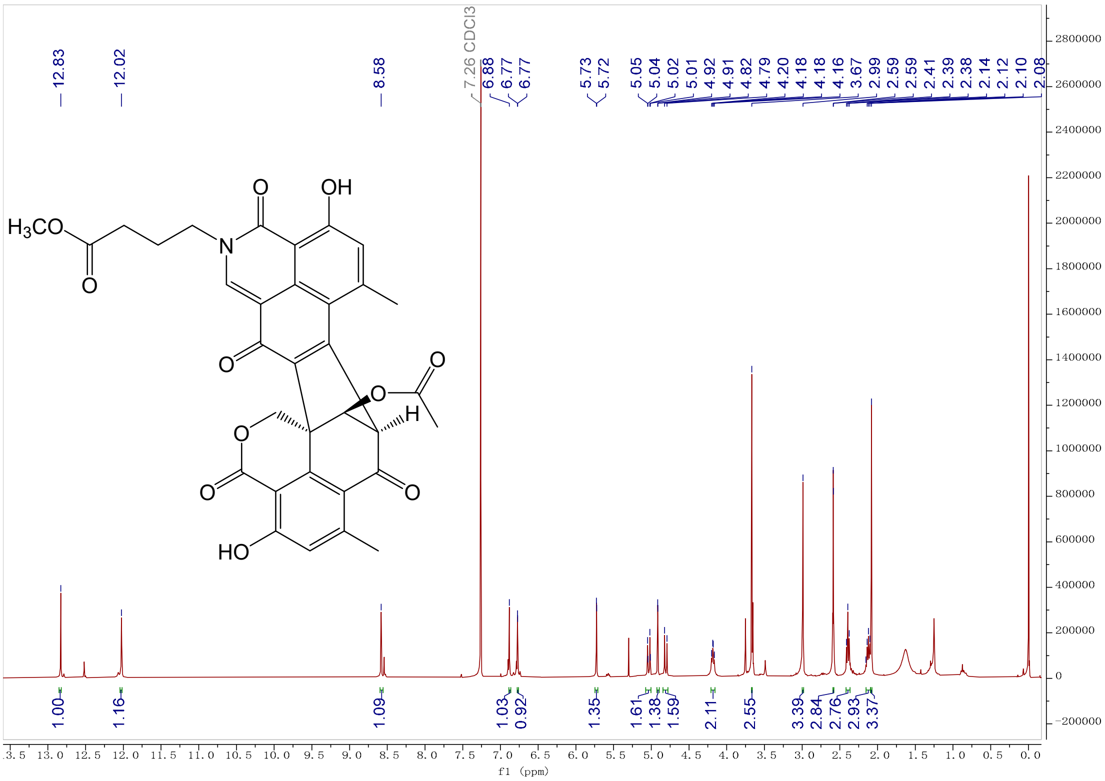

Figure S48 ^{1}H NMR spectrum of compound **8** (400 MHz, $CDCl_3$).

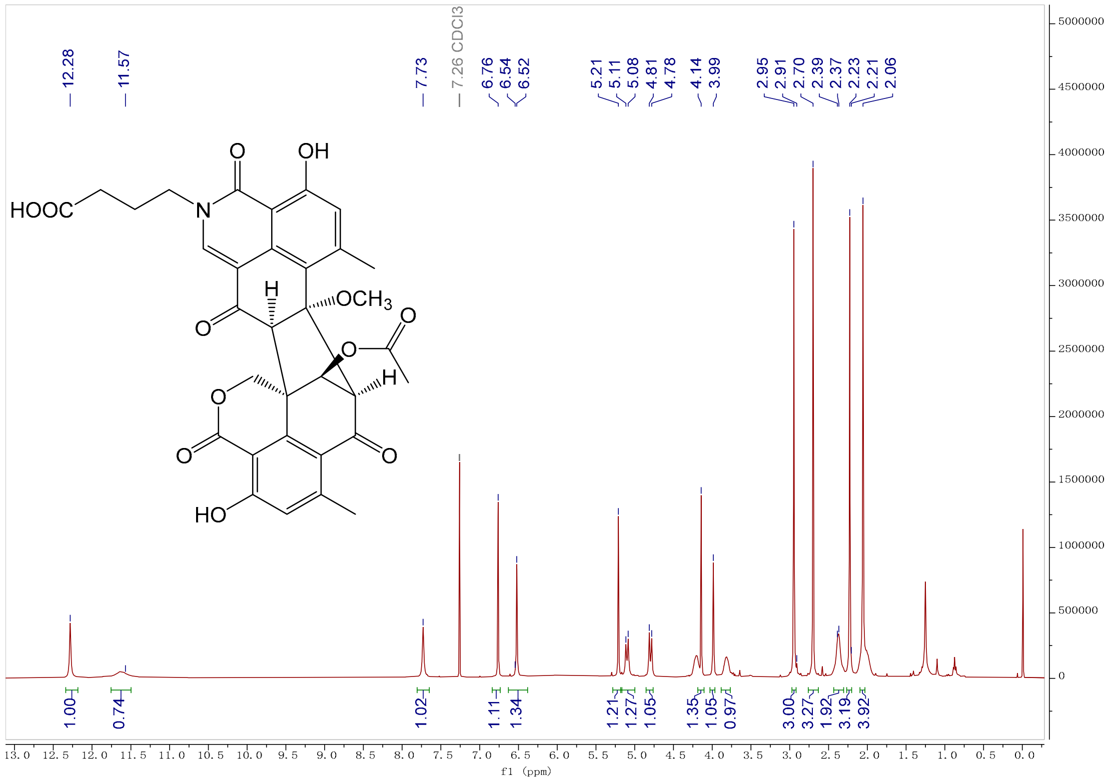

Figure S49 ^{1}H NMR spectrum of compound **9** (400 MHz, $CDCl_3$).

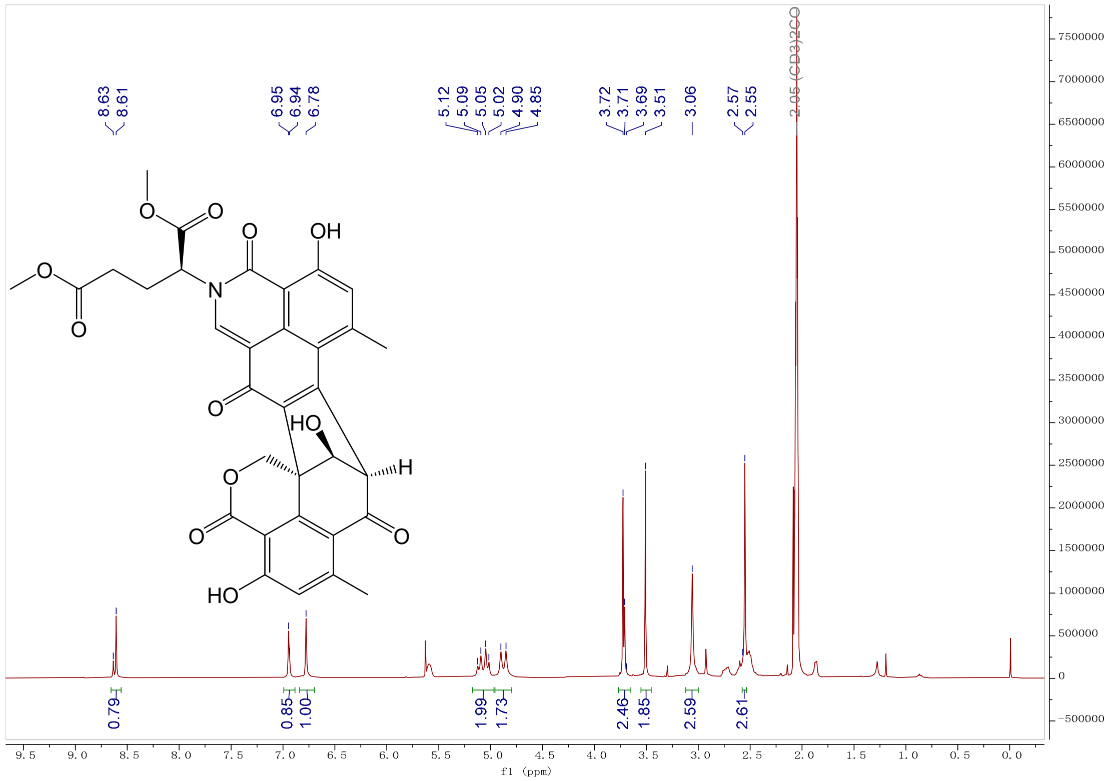

Figure S50 ^{1}H NMR spectrum of compound **10** (400 MHz, Acetone-d_6).

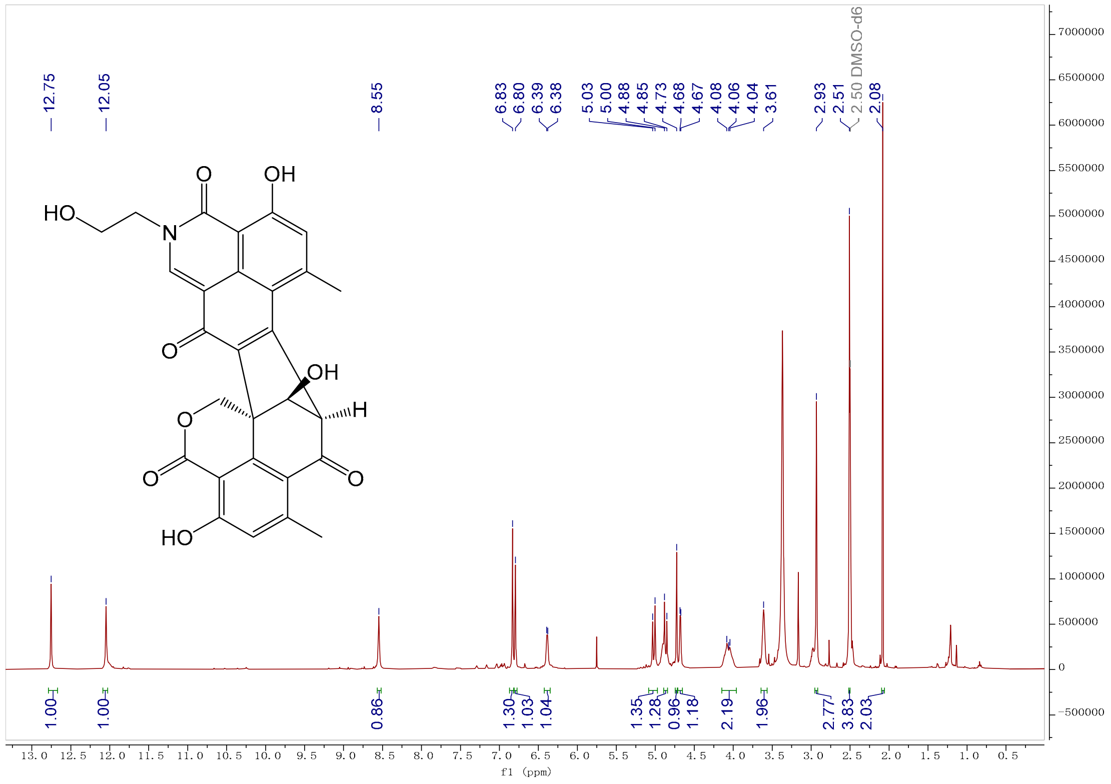

Figure S51 ^{1}H NMR spectrum of compound **11** (400 MHz, Acetone-d_6).

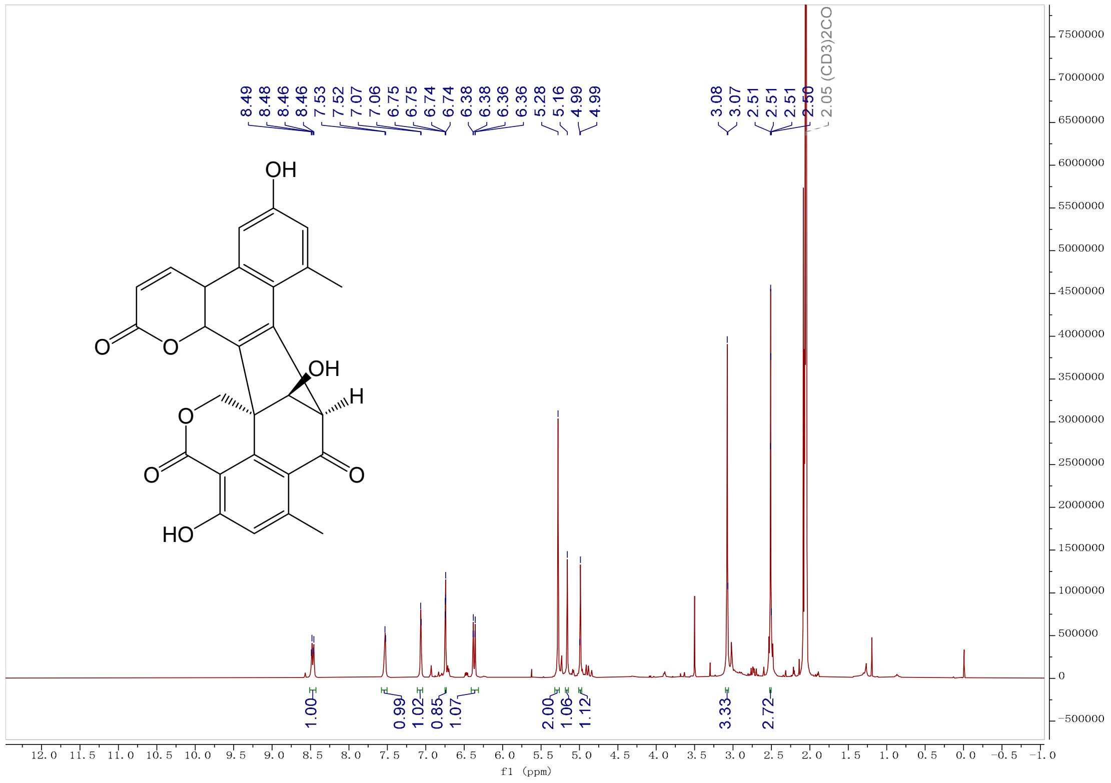

Figure S52 ^{1}H NMR spectrum of compound **12** (400 MHz, Acetone-d_6).

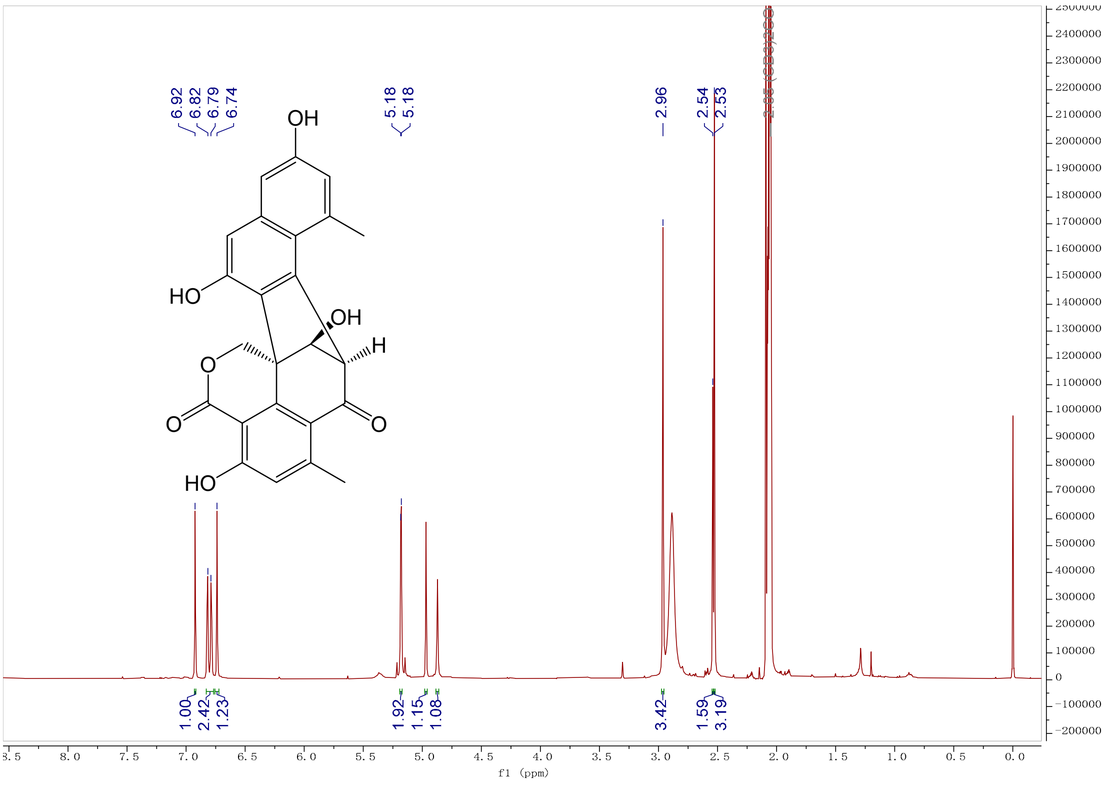

Figure S53 ^{1}H NMR spectrum of compound **13** (400 MHz, Acetone-d_6).

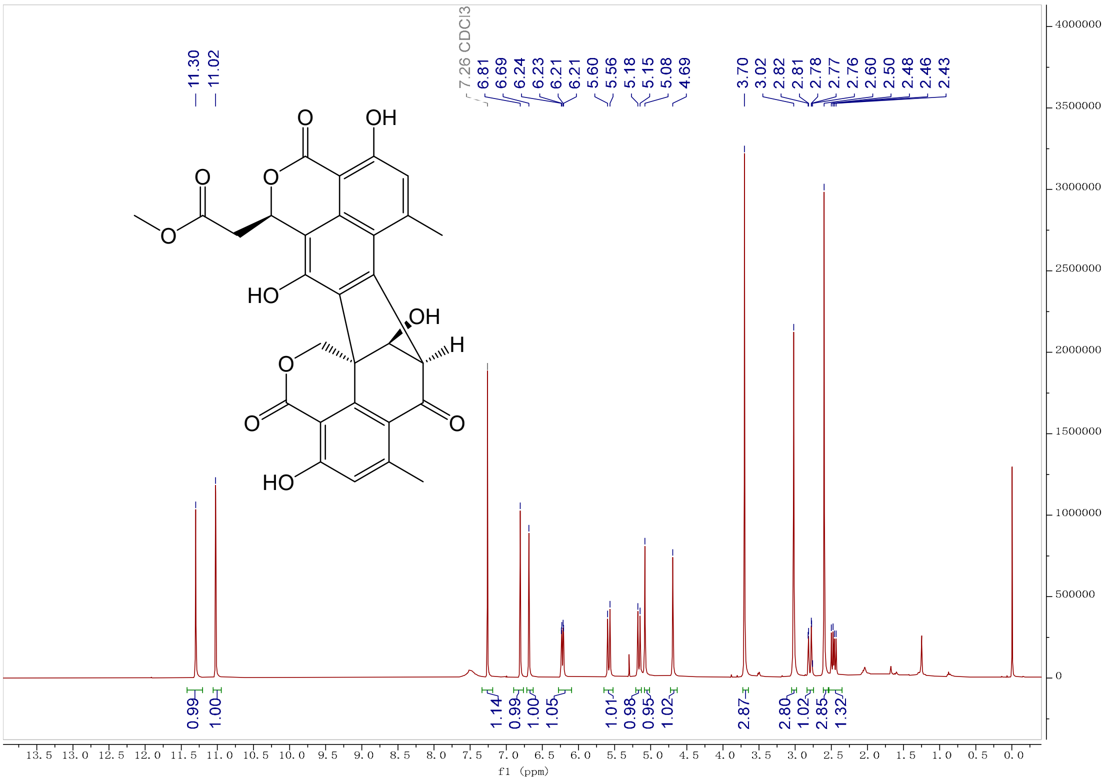

Figure S54 ^{1}H NMR spectrum of compound **14** (400 MHz, CDCl$_3$).

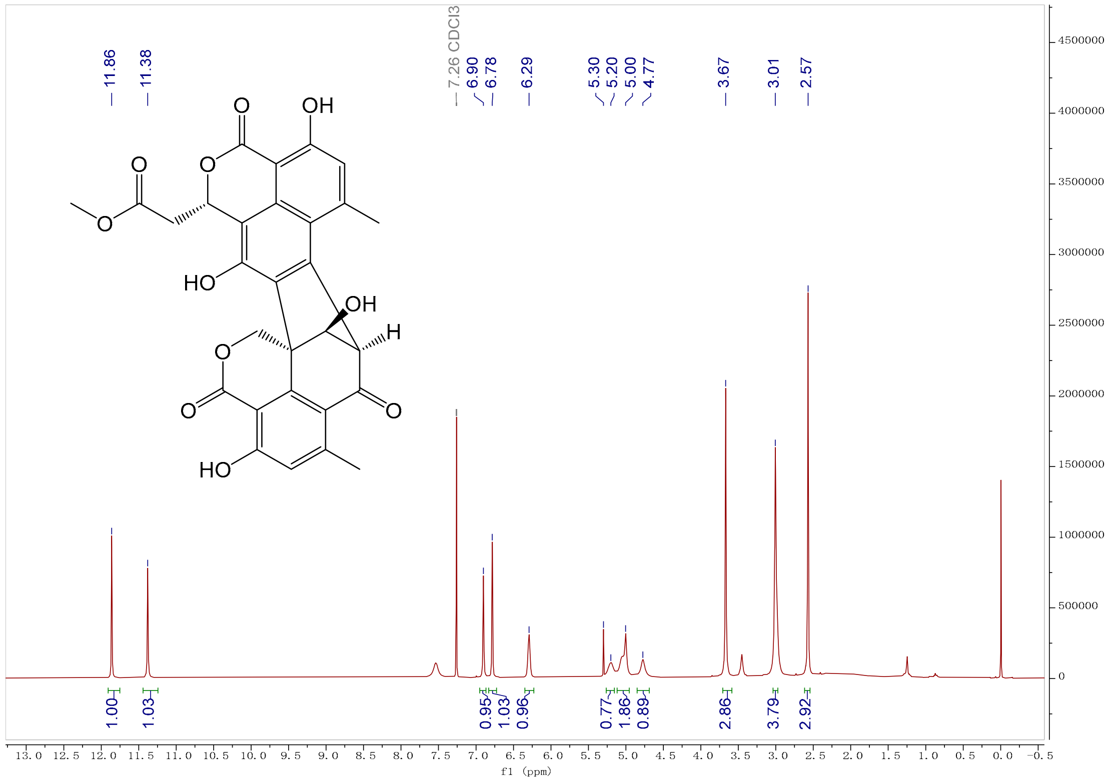

Figure S55 ^{1}H NMR spectrum of compound **15** (400 MHz, $CDCl_3$).

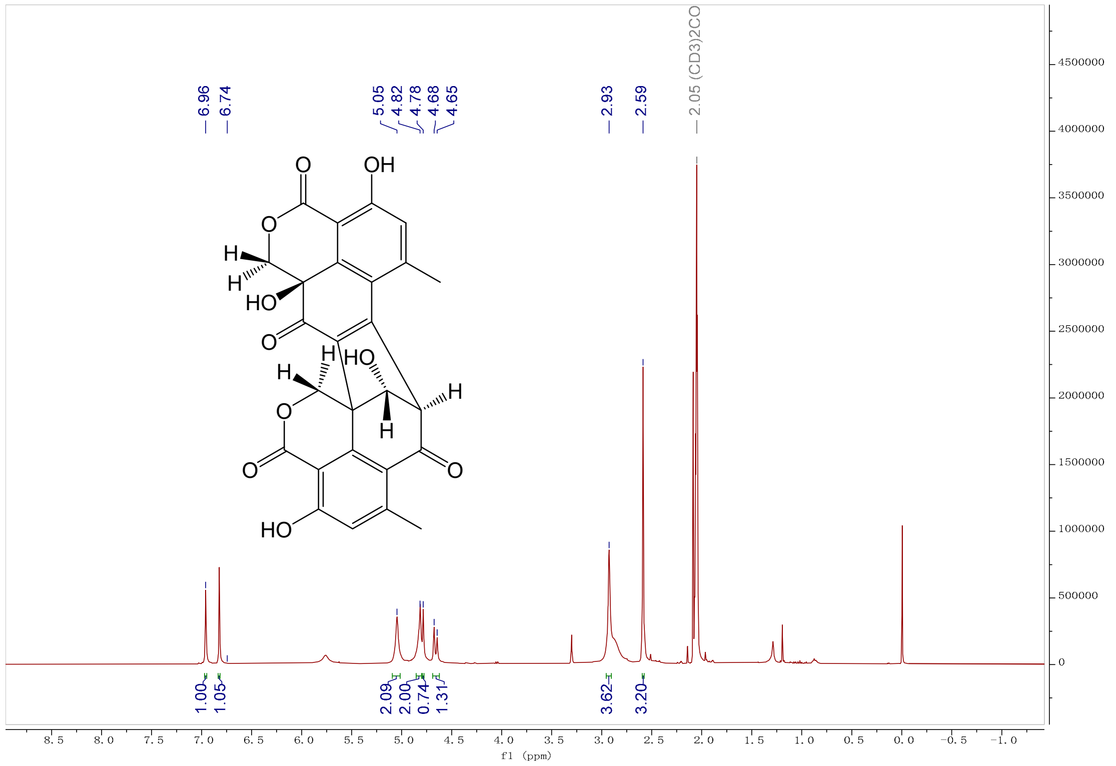

Figure S56 ^{1}H NMR spectrum of compound **16** (400 MHz, Acetone-d_6).

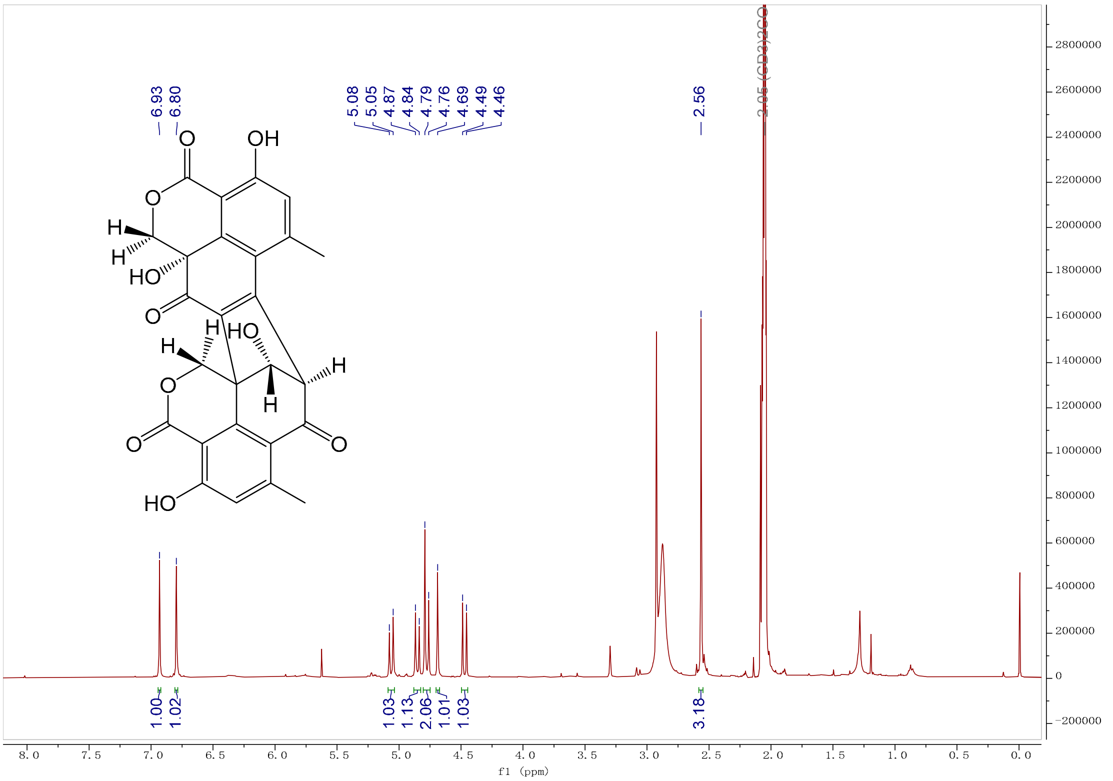

Figure S57 ^{1}H NMR spectrum of compound **17** (400 MHz, Acetone-d_6).

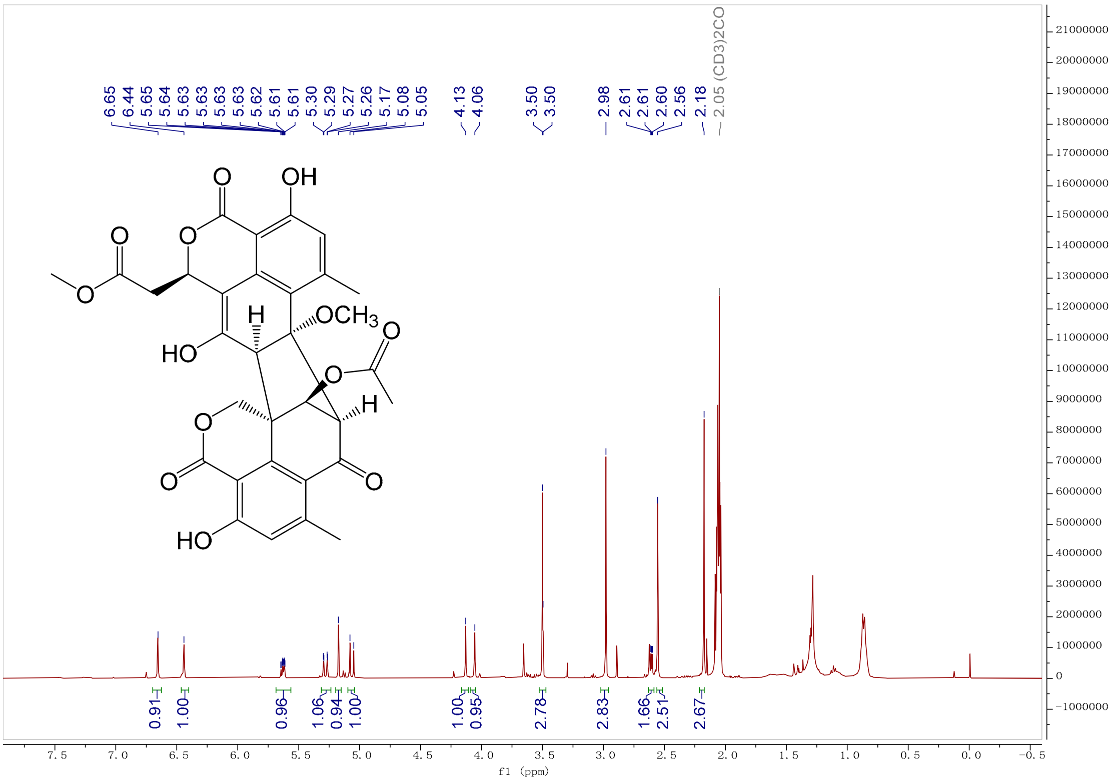

Figure S58 ^{1}H NMR spectrum of compound **18** (400 MHz, Acetone-d_6).

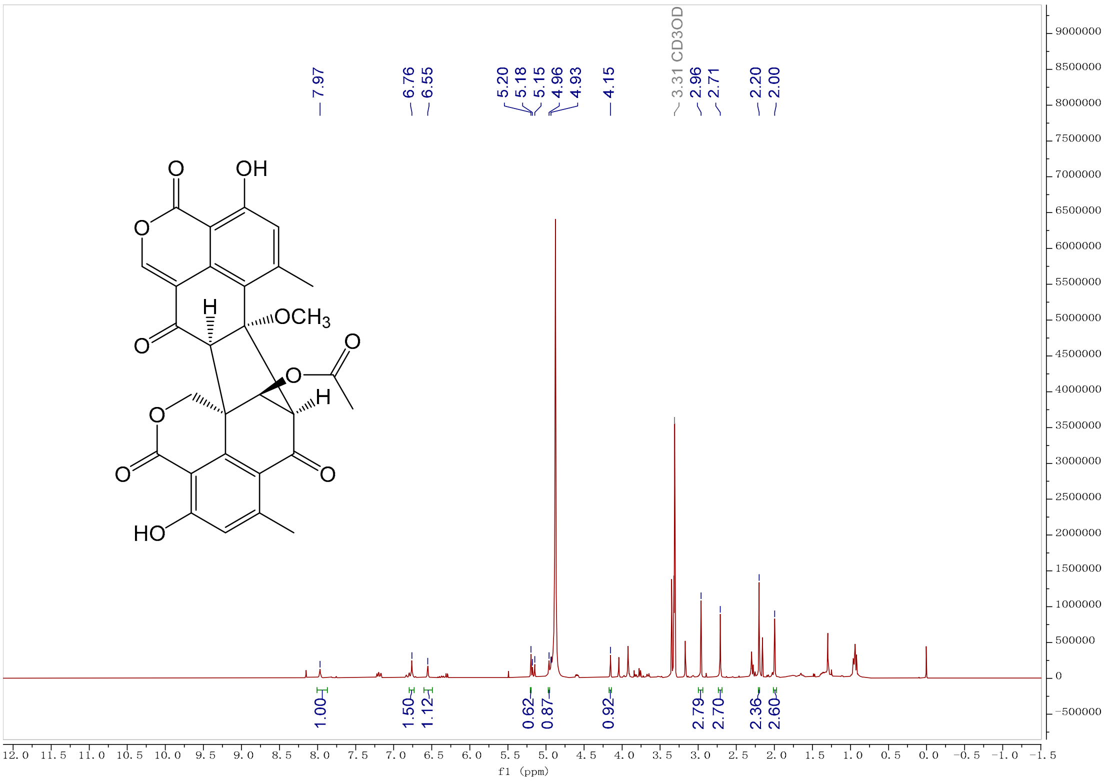

Figure S59 ^{1}H NMR spectrum of compound **19** (400 MHz, Acetone-d_6).

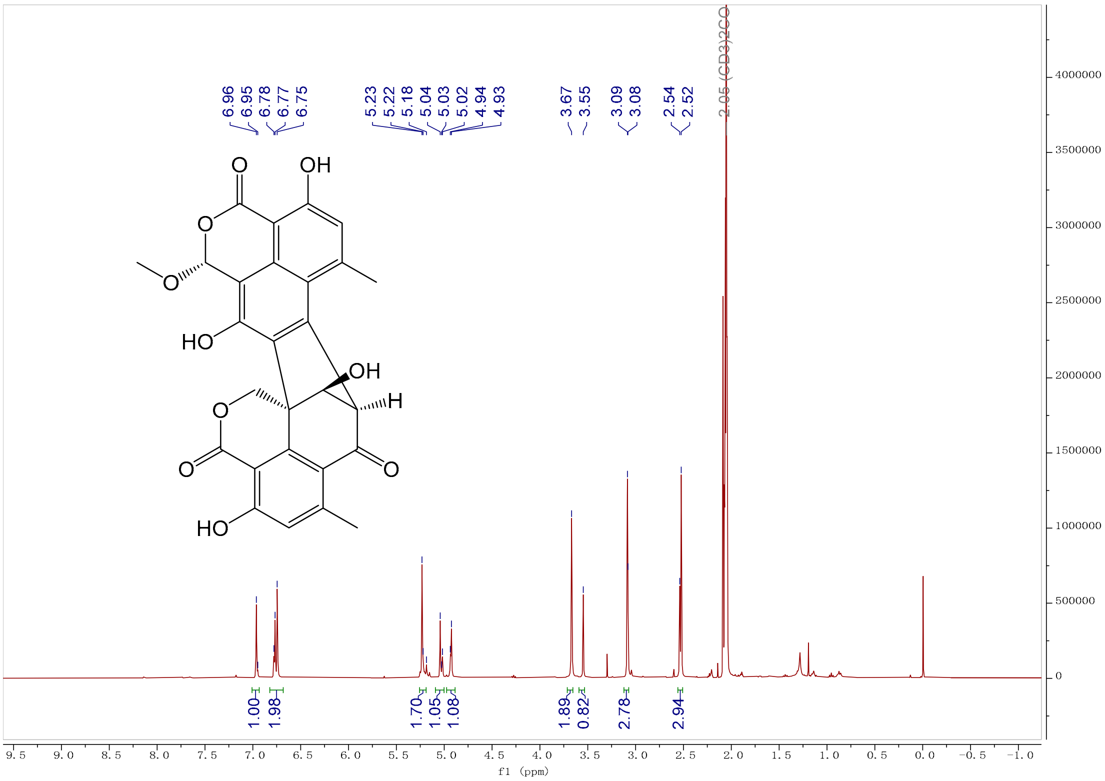

Figure S60 ^{1}H NMR spectrum of compound **20** (400 MHz, Acetone-d_6).

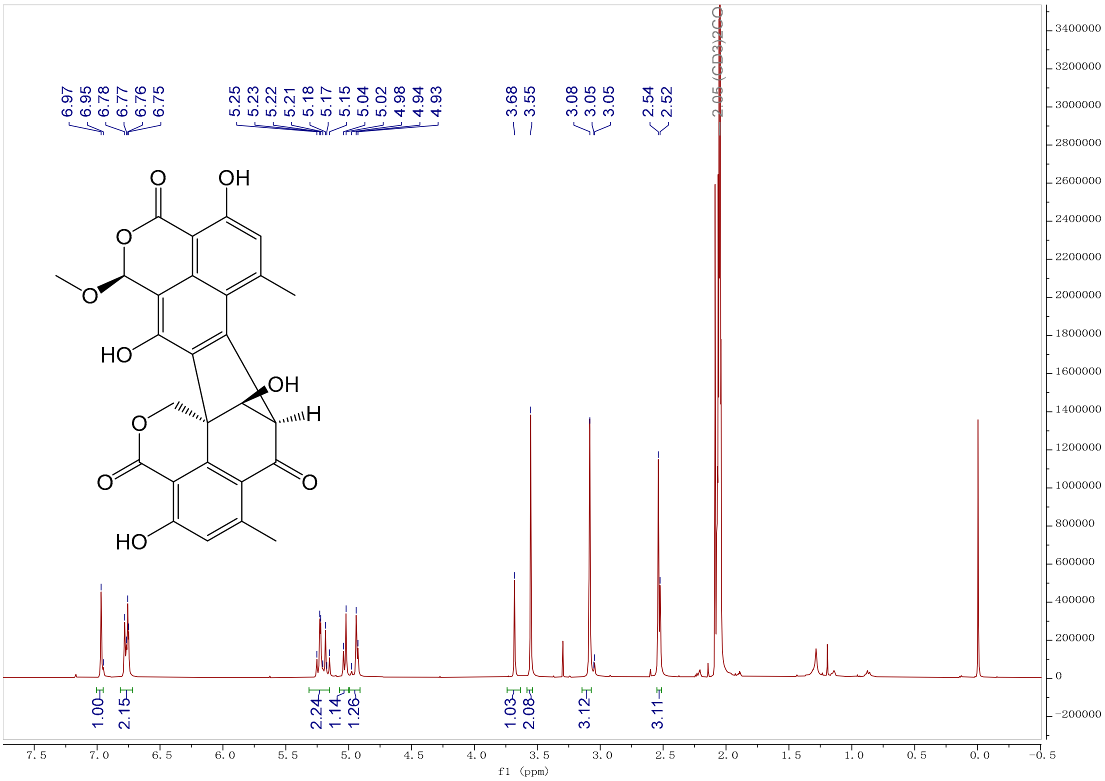

Figure S61 ^{1}H NMR spectrum of compound **21** (400 MHz, Acetone-d_6).

The ITS sequence comparison results of the sample were *Talaromyces pinophilus* (or the same genus) The ITS sequencing results and NCBI comparison results were as follows:

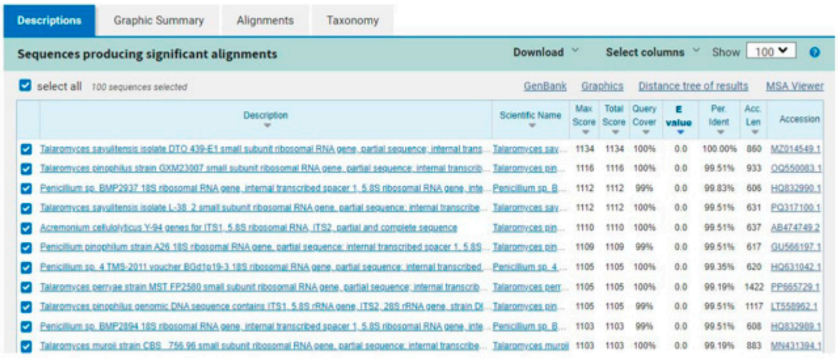

Descriptions | Graphic Summary | Alignments | Taxonomy

Sequences producing significant alignments — Download — Select columns — Show 100

select all 100 sequences selected — GenBank Graphics Distance tree of results MSA Viewer

Description	Scientific Name	Max Score	Total Score	Query Cover	E value	Per. Ident	Acc. Len	Accession
Talaromyces sayulitensis isolate DTO 439-E1 small subunit ribosomal RNA gene, partial sequence; internal trans...	Talaromyces say...	1134	1134	100%	0.0	100.00%	860	MZ014549.1
Talaromyces pinophilus strain GXM23007 small subunit ribosomal RNA gene, partial sequence; internal transcrib...	Talaromyces pin...	1116	1116	100%	0.0	99.51%	933	OQ550083.1
Penicillium sp. BMP2937 18S ribosomal RNA gene, internal transcribed spacer 1, 5.8S ribosomal RNA gene, inte...	Penicillium sp. B...	1112	1112	99%	0.0	99.83%	606	HQ832990.1
Talaromyces sayulitensis isolate L-38_2 small subunit ribosomal RNA gene, partial sequence; internal transcribe...	Talaromyces say...	1112	1112	100%	0.0	99.51%	631	PQ317100.1
Acremonium cellulolyticus Y-94 genes for ITS1, 5.8S ribosomal RNA, ITS2, partial and complete sequence	Talaromyces pin...	1110	1110	100%	0.0	99.51%	637	AB474749.2
Penicillium pinophilum strain A26 18S ribosomal RNA gene, partial sequence; internal transcribed spacer 1, 5.8S...	Talaromyces pin...	1109	1109	99%	0.0	99.51%	617	GU566197.1
Penicillium sp. 4 TMS-2011 voucher BGd1p19-3 18S ribosomal RNA gene, partial sequence; internal transcribed...	Penicillium sp. 4...	1105	1105	100%	0.0	99.35%	620	HQ631042.1
Talaromyces pernyae strain MST FP2580 small subunit ribosomal RNA gene, partial sequence; internal transcrib...	Talaromyces perr...	1105	1105	100%	0.0	99.19%	1422	PP665729.1
Talaromyces pinophilus genomic DNA sequence contains ITS1, 5.8S rRNA gene, ITS2, 28S rRNA gene, strain DI...	Talaromyces pin...	1105	1105	99%	0.0	99.51%	1117	LT558962.1
Penicillium sp. BMP2894 18S ribosomal RNA gene, internal transcribed spacer 1, 5.8S ribosomal RNA gene, inte...	Penicillium sp. B...	1103	1103	99%	0.0	99.51%	608	HQ832989.1
Talaromyces muroii strain CBS_756.96 small subunit ribosomal RNA gene, partial sequence; internal transcribe...	Talaromyces muroi	1103	1103	100%	0.0	99.19%	883	MN431394.1

Figure S62 The fungus ITS gene sequence.

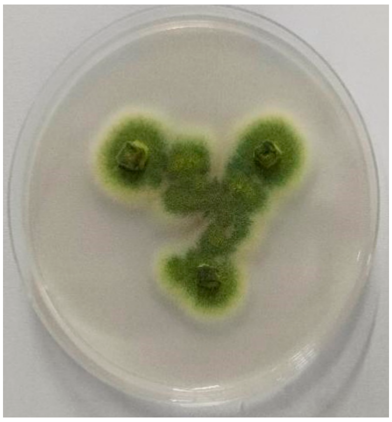

Figure S63 Plate image of the fungus.

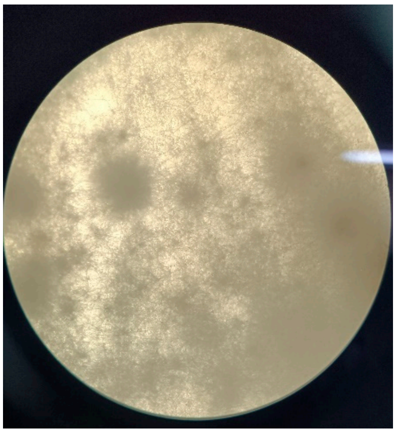

Figure S64 Microscopic images of the fungus.

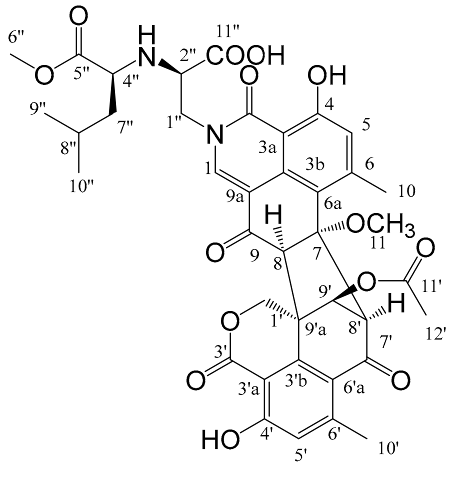

Table S1 NMR data of Talauxamide A (**1**) in $CDCl_3$

NO.	δ_C, type	δ_H, mult (*J* in Hz)	HMBC	COSY	NOESY
1	135.92, CH	8.01, s	3a、3b、9、1''		
3	165.29, C				
3a	106.94, C				
3b	134.67, C				

4	161.62, C				
5	118.45, CH	6.76, s	3a、4、10		
6	149.56, C				
6a	116.76, C				
7	88.63, C				
8	63.99, CH	4.01, s	3b、6a、1'、9'a		1'β, 11
9	194.41, C				
9a	111.51, C				
10	21.94, CH_3	2.70, s	6、6a		
11	51.73, CH_3	2.96, s	7		8
1'	71.51, CH_2	5.10, d (14.8)	3'、3'b、9'		8
		4.73, d (14.8)			9'
3'	167.42, C				
3'a	104.90, C				
3'b	142.97, C				
4'	164.73, C				
5'	121.24, CH	6.50, s	3'a、6'a、10'		
6'	151.65, C				
6'a	121.29, C				
7'	191.23, C				
8'	67.98, CH	4.14, s	6'a、9'a		9'
9'	78.82, CH	5.20, s	7'、11'		1'α, 8'
9'a	51.53, C				
10'	22.59, CH_3	2.05, s			
11'	169.79, C				
12'	21.11, CH_3	2.23, s			
1''	60.11, CH_2	4.07, m		2''	
		4.21, m			
2''	61.57, CH	5.31, t	11''	1''	
NH		6.72		4''	
4''	51.62, CH	4.66, m		7''	
5''	174.15, C				
6''	52.88, CH	3.80, s	5''		
7''	41.04, CH_2	1.62, m		4''	
8''	25.06, CH	1.66, m		9''	
9''	22.89, CH_3	0.95, overlap		8''	
10''	22.05, CH_3	0.95, overlap	7''	8''	
11''	167.03, C				
4-OH		12.07, s	3a、4、5		
4'-OH		11.58, s			

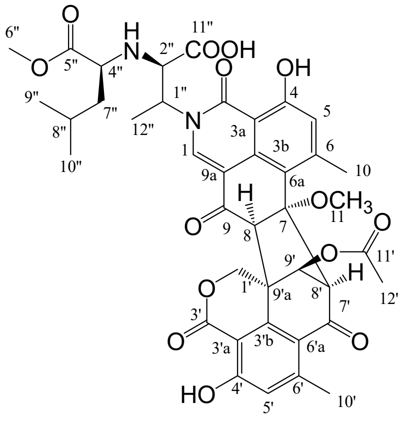

Table S2 NMR data of Talauxamide B (**2**) in $CDCl_3$

NO.	δ_C, type	δ_H, mult (*J* in Hz)	HMBC	COSY	NOESY
1	136.28, CH	8.14, s	3、3b、9		9'、1''
3	165.77, C				
3a	106.81, C				
3b	134.74, C				
4	161.61, C				
5	118.38, CH	6.76, s	3a、4、6a、10		
6	149.69, C				
6a	116.80, C				
7	88.56, C				
8	63.97, CH	4.00, s	6a、7、9、9a、1'、3'b、9'a		11、1'β
9	193.17, C				
9a	111.32, C				
10	22.05, CH_3	2.71, s	6、6a		
11	51.79, CH_3	2.95, s	7		8
1'	71.46, CH_2	5.08, d (13.2)	8、3'、3'b、		9'
		4.75, d (13.2)	9'、9'a		8
3'	167.59, C				
3'a	104.95, C				
3'b	143.05, C				
4'	164.68, C				
5'	120.97, CH	6.50, s, 1H	3'a、4'、6'a		
6'	151.51, C				
6'a	121.25, C				
7'	191.27, C				
8'	67.98, CH	4.14, s, 1H	7、8、9a、1'、7'、6'a、9'、9'a		
9'	78.85, CH	5.20, s, 1H	7、8、3'、3'b、7’		1'α、8'
9'a	51.53, C				

10'	22.61, CH_3	2.05, s, 3H	6'、6'a		
11'	169.83, C				
12'	21.11, CH_3	2.23, s, 3H	11'		
1"	67.06, CH	4.51, m, 1H		12"	
2"	62.70, CH	5.23, t, 1H			
3"	NH	6.76, d, 1H		4"	
4"	51.60, CH	4.64, m, 1H	7"	3"、7"	
5"	173.95, C				
6"	52.83, CH_3	3.79, s, 3H	5"		
7"	40.91, CH_2	1.61, m, 2H	4"	4"	
8"	25.15, CH	1.69, m, 1H	7"		
9"	22.84, CH_3	0.95, m, 3H	7"、8"	8"	
10"	22.08, CH_3	0.95, m, 3H			
11"	166.70, C				
12"	19.85, CH_3	1.22, d, 3H	2"	1"	9'
4-OH		12.06, s			
4'-OH					

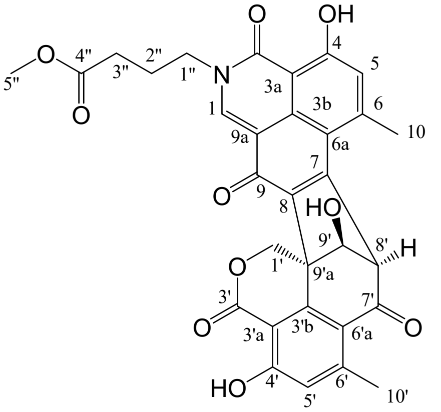

Table S3 NMR data of Talauxamide C (**3**) in Acetone-d_6

No.	δ_C, type	δ_H, mult (J in Hz)	HMBC	COSY	NOESY
1	143.86 CH	8.52, s	3、3b、9、9a、1"		
3	166.04 C				
3a	109.00 C				
3b	135.73 C				
4	164.84 C				
5	118.35 CH	6.72, s	3a、6a		
6	149.53 C				
6a	115.71 C				
7	152.83 C				
8	140.65 C				
9	178.36 C				

9a	115.01 C				
10	25.13 CH_3	2.99, s	5、6		
1'	70.46 CH_2	5.09, s	8、3'、3'a、3'b、9'		
3'	169.13 C				
3'a	105.29 C				
3'b	148.68 C				
4'	164.75 C				
5'	120.44 CH	6.78, s	3'a、6'a、10'		
6'	153.71 C				
6'a	118.25 C				
7'	192.23 C				
8'	67.66 CH	4.87, overlap	8、9'a		
9'	85.91 CH	4.86, overlap	6'a、7'		
9'a	50.52 C				
10'	23.77 CH_3	2.56, s	6'a		
1"	49.97 CH_2	4.26, m		2"	
2"	25.00 CH_2	2.07, t	4"	1"、3"	
3"	31.22 CH_2	2.42, t	1"	2"	
4"	173.34 C				
5"	51.71 CH_3	3.54, s	4"		
4-OH					
4'-OH					
9'-OH					

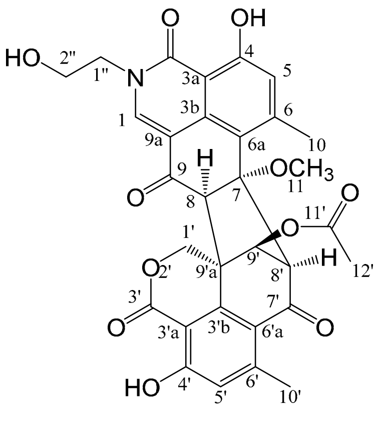

Table S4 NMR data of Talauxamide D (**4**) in Acetone-d_6

No.	δ_C, type	δ_H, mult (J in Hz)	HMBC	COSY	NOESY
1	135.32 CH	7.86, s	3、3b、9、2"		
3	164.10 C				
3a	107.03 C				
3b	137.40 C				
4	161.26 C				
5	117.08 CH	6.74, s	3a、4、6a、10、		

6	148.47 C			
6a	116.73 C			
7	88.48 C			
8	63.88 CH	4.01, s	11、1'、3'b	11、1'β
9	193.16 C			
9a	110.90 C			
10	21.08 CH_3	2.70, s	5、6	
11	51.21 CH_3	2.98, s	6a、7	8
1'	71.29 CH_2	5.05, d (16.0)	8、3'b	9'
		5.17, d (16.0)		8
3'	165.35 C			
3'a	105.21 C			
3'b	143.49 C			
4'	167.49 C			
5'	119.97 CH	6.53, s	3a'、6'a	
6’	150.69 C			
6'a	121.43 C			
7'	191.32 C			
8'	67.86 CH	4.14, overlap	8、6'a、9'a	9'
9'	78.95 CH	5.21, s	7、8、3'b、7'、11'	1'α、8'
9'a	51.21 C			
10'	21.25 CH_3	1.99, s	5'	
11'	169.47 C			
12'	20.01 CH_3	2.21, s	11'	
1''	59.14 CH_2	3.77, overlap	1、3	
		4.12, overlap		
2''	50.80 CH_2	3.78, overlap		
		4.10, overlap		
4-OH		12.56, s		
4'-OH				

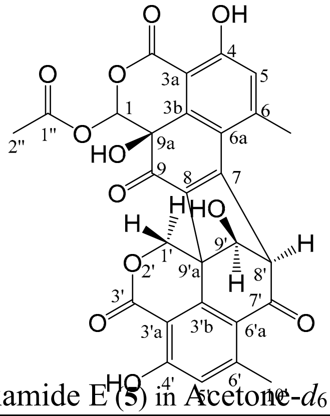

Table S5 NMR data of Talauxamide E (5) in Acetone-d_6

No.	C	H	HMBC	COS Y	NOESY

1	80.33 CH	5.44, dd	3、3b、1"、2"	
3	167.85 C			
3a	108.74 C			
3b	143.96 C			
4	162.86 C			
5	122.19 CH	6.97, s	3a、6a、10	
6	148.24 C			
6a	118.49 C			
7	156.57 C			
8	137.83 C			
9	192.92 C			
9a	67.83 C			
10	24.68 CH_3	2.95, s	6、6a	
1'	70.18 CH_2	5.06, d (12.3)	3'、3'b、9'、9'a	9'
		4.86, d (12.3)		
3'	168.93 C			
3'a	105.18 C			
3'b	148.59 C			
4'	164.90 C			
5'	120.54 CH	6.78, s	3'a、6'a	
6'	153.76 C			
6'a	119.62 C			
7'	191.08 C			
8'	68.99 CH	4.77, s	8、6'a	
9'	86.06 CH	4.72, s	7、8、7'	1'α
9'a	50.28 C			
10'	23.77 CH_3	2.56, s	5'、6'a	
1"	170.22 C			
2"	36.57 CH_3	2.05, m	1、1"	
4-OH				
4'-OH				
